# Enantioselective *cis*-β-lactam synthesis by intramolecular C–H functionalization from enoldiazoacetamides and derivative donor–acceptor cyclopropenes†
†Electronic supplementary information (ESI) available: Experimental details of substrates preparation, catalytic experiments, identification of the products and crystal data for **3i**. CCDC 1018978. For ESI and crystallographic data in CIF or other electronic format see DOI: 10.1039/c4sc03991b



**DOI:** 10.1039/c4sc03991b

**Published:** 2015-01-28

**Authors:** Xinfang Xu, Yongming Deng, David N. Yim, Peter Y. Zavalij, Michael P. Doyle

**Affiliations:** a Key Laboratory of Organic Synthesis of Jiangsu Province , College of Chemistry , Chemical Engineering and Materials Science , Soochow University , Suzhou 215123 , China . Email: xinfangxu@suda.edu.cn ; Tel: +86 0512 65883612; b Department of Chemistry and Biochemistry , University of Maryland , College Park , Maryland 20742 , USA . Email: mdoyle3@umd.edu

## Abstract

β-Lactam derivatives are produced through intermediate donor–acceptor cyclopropene intermediates in high yield, exclusive *cis*-diastereoselectivity, and high enantiocontrol in a chiral dirhodium carboxylate catalyzed intramolecular C–H functionalization reaction of enoldiazoacetamides.

## Abstract 2

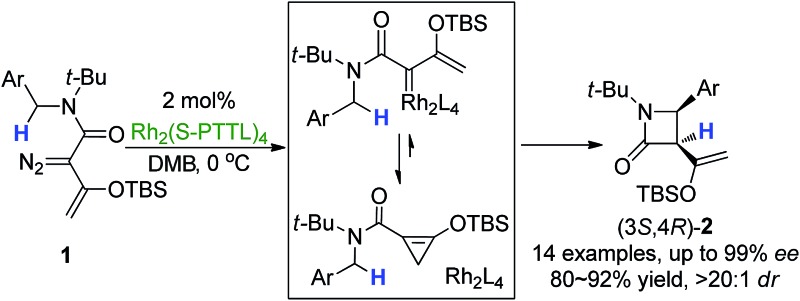
β-Lactam derivatives are produced through donor–acceptor cyclopropene intermediates in high yield with exclusive *cis*-diastereoselectivity, and high enantiocontrol.

## Introduction

The importance of β-lactam compounds (2-azetidinones) in biology and medicine is undisputed since the discovery of the antibiotic activity of the bicyclic penicillin in 1928,^1,2^ and monocyclic β-lactams that include the plasma cholesterol-lowering Ezetimibe^3^ also show biological activity.^4^ Considerable effort has been focused on chemical catalysis for the construction of β-lactams, including those through catalytic intramolecular amide N–H insertion reactions of diazo compounds,^5^ and asymmetric synthesis has been a primary focus.^2,6^ However, the same C–H functionalization methodology of diazo compounds that has provided exceptional selectivities in intermolecular reactions^7^ and intramolecular syntheses of γ-lactones and γ-lactams^8^ has been limited in efforts to synthesize β-lactams.
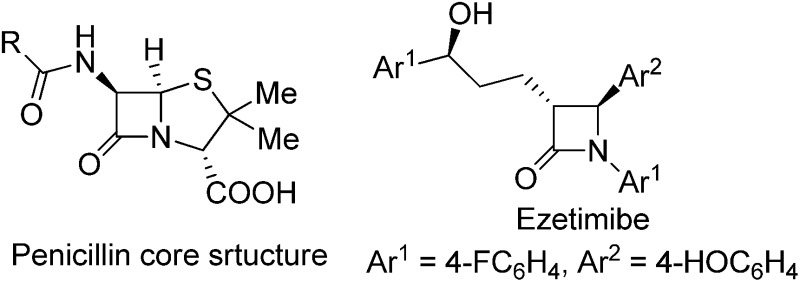


**Scheme 1:**
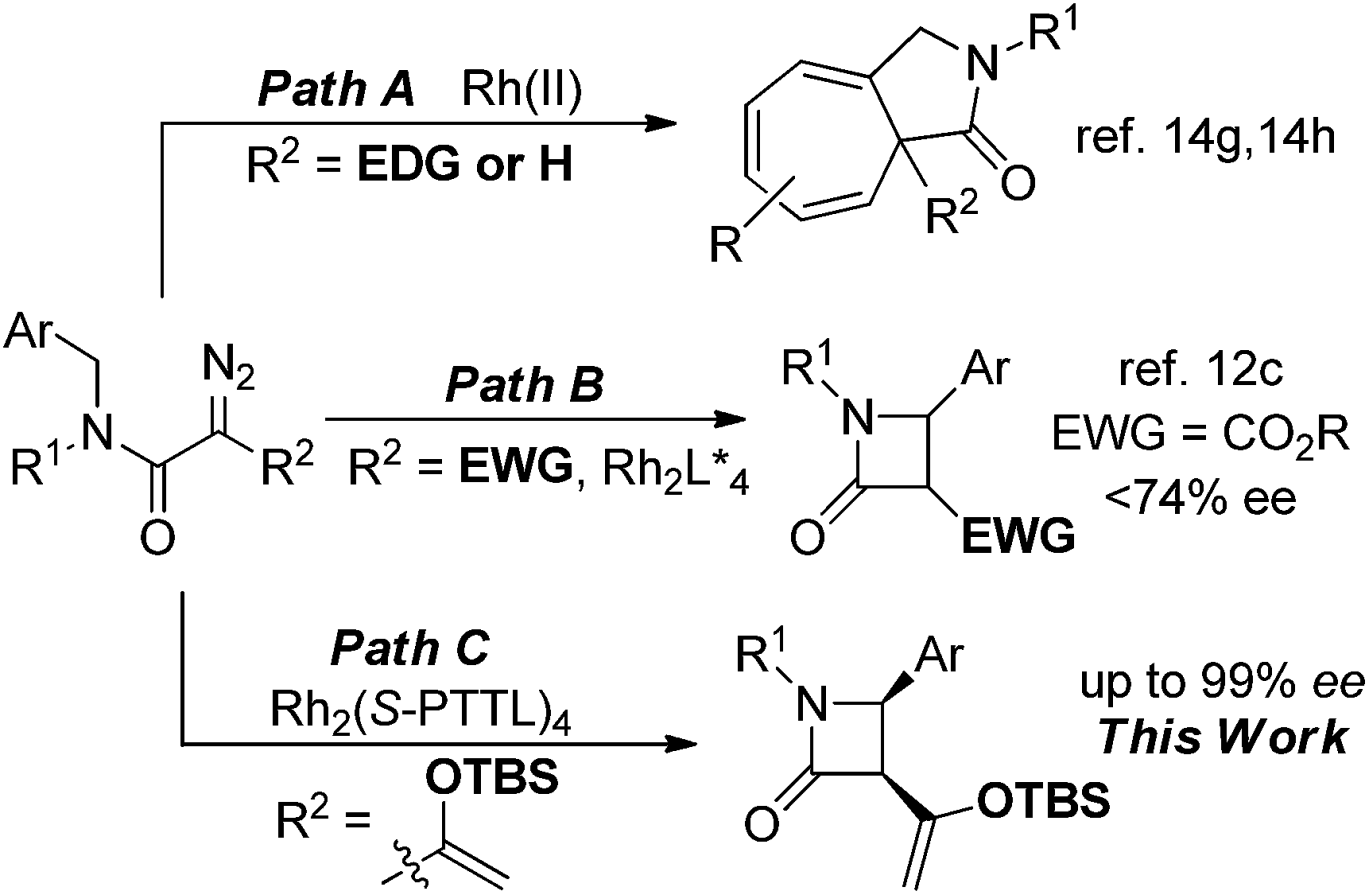
Diazoacetamide substrate dependence on chemoselectivity.

Direct intramolecular C–H functionalization of diazoacetamides catalyzed by transition metal catalysts is straightforward.^9^ The amide nitrogen activates the adjacent C–H bond for insertion. Although β-lactam formation by photocatalysis was described fifty years ago,^10^ and the first enantioselective process was reported in 1992,^2c,11^ there have only been a few examples that have overcome a majority of the electronic, steric, and conformational factors which control the selectivity of this process.^12,13^ Competing reactions include intramolecular cycloaddition to an aromatic ring of an aryl- or heterocycle attachment (Buchner reaction), addition to a carbon–carbon multiple bond of an allylic or propargylic system, or regioselective formation of a γ-lactam from C–H functionalization.^14^ Product selectivity is highly dependent on the diazo compound that is employed;^12,14^ for example, acceptor or donor–acceptor diazoamides form aromatic cycloaddition products when catalyzed by dirhodium catalysts (Scheme 1, Path A, R^2^ = H or EDG), but acceptor–acceptor diazoamides produce β-lactam products by a C–H functionalization reaction (Path B, R^2^ = EWG).

To achieve high selectivity in C–H functionalization reactions that form β-lactam products, acceptor–acceptor diazoamides (R^2^ = EWG) have been constructed to avoid side reactions, but high regio- and enantiocontrol (>90% ee) has only been demonstrated in constrained cyclic systems in which the aliphatic γ-position has been made inaccessible for insertion.^12a,b^ We have developed enoldiazoacetates as a new class of stable donor–acceptor diazo compounds with extensive applications for cycloaddition reactions.^15^ An attractive feature of this vinyldiazo compound in catalytic reactions with dirhodium(ii) carboxylates is the apparent donor–acceptor cyclopropene intermediate that serves as a resting state for the apparent reactive metal carbene intermediate.^16^ Could enoldiazoacetamides form intermediate donor–acceptor cyclopropenes and be precursors to donor–acceptor metal carbene intermediates on the pathway to C–H functionalization? Earlier work by Müller suggested that enantioselectivity in cyclopropanation reactions of styrene with an enoldiazoacetate is significantly greater than that with the corresponding diazoacetoacetate.^17^ We wish to report that asymmetric catalysis with donor–acceptor *N*-benzyldiazoamides proceeds through donor–acceptor cyclopropene intermediates to form *cis*-disubstituted β-lactams by intramolecular C–H functionalization in high yields and stereoselectivities (Path C).

## Results and discussion

In initial studies we selected the *N-tert*-butyl-*N*-(*p*-methoxy-benzyl)enoldiazoacetamide **1a** as a model substrate since previous studies have shown that the *tert*-butyl group fixed the reactant in the conformation in which the benzyl and diazo functional groups are *syn* to each other.^13^ In this conformation C–H insertion into the *tert*-butyl group is prevented, but aromatic cycloaddition into the anisyl group could be competitive with C–H insertion into its benzyl group, and indeed this competition was observed (Table 1). The Buchner product **2a** was dominant in reactions catalyzed by sterically unencumbered Rh_2_(OAc)_4_ or the electrophilic Rh_2_(pfb)_4_, but with the sterically restrictive Rh_2_(tpa)_4_ or Rh_2_(esp)_2_ the sole product was the *cis*-disubstituted β-lactam **3a**, formed by C–H insertion into the benzylic position. With asymmetric catalysts similar steric influences were operative so that increasing the steric bulk of the chiral Hashimoto dirhodium(ii) carboxylate catalyst ligand increased the **3a**/**2a** ratio and, also, enhanced enantioselectivity (entries 5–9) for both products. Both Rh_2_(*S*-PTTL)_4_ and Rh_2_(*S*-PTAD)_4_ gave β-lactam **3a** as the only product in high yield with 64% ee (entries 8 and 9). We chose Rh_2_(*S*-PTTL)_4_ for further optimization and, after screening solvents and reaction temperatures, the reaction carried out at 0 °C in 2,2-dimethylbutane (DMB) gave the optimum result with 85% isolated yield and 92% ee of **3a** (entry 17).^18^ The more Lewis acidic Rh_2_(*S*-TCPTTL)_4_ gave **3a** in higher yield but slightly lower % ee. Chiral dirhodium carboxamidates were not effective as catalysts for this transformation, but the more reactive Rh_2_(*S*-DOSP)_4_ produced a mixture of **2a** and **3a** with low enantioselectivities (entry 5).

**Table 1:** Optimization of reaction conditions for the enantioselective C–H functionalization of enlodiazoacetamide **1a**^*a*^

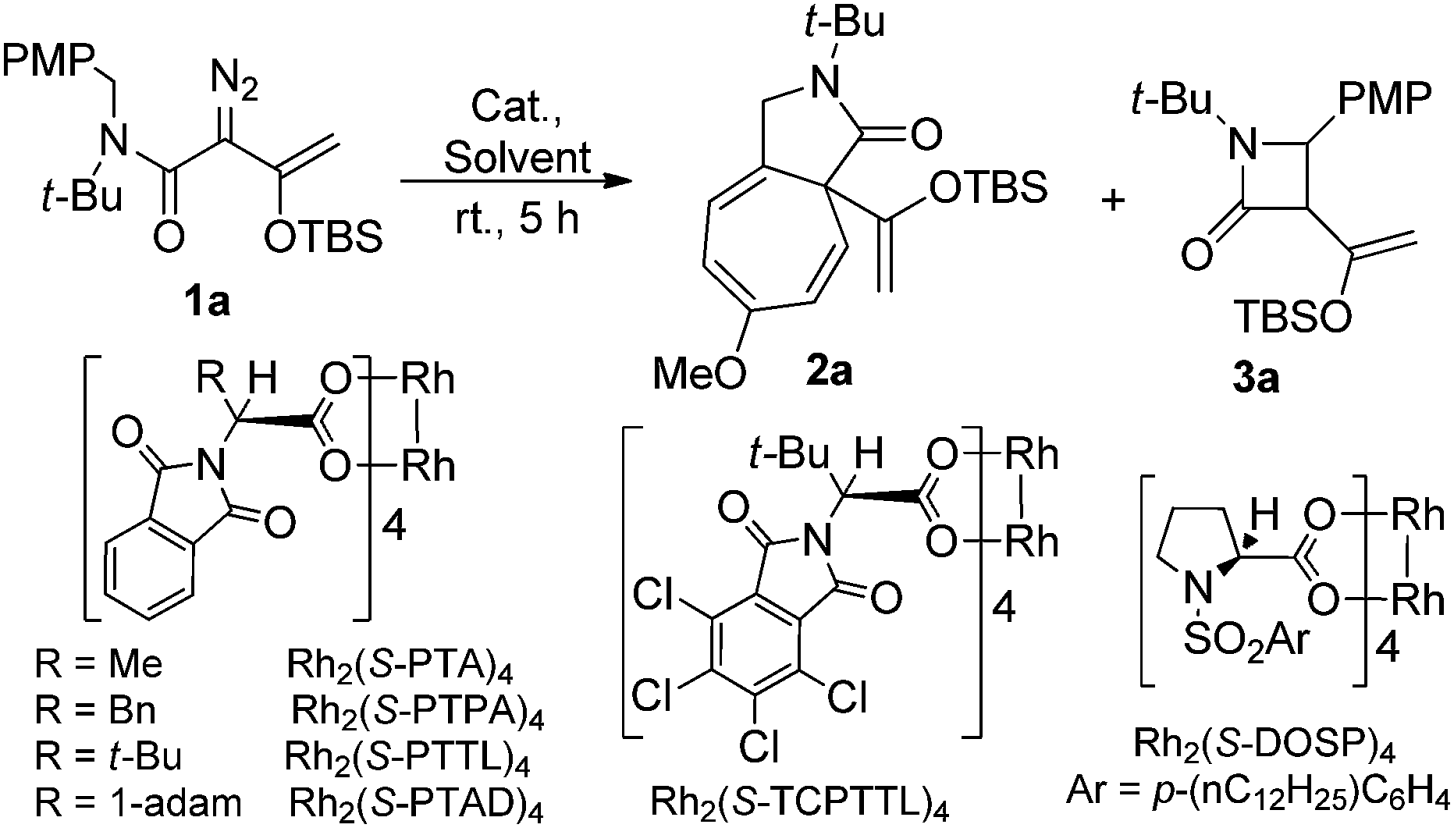					
Entry	Rh(ii)	Solvent	**2a** : **3a**^*e*^	Yield^*f*^, (%) **2a** + **3a**	ee^*g*^ (%) **2a**/**3a**
1^*b*^	Rh_2_(OAc)_4_	DCCl_3_	80 : 20	87	—/—
2^*b*^	Rh_2_(pfb)_4_	DCCl_3_	80 : 20	89	—/—
3^*b*^	Rh_2_(tpa)_4_	DCCl_3_	<5 : 95	92	—/—
4^*b*^	Rh_2_(esp)_2_	DCCl_3_	<5 : 95	87	—/—
5^*b*^	Rh_2_(*S*-DOSP)_4_	DCCl_3_	37 : 63	90	27/10
6^*b*^	Rh_2_(*S*-PTA)_4_	DCCl_3_	25 : 75	92	35/30
7^*b*^	Rh_2_(*S*-PTPA)_4_	DCCl_3_	33 : 67	90	53/41
8^*b*^	Rh_2_(*S*-PTTL)_4_	DCCl_3_	<5 : 95	93	—/64
9^*b*^	Rh_2_(*S*-PTAD)_4_	DCCl_3_	<5 : 95	93	—/64
10	Rh_2_(*S*-PTTL)_4_	DCM	<5 : 95	89	—/65
11	Rh_2_(*S*-PTTL)_4_	DCE	<5 : 95	88	—/68
12	Rh_2_(*S*-PTTL)_4_	Toluene	<5 : 95	76	—/75
13	Rh_2_(*S*-PTTL)_4_	CF_3_Ph	<5 : 95	72	—/67
14	Rh_2_(*S*-PTTL)_4_	Cyclohexane	<5 : 95	90	—/82
15	Rh_2_(*S*-PTTL)_4_	TBME	<5 : 95	91	—/71
16	Rh_2_(*S*-PTTL)_4_	DMB	<5 : 95	83	—/88
17^*c*^	Rh_2_(*S*-PTTL)_4_	DMB	<5 : 95	85	—/92
18^*d*^	Rh_2_(*S*-PTTL)_4_	DMB	<5 : 95	82	—/91
19^*c*^	Rh_2_(*S*-TCPTTL)_4_	DMB	<5 : 95	88	—/89

^*a*^Reactions were carried out at room temperature on a 0.2 mmol scale in 1.0 mL solvent with 2.0 mol% dirhodium catalyst in 5 hours.

^*b*^Reactions were carried out at room temperature on a 0.2 mmol scale in 0.5 mL DCCl_3_ with 2.0 mol% dirhodium catalyst in an NMR tube.

^*c*^The reaction was carried out at 0 °C in 3 hours.

^*d*^The reaction was carried out at –20 °C overnight.

^*e*^The ratio was determined by integration of characteristic ^1^H NMR absorptions from the spectrum of the reaction mixture.

^*f*^Isolated yield after chromatography.

^*g*^Enantioselectivity was determined by chiral HPLC analysis, see ESI for details. TBME = *tert*-butyl methyl ether; DMB = 2,2-dimethylbutane.

The observed high enantioselectivity obtained from catalytic intramolecular C–H insertion of **1a** was not observed with the corresponding diazoacetamide. β-Lactam **5a** was obtained in only 60% ee for when diazoacetamide **4a** was reacted under the same conditions (eqn (1)) and, in contrast to the exclusive *cis*-selectivity observed in the formation of β-lactam **3a**, **5a** was formed with exclusive *trans*-selectivity.^19^ The *trans* β-lactam product was also obtained in high yield in reactions catalyzed by achiral [RuCl_2_(*p*-cymene)]_2_ reported by Chi.^14f^ The reason for this difference in diastereoselectivity is probably isomerization of the β-ketoamide and, indeed, *cis*-**3c** is converted to *trans*-**5c** upon hydrolysis (eqn (2)) without loss of enantioselectivity.1
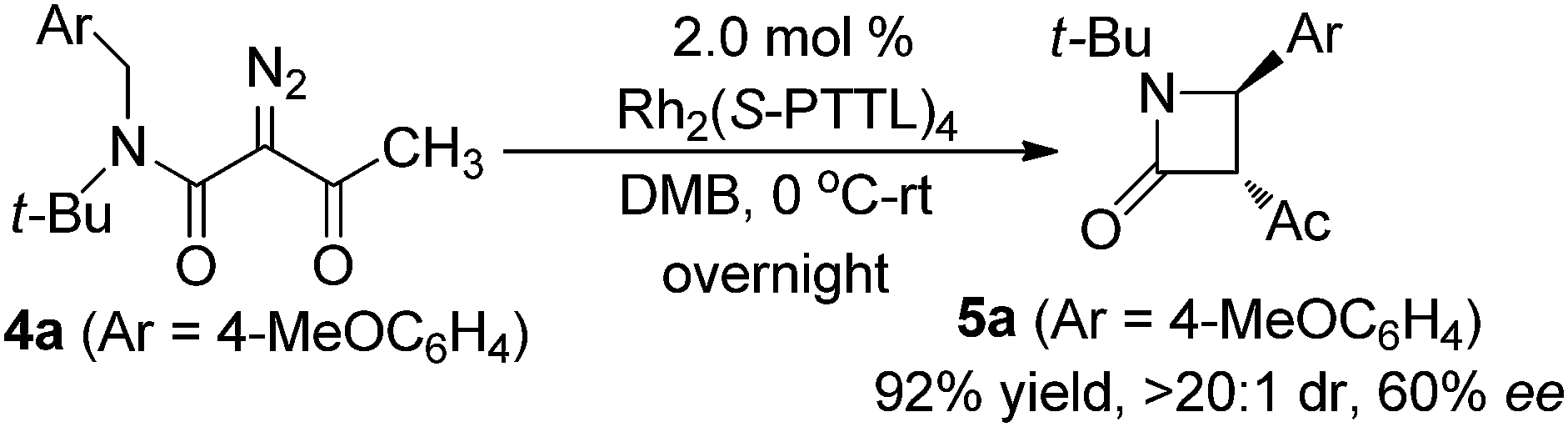

2
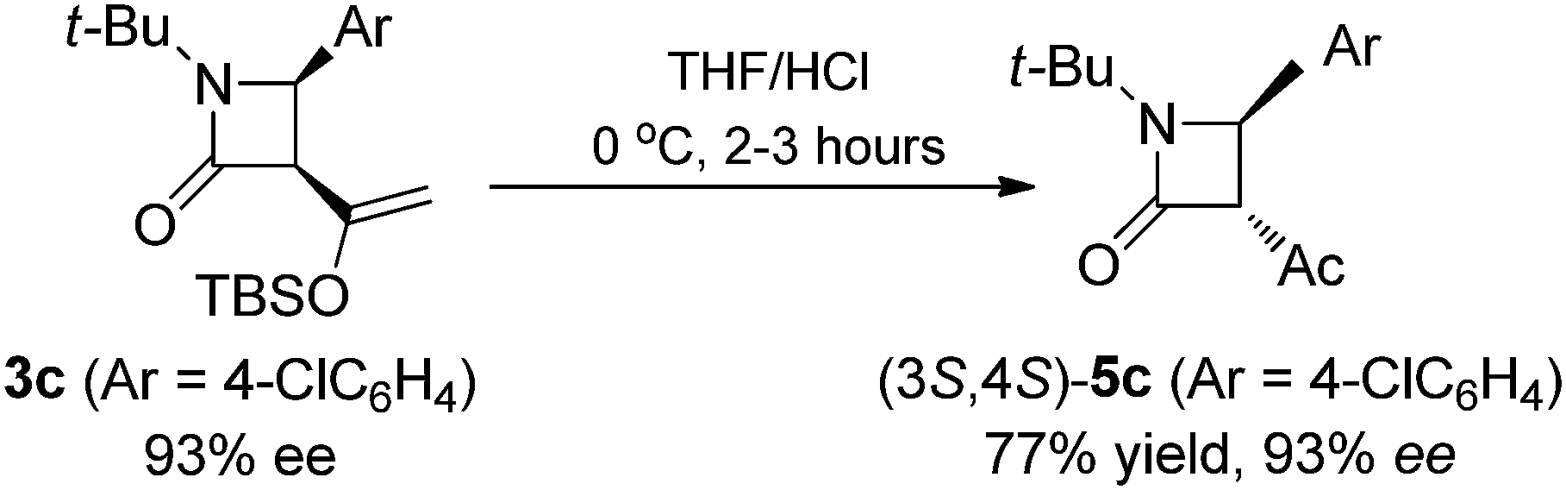



The scope of the C–H functionalization reaction of representative diazoacetamides **1** was investigated with Rh_2_(*S*-PTTL)_4_ catalysis under the optimum conditions established for **1a** (Table 2). In all examples, *cis*-β-lactam **3** was generated exclusively and in high yield (80–92%) and with high enantioselectivities (83–99% ee). The % ee of β-lactam **3** was lower when strong electron-withdrawing substituents were on the aromatic ring, and these reactions also required longer times for completion than did enoldiazoacetamides with electron-donating substituents (entries 6 and 7). Lower enantioselectivities were obtained with substrates having *m*- or *o*-substituents, and the lowered % ee was independent of a second substituent at the *para* position (entries 10 and 11) or of the size of the *ortho* substituent (entries 12 and 13). Using the *N*,*N*-diisopropylamide instead of the benzyl-*t*-butylamide also resulted in a β-lactam with lower enantioselectivity but good yield. Rh_2_(*S*-NTTL)_4_ gave higher enantiocontrol by 7% ee in the reaction of unsubstituted enoldiazo acetamide **1b** (entry 2), but the same or lower enantioselectivities were observed in reactions with **1a**, **1f**, **1g**, and **1k**. Product mixtures were obtained when Ar = the heteroaryl 3-furanyl group and included products from [3 + 4]-cycloaddition.^7^ It is noteworthy that β-lactam **3i** with the *p*-dimethylamino substituent was obtained with 99% ee in 80% yield (entry 9). And TIPS protected substrate **1c′** gave similar results as **1c** (entry 3 *vs.* entry 15). When an *N*-aryl substituent was used instead of the *t*-butyl group diazo compound **1o**, the reaction gave both Buchner reaction and C–H functionalization products in a 1 : 2 ratio with 78% total yield, and β-lactam **3o** was formed with 71% ee (eqn (3)).3
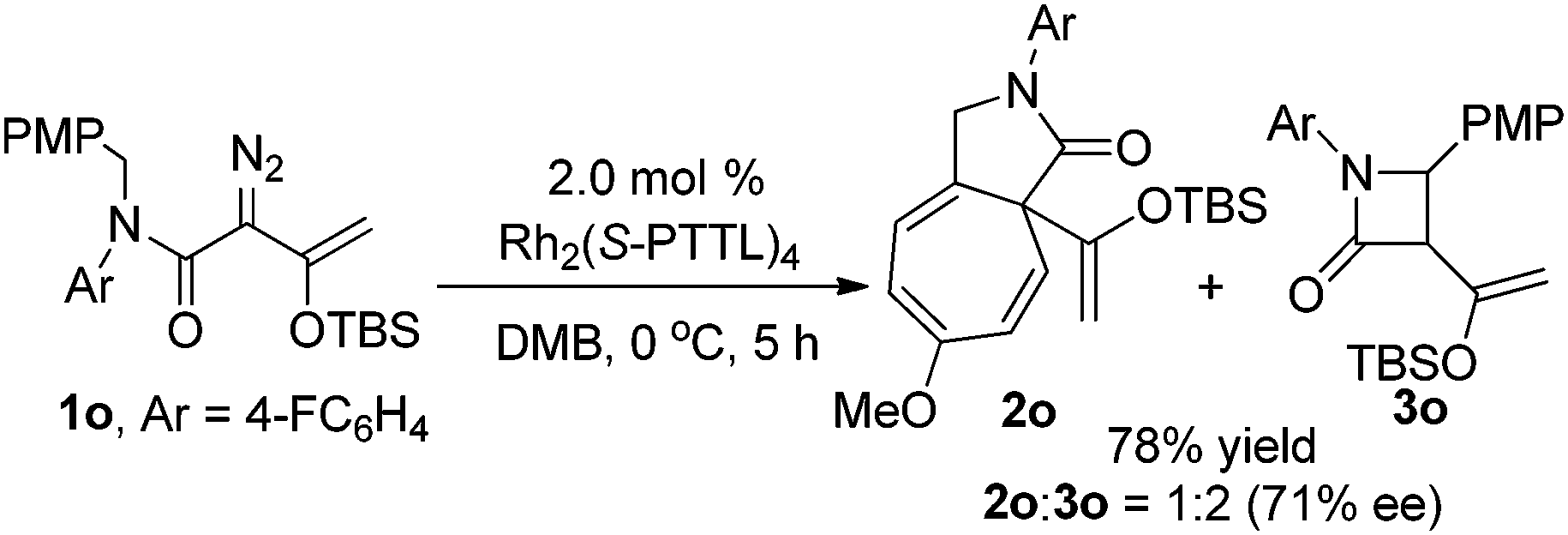



**Table 2:** Enantioselective C–H functionalization of **1**^*a*^

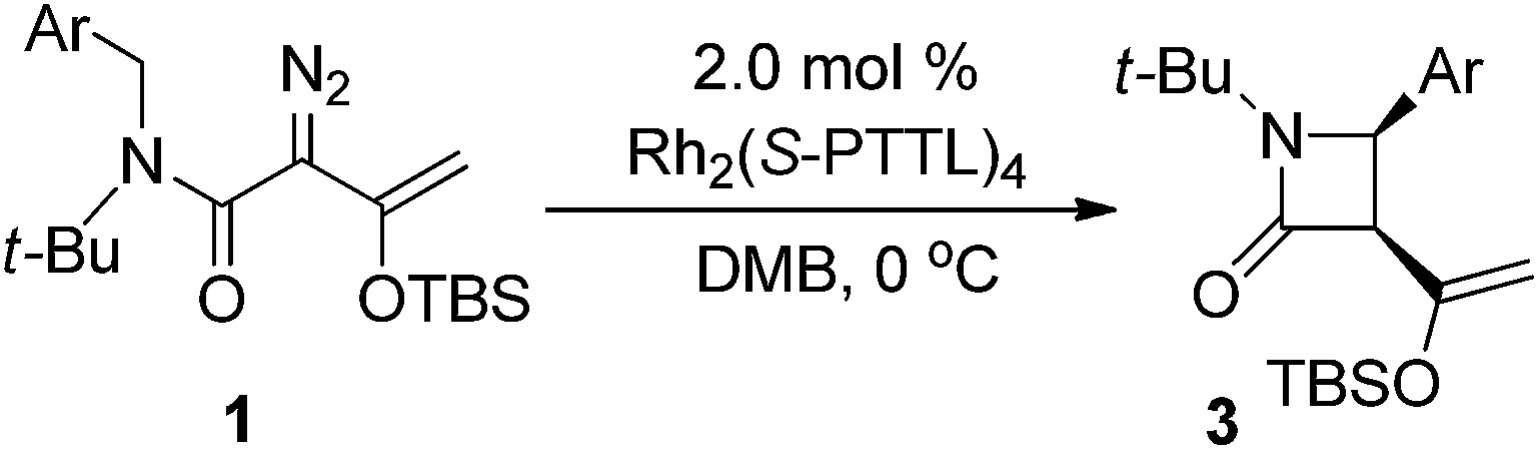					
Entry	Ar (**1**)	*t* (hours)	**3**	Yield^*c*^ (%)	ee^*d*^ (%)
1	4-MeO_6_H_4_ (**1a**)	3	**3a**	85	92
2	C_6_H_5_(**1b**)	12	**3b**	82 (81)^*b*^	80 (87)^*b*^
3	4-ClC_6_H_4_ (**1c**)	5	**3c**	88	93
4	4-MeC_6_H_4_ (**1d**)	5	**3d**	92	93
5	4-FC_6_H_4_ (**1e**)	5	**3e**	88	91
6	4-BrC_6_H_4_ (**1f**)	12	**3f**	89	89
7	4-NO_2_C_6_H_4_ (**1g**)	12	**3g**	88	83
8	4-PhC_6_H_4_ (**1h**)	5	**3h**	89	91
9	4-Me_2_NC_6_H_4_ (**1i**)	5	**3i**	80	99
10	3,4-(MeO)_2_C_6_H_3_ (**1j**)	5	**3j**	81	77
11	3-MeOC_6_H_4_ (**1k**)	5	**3k**	92	78
12	2-MeOC_6_H_4_ (**1l**)	12	**3l**	85	25
13	1-Naphthyl (**1m**)	5	**3m**	85	24
14^*e*^	*N*,*N*-Diisopropyl (**1n**)	5	**3n**	81	67
15^*f*^	4-ClC_6_H_4_ (**1c′**)	5	**3c′**	84	92

^*a*^Reactions were carried out on a 0.2 mmol scale in 1.0 mL DMB with 2.0 mol% Rh_2_(*S*-PTTL)_4_.

^*b*^Results in parentheses was catalyzed by Rh_2_(*S*-NTTL)_4_.

^*c*^Isolated yield.

^*d*^Enantioselectivity was determined by chiral HPLC analysis, see ESI for details.

^*e*^ *N*,*N*-Diisopropyl instead of *N-tert*-butyl-*N*-benzyl diazoamide was used.

^*f*^TIPS protection instead of TBS was used.

**Scheme 2:**
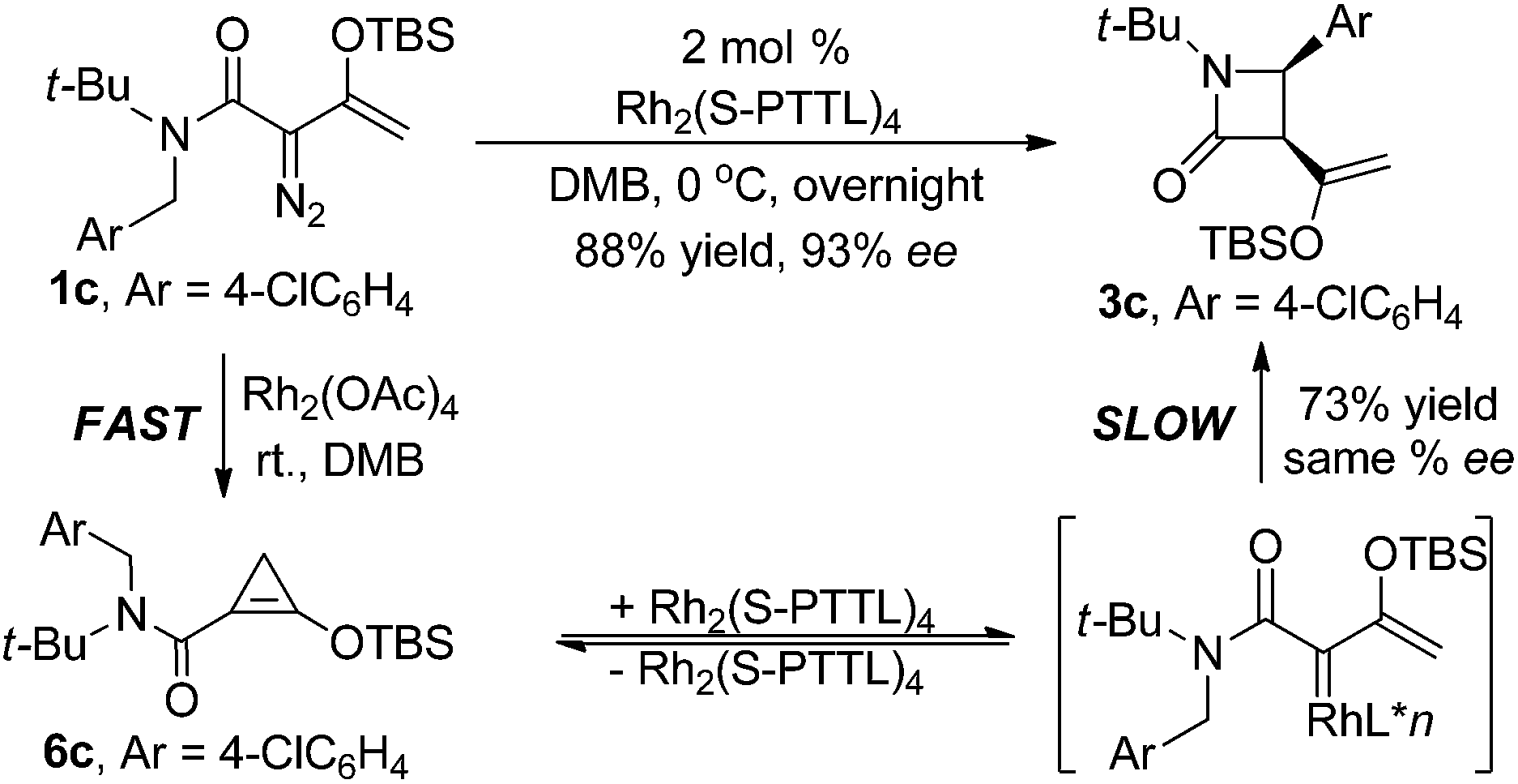
Donor–acceptor cyclopropenes are reaction intermediates in metal carbene formation.

As is suggested by the reaction conditions, these reactions are relatively rapid. However, close spectroscopic inspection of reactions with **1c** revealed that donor–acceptor cyclopropene **6c** was formed at a much faster rate than was the product from C–H insertion. To determine if the donor–acceptor cyclopropene is a precursor to the donor–acceptor chiral metal carbene intermediate whose intramolecular C–H insertion produces highly enantiomerically enriched β-lactam, reaction of **1c** was performed in DMB with 2.0 mol% Rh_2_(OAc)_4_ and at 5 min was filtered through Celite to remove the Rh_2_(OAc)_4_. Spectral analysis of the residue showed <1% **1c**, 8 ± 1% **3c**, and 92 ± 2% donor–acceptor cyclopropene **6c**. This mixture was then submitted to the same reaction conditions as reported in Table 2 with Rh_2_(*S*-PTTL)_4_ catalysis to produce **3c** in 73% isolated yield and 83 ± 3% ee. Subtracting racemic product formed from Rh_2_(OAc)_4_ gives **3c** formed from donor–acceptor cyclopropene **6c** with the same selectivity as that formed from **1c** (Scheme 2).

That donor–acceptor cyclopropene **6** can serve as a precursor to an intermediate metal carbene of dirhodium(ii) which undergoes C–H insertion prompted us to investigate if other transition metals, particularly those of copper(i) and silver(i), could undergo the same transformation. Although both of these catalytically active metal ions are known to form metal carbenes directly from diazo compounds,^7,8,20^ they undergo Lewis acid catalyzed reactions with enoldiazoacetates,^15^ and they are distinctly different from dirhodium(ii) carboxylates in cycloaddition reactions with nitrones.^18*c*^ Since C–H insertion is notably unique to metal carbenes, reactions of metal catalysts with enoldiazoacetamides or their derivative cyclopropenes would be a demonstration of metal carbene involvement with these catalysts. To undertake this investigation, reactions were performed on both enoldiazoacetamide **1c** and cyclopropene **6c** that was prepared from **1c** by treatment with Rh_2_(OAc)_4_ in DMB as previously described, and these results are described in Table 3. The copper(i) and silver(i) catalysts are distinctly different from each other and from Rh_2_(OAc)_4_ in their reactions with enoldiazoacetamide **1c**: aromatic cycloaddition is favored over C–H-insertion in reactions of **1c** in the catalyst order: Ag(SbF_6_) > Cu(MeCN)_4_PF_6_ > Rh_2_(OAc)_4_ (entries 1 and 2); and this difference is also reflected in the results from reactions with cyclopropene **6c**. Surprisingly, the Cu(MeCN)_4_PF_6_ and AgSbF_6_ catalyzed reactions with enoldiazoacetamide **1c** provide more of the aromatic cycloaddition product, which is reported to be due to a more electrophilic metal carbene intermediate,^13^ than do their reactions with donor–acceptor cyclopropene **6c**. The observed differences in the **2c** : **3c** ratios from reactions with **1c** and **6c** suggest that there may be some dependence on the carbene source among the catalysts employed for aromatic cycloaddition and C–H insertion, and that donor–acceptor cyclopropene **6c** and enoldiazoacetate **1c** may not form the same conformationally identical metal carbene intermediate. Use of a box ligand effectively inhibits dinitrogen extrusion from enoldiazoacetamide **1c**, but metal carbene formation from cyclopropene **6c** occurs without this limitation (entries 3 and 5). Other Lewis acids or under the thermal condition did not give any Buchner reaction or C–H insertion product, and only slowly decomposition of **6c** was observed (entries 8–11).

**Table 3:** Comparison of catalysts in C–H insertion and aromatic cycloaddition reactions of **1c** and **6c**^*a*^

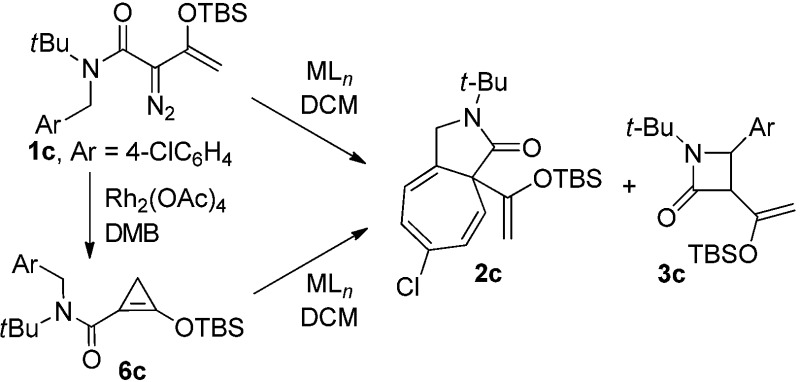						
Entry	Catalyst	Reactant	*t* (hours)	**2c** : **3c**	Yield^*d*^ (%) **2c** + **3c**	ee^*e*^ (%) **3c**
1^*b*^	AgSbF_6_	**1c**	12	>95 : 5	87	—
2^*b*^	Cu(MeCN)_4_PF_6_	**1c**	12	85 : 15	85	—
3^*c*^	Cu(MeCN)_4_PF_6_/(*S*)-*t*-BuBox	**1c**	48	<5 : 95	10	28
4^*b*^	Cu(MeCN)_4_PF_6_	**6c**	12	75 : 25	87	—
5^*c*^	Cu(MeCN)_4_PF_6_/(*S*)-*t*-BuBox	**6c**	12	<5 : 95	89	30
6^*b*^	AgSbF_6_	**6c**	12	85 : 15	82	—
7^*c*^	AgSbF_6_/(*S*)-*t*-BuBox	**6c**	12	75 : 25	91	24
8	Sc(OTf)_3_	**6c**	12	—	NR^*f*^	—
9	La(OTf)_3_	**6c**	12	—	NR^*f*^	—
10	BF_3_·Et_2_O	**6c**	12	—	NR^*f*^	—
11^*g*^	(—)	**6c**	12	—	NR^*f*^	—

^*a*^Reactions were carried out on a 0.2 mmol scale in 1.0 mL DCM.

^*b*^Reactions were carried out with 10 mol% Lewis acid catalyst.

^*c*^Reactions were carried out with 10 mol% Lewis acid catalyst and 12 mol% ligand.

^*d*^Isolated yield.

^*e*^The enantioselectivity was determined by chiral HPLC analysis, see ESI for details.

^*f*^Neither **2c** nor **3c** was observed, and only slowly decomposition of **6c** was observed.

^*g*^The reaction was carried out in 70 °C.

The assignment of relative stereochemistry for β-lactam **3** was made from ^1^H NMR coupling constants,^21^ and the observed *cis*-configuration is consistent with a steric influence of the catalyst attachments and rhodium surface on the aryl substituent of the intermediate metal carbene. The chiral Rh_2_(*S*-PTTL)_4_ catalyst was reported to exist in a crown conformation by Fox,^22^ which means all of the ligands in an “all-up” orientation, and the bulky *t*-butyl and TBS substituents of the carbene are enclosed within the crown. This crowded transition state defines conformational preference for the aryl group to approach the carbene center for C–H insertion and the Buchner reaction. The (3*S*,4*R*)-configuration of the generated chiral centers in *β*-lactam **3** was confirmed by single-crystal X-ray diffraction analysis of **3i**, and the configurations of other compounds were assigned by analogy.^23^

## Conclusions

In conclusion, we have discovered a highly selective asymmetric intramolecular C–H functionalization reaction of enoldiazoacetamides catalyzed by Rh_2_(*S*-PTTL)_4_ that occur *via* an intermediate donor–acceptor cyclopropene. The Buchner reaction was totally excluded in reactions catalysed by steric bulky dirhodium carboxylate catalyst, and β-lactam derivatives are obtained from intramolecular C–H insertion as one diastereoisomer in high yield with up to 99% enantiomeric excess. Furthermore, reactions of enoldiazoacetamides and their corresponding donor–acceptor cyclopropenes performed with copper(i) and silver(i) catalysts validate their formation of metal carbene intermediates, but they show differences in reactivity and selectivity. The high enantiocontrol that is achieved relies on both electronic and steric influences of the unique silylenol group in these metal carbene transformation.

## Supplementary Material

Supplementary information

Crystal structure data
