# Supplementary material for: Enantioselective cis-β-lactam synthesis by intramolecular C–H functionalization from enoldiazoacetamides and derivative donor–acceptor cyclopropenes
Source: Chem Sci. 2015 Jan 28;6(4):2196–false. doi: 10.1039/c4sc03991b (PMC4444070; doi:10.1039/c4sc03991b)
Supplement: Supplementary file 1 [file SC-006-C4SC03991B-s001.pdf]

Electronic Supplementary Material (ESI) for Chemical Science.
This journal is © The Royal Society of Chemistry 2015

Enantioselective cis-β-Lactam Synthesis by Intramolecular C-H Functionalization from Enoldiazoacetamides and Derivative Donor-Acceptor Cyclopropenes

Xinfang Xu,*[a] Yongming Deng,[b] David N. Yim,[b] Peter Y. Zavalij,[b] and Michael P. Doyle*[b]

[a] *Key Laboratory of Organic Synthesis of Jiangsu Province, College of Chemistry, Chemical Engineering and Materials Science, Dushu Lake Campus, Soochow University, Suzhou 215123, People's Republic of China*

[b] *Department of Chemistry and Biochemistry, University of Maryland, College Park, Maryland 20742 U.S.A.*

Supporting Information

S2 **General**

S2-6 **General Procedures for the Preparation of Enoldiazoacetates 1**

S6-15 **General Procedure for Enantioselective C-H Functionalization of 1**

S16 **Procedure for Reactions of Donor-Acceptor Cyclopropene 6 with Copper(I) and Silver(I) Catalysts**

S17 **Procedure for Acidic Hydrolysis of β-Lactams 3c**

S18 **Procedure for Enantioselective C-H Functionalization of 4a**

S18 **References**

S19 **Proton NMR Spectral Observation of Cyclopropene 6c**

S20 **Chemoselectivity Determination by ^{1}H NMR Spectrum of the Reaction Mixture**

S21-50 **NMR Spectra of 1, 2c, 3 and 5**

S51-62 **HPLC Analyses of 3 and 5**

S63 **Data Collection and Structure Refinement for (3*S*,4*R*)-3i**

General Information

All reactions were performed in oven-dried (140 °C) glassware under an atmosphere of dry N_2. DCM (dichloromethane), DCE (1,2-dichloroethane) and toluene were distilled prior to use kept over activated 3 Å molecular sieves. TBME (*tert*-butyl methyl ether), DMB (2,2-dimethylbutane) and $DCCl_3$ were purchased from Sigma Aldrich and used without further treatment. Thin layer chromatography (TLC) was carried out using EM Science silica gel 60 F254 plates. The developed chromatogram was analyzed with a UV lamp (254 nm). Liquid chromatography was performed using flash chromatography of the indicated system on silica gel (230-400 mesh). ^1H NMR and ^{13}C NMR spectra were recorded in $CDCl_3$ on a 400 MHz spectrometer; chemical shifts are reported in ppm with the solvent signals as reference, and coupling constants (J) are given in Hertz. The peak information is described as: br = broad, s = singlet, d = doublet, t = triplet, q = quartet, m = multiplet, comp = composite. Enantioselectivity was determined on an Agilent 1200 Series HPLC using Daicel Chiralcel IB-3, AD-H and OD-H columns. Optical rotation was recorded on JASCO DIP-1000 digital polarimeter. High-resolution mass spectra (HRMS) were performed on a TOF-CS mass spectrometer using CsI as the standard.

Materials

Rh_2(*S*-DOSP)$_4$ was purchased from Strem Chemicals. $Rh_2(esp)_2$ was purchased from Sigma Aldrich, and all other dirhodium carboxylates catalysts were prepared according to literature procedures.[1] The diazoacetamides **1** were prepared according to the literature procedures.[2]

General Procedure for the Preparation of Enoldiazoacetamides 1.[2]

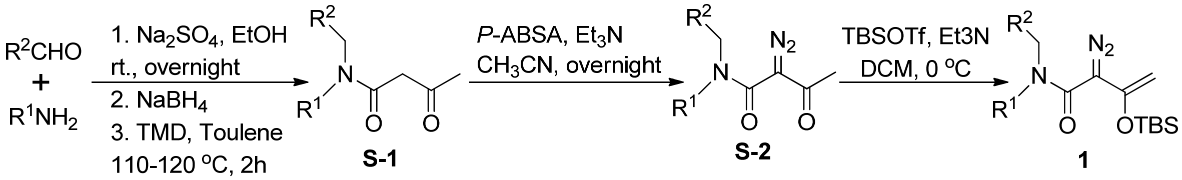

<u>Synthesis of **S-1**</u>: To a 100-mL oven-dried flask containing a magnetic stirring bar, aldehyde (10 mmol) and amine (10 mmol) in EtOH (20 mL), was added anhydrous

Na_2SO_4 (10 g). After the reaction mixture was stirred at room temperature overnight, Na_2SO_4 was filtered, and the solid was washed with MeOH (10 mL). The combined organic phase was cooled to 0 °C in an ice bath, and $NaBH_4$ (10 mmol, 378 mg) was added in portions over 30 min. The mixture was then stirred for another 2 hours at room temperature [monitored by thin layer chromatography (TLC) until all the material was consumed]. The reaction was quenched by addition of saturated aqueous $NaHCO_3$ (20 mL) with stirring for 10 min, then extracted with EtOAc (60 mL). The organic phase was washed twice with saturated aqueous NaCl (40 mL X 2) and then dried over anhydrous Na_2SO_4. After evaporating the solvents, 2,2,6-trimethyl-4*H*-1,3-dioxin-4-one (TMD, 11 mmol, 1.56 g) and toluene (40 mL) were added, and the reaction mixture was refluxed at 110-120 °C for 2 hours [monitored by thin layer chromatography (TLC) until all the material was consumed]. After evaporation of the solvents under reduced pressure, the residue was purified by column chromatography to obtain **S-1** in 50-80 % yield.

Synthesis of **S-2**: To a 100-mL oven-dried flask containing a magnetic stirring bar, **S-1** and *p*-ABSA (1.1 eq) in CH_3CN (50 mL/10 mmol) was added Et_3N (1.5 eq) slowly at 0 °C, and the resulting reaction mixture was stirred at room temperature overnight. Ether (30 mL) was then added, and the sulfonamide solid formed during the reaction was filtered through Celite. The solid was washed with ether (30 mL), and the ether washing was combined with the filtrate. The combined organic solution was washed with saturated aqueous NH_4Cl (50 mL), followed by saturated aqueous NaCl (50 mL), saturated aqueous $NaHCO_3$ (50 mL) and saturated aqueous NaCl (50 mL), then dried over anhydrous Na_2SO_4. After evaporating the solvents, the residue was purified by column chromatography and further purified by recrystallization from ether and hexanes to give pure **S-2** in 40-60% yield. (For the NMR data of compounds **S-2b**[2b], **S-2c**[2a], **S-2f** and **S-2g**[2c], see reported references)

Synthesis of **1**: To a 100-mL oven-dried flask containing a magnetic stirring bar, diazo compound **S-2** (2 mmol) and Et_3N (1.5 eq, 0.45 mL) in DCM (10 mL) was added TBSOTf (1.1 eq, 0.5 mL) slowly at 0 °C, then the reaction mixture was stirred for 0.5-1 h. Hexanes (30 mL) were added followed by saturated aqueous $NaHCO_3$ (40 mL), and the

organic phase was separated and washed two more times with saturated aqueous $NaHCO_3$ (40 mL X 2), then dried over anhydrous Na_2SO_4. After evaporating the solvents, the residue was used directly to assess its catalytic intramolecular reactions without further purification (>95% pure). Further purification by recrystallization using hexane gave the results presented below with >71% yield.

***N*-(*tert*-Butyl)-3-[(*tert*-butyldimethylsilyl)oxy]-2-diazo-*N*-(4-methoxybenzyl)but-3-enamide (1a)**: 74% yield; ^{1}H NMR (400 MHz, $CDCl_3$) δ 7.14 (d, *J* = 8.7 Hz, 2H), 6.85 (d, *J* = 8.7 Hz, 2H), 4.52 (s, 2H), 4.41 (d, *J* = 2.1 Hz, 1H), 4.16 (d, *J* = 2.1 Hz, 1H), 3.78 (s, 3H), 1.36 (s, 9H), 0.89 (s, 9H), 0.18 (s, 6H); ^{13}C NMR ($CDCl_3$, 100 MHz): δ 166.9, 158.7, 144.0, 131.2, 127.9, 113.8, 88.7, 58.0, 55.1, 50.4, 28.7, 25.5, 18.0, -4.9; HRMS (ESI) calculated for $C_{22}H_{36}N_3O_3Si$ $[M+H]^+$: 418.2520; found: 418.2523.

***N*-Benzyl-*N*-(*tert*-Butyl)-3-[(*tert*-butyldimethylsilyl)oxy]-2-diazobut-3-enamide (1b)**: 72% yield; ^{1}H NMR (400 MHz, $CDCl_3$) δ 7.38-6.91 (comp, 5H), 4.60 (s, 2H), 4.42 (d, *J* = 2.1 Hz, 1H), 4.17 (d, *J* = 2.1 Hz, 1H), 1.39 (s, 9H), 0.90 (s, 9H), 0.19 (s, 6H). ^{13}C NMR ($CDCl_3$, 100 MHz): δ 166.9, 144.0, 139.4, 128.5, 127.2, 126.7, 88.7, 58.2, 51.0, 28.8, 25.5, 18.0, -4.8; HRMS (ESI) calculated for $C_{21}H_{34}N_3O_2Si$ $[M+H]^+$: 388.2415; found: 388.2451.

***N*-(*tert*-Butyl)-3-[(*tert*-butyldimethylsilyl)oxy]-2-diazo-*N*-(4-chlorobenzyl)but-3-enamide (1c)**: 88% yield; ^{1}H NMR (400 MHz, $CDCl_3$) δ 7.22 (d, *J* = 8.4 Hz, 2H), 7.12 (d, *J* = 8.4 Hz, 2H), 4.50 (s, 2H), 4.37 (d, *J* = 2.1 Hz, 1H), 4.12 (d, *J* = 2.1 Hz, 1H), 1.32 (s, 9H), 0.84 (s, 9H), 0.13 (s, 6H). ^{13}C NMR ($CDCl_3$, 100 MHz): δ 167.1, 143.8, 138.0, 133.0, 128.7, 128.1, 89.1, 58.3, 50.3, 28.8, 25.5, 18.0, -4.8; HRMS (ESI) calculated for $C_{21}H_{33}ClN_3O_2Si$ $[M+H]^+$: 422.2025; found: 422.2062.

***N*-(*tert*-Butyl)-3-[(triisopropylsilyloxy)vinyl]-2-diazo-*N*-(4-chlorobenzyl)but-3-enamide (1c')**: 92% yield; ^{1}H NMR (400 MHz, $CDCl_3$) δ 7.30 (d, *J* = 8.5 Hz, 2H), 7.18 (d, *J* = 8.5 Hz, 2H), 4.55 (s, 2H), 4.40 (d, *J* = 2.2 Hz, 1H), 4.17 (d, *J* = 2.2 Hz, 1H), 1.39 (s, 9H), 1.25-1.18 (m, 3H), 1.07 (s, 18H); ^{13}C NMR ($CDCl_3$, 100 MHz): δ 167.5, 144.2,

138.2, 133.2, 129.0, 128.3, 88.5, 58.5, 50.5, 29.0, 18.1, 12.8; HRMS (ESI) calculated for $C_{24}H_{39}ClN_3O_2Si$ $[M+H]^+$: 464.2495; found: 464.2477.

***N*-(*tert*-Butyl)-3-[(*tert*-butyldimethylsilyl)oxy]-2-diazo-*N*-(4-methylbenzyl)but-3-enamide (1d)**: 79% yield; ^1H NMR (400 MHz, $CDCl_3$) δ 7.11 (s, 4H), 4.55 (s, 2H), 4.42 (d, *J* = 2.0 Hz, 1H), 4.16 (d, *J* = 2.0 Hz, 1H), 2.31 (s, 3H), 1.38 (s, 9H), 0.89 (s, 9H), 0.18 (s, 6H). ^{13}C NMR ($CDCl_3$, 100 MHz): δ 166.9, 144.1, 136.9, 136.3, 129.2, 126.7, 88.7, 58.2, 50.8, 28.8, 25.5, 21.0, 18.0, -4.8; HRMS (ESI) calculated for $C_{22}H_{36}N_3O_2Si$ $[M+H]^+$: 402.2571; found: 402.2578.

***N*-(*tert*-Butyl)-3-[(*tert*-butyldimethylsilyl)oxy]-2-diazo-*N*-(4-fluorobenzyl)but-3-enamide (1e)**: 87% yield; ^1H NMR (400 MHz, $CDCl_3$) δ 7.64-7.32 (comp, 4H), 4.85 (s, 2H), 4.45 (d, *J* = 1.9 Hz, 1H), 4.18 (d, *J* = 1.9 Hz, 1H), 1.42 (s, 9H), 0.84 (s, 9H), 0.13 (s, 6H). ^{13}C NMR ($CDCl_3$, 100 MHz): δ 167.1, 162.0 (d, *J* = 244.0 Hz), 143.9, 135.1 (d, *J* = 3.1 Hz), 128.3 (d, *J* = 8.1 Hz), 115.5 (d, *J* = 22.0 Hz), 89.0, 58.2, 50.2, 28.8, 25.5, 18.0, -4.8; HRMS (ESI) calculated for $C_{21}H_{33}FN_3O_2Si$ $[M+H]^+$: 406.2321; found: 406.2333.

***N*-(4-Bromobenzyl)-*N*-(*tert*-butyl)-3-[(*tert*-butyldimethylsilyl)oxy]-2-diazobut-3-enamide (1f)**: 88% yield; ^1H NMR (400 MHz, $CDCl_3$) δ 7.41 (d, *J* = 8.4 Hz, 2H), 7.08 (d, *J* = 8.4 Hz, 2H), 4.50 (s, 2H), 4.38 (d, *J* = 2.1 Hz, 1H), 4.14 (d, *J* = 2.1 Hz, 1H), 1.35 (s, 9H), 0.86 (s, 9H), 0.16 (s, 6H). ^{13}C NMR ($CDCl_3$, 100 MHz): δ 167.0, 143.8, 138.5, 131.6, 128.4, 121.0, 89.0, 58.3, 50.3, 28.8, 25.5, 18.0, -4.8; HRMS (ESI) calculated for $C_{21}H_{33}BrN_3O_2Si$ $[M+H]^+$: 466.1520; found: 466.1513.

***N*-(*tert*-Butyl)-3-[(*tert*-butyldimethylsilyl)oxy]-2-diazo-*N*-(4-nitrobenzyl)but-3-enamide (1g)**: 90% yield; ^1H NMR (400 MHz, $CDCl_3$) δ 8.11 (d, *J* = 8.7 Hz, 2H), 7.37 (d, *J* = 8.7 Hz, 2H), 4.63 (s, 2H), 4.36 (d, *J* = 2.1 Hz, 1H), 4.11 (d, *J* = 2.1 Hz, 1H), 1.35 (s, 9H), 0.80 (s, 9H), 0.10 (s, 6H). ^{13}C NMR ($CDCl_3$, 100 MHz): δ 167.1, 147.2, 147.1, 143.5, 127.3, 123.8, 89.5, 58.5, 50.1, 28.8, 25.4, 18.0, -4.9; HRMS (ESI) calculated for $C_{21}H_{33}N_4O_4Si$ $[M+H]^+$: 433.2266; found: 433.2255.

***N*-[(1,1'-Biphenyl)-4-ylmethyl]-*N*-(*tert*-butyl)-3-[(tert-butyldimethylsilyl)oxy]-2-diazobut-3-enamide (1h)**: 80% yield; ^{1}H NMR (400 MHz, $CDCl_3$) δ 7.58-7.26 (comp, 9H), 4.63 (s, 2H), 4.47 (d, J = 2.1 Hz, 1H), 4.21 (d, J = 2.1 Hz, 1H), 1.47 (s, 9H), 0.95 (s, 9H), 0.12 (s, 6H). ^{13}C NMR ($CDCl_3$, 100 MHz): δ 167.0, 144.0, 140.5, 140.1, 138.5, 128.7, 127.3, 127.2, 127.2, 126.9, 88.8, 58.3, 50.7, 28.8, 25.5, 18.0, -4.8; HRMS (ESI) calculated for $C_{27}H_{38}N_3O_2Si$ $[M+H]^+$: 464.2728; found: 464.2700.

***N*-(*tert*-Butyl)-3-[(*tert*-butyldimethylsilyl)oxy]-2-diazo-*N*-[4-(dimethylamino)benzyl]-but-3-enamide (1i)**: 71% yield; ^{1}H NMR (400 MHz, $CDCl_3$) δ 7.08 (d, J = 8.7 Hz, 2H), 6.68 (d, J = 8.7 Hz, 2H), 4.51 (s, 2H), 4.43 (d, J = 2.0 Hz, 1H), 4.16 (d, J = 2.0 Hz, 1H), 2.92 (s, 6H), 1.37 (s, 9H), 0.90 (s, 9H), 0.20 (s, 6H). ^{13}C NMR ($CDCl_3$, 100 MHz): δ 166.8, 149.8, 144.2, 127.8, 126.7, 112.5, 88.5, 58.0, 50.7, 40.5, 28.8, 25.6, 18.0, -4.8; HRMS (ESI) calculated for $C_{23}H_{39}N_4O_2Si$ $[M+H]^+$: 431.2837; found: 431.2825.

General Procedure for Enantioselective C-H Functionalization of 1.

To an 10-mL oven-dried vial containing a magnetic stirring bar, Rh_2(*S*-PTTL)$_4$ (2.0 mol%, 5.0 mg) in DMB (0.5 mL), was added enoldiazoacetamide **1** (0.20 mmol) in DMB (0.5 mL) over 10 min via a syringe pump at 0 °C. The reaction mixture was stirred for the indicated time under this condition. After the reaction was complete, the reaction mixture was directly purified by column chromatography on silica gel without any additional treatment (eluent: hexanes:EtOAc = 100:0 to 90:10) to give pure β-lactams **3** in >80% yield with high to excellent enantioselectivity.

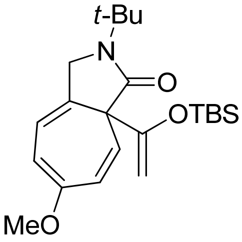

2-*tert*-Butyl-8a-[1-(*tert*-butyldimethylsilyloxy)vinyl]-6-methoxyl-2,3-dihydrocyclohepta[c]pyrrol-1(8a*H*)-one (2a). ^{1}H NMR ($CDCl_3$, 400 MHz) δ 6.13 (dd, J = 10.7, 2.0 Hz, 1H), 6.11-6.04 (m, 1H), 5.69 (d, J = 10.7 Hz, 1H), 5.64-5.56 (m,

1H), 4.26 (dd, J = 14.2, 1.4 Hz, 1H), 4.21 (d, J = 1.9 Hz, 1H), 4.11 (dd, J = 14.2, 1.4 Hz, 1H), 4.08 (d, J = 1.9 Hz, 1H), 3.57 (s, 3H), 1.42 (s, 9H), 0.87 (s, 9H), 0.09 (s, 3H), 0.04 (s, 3H).

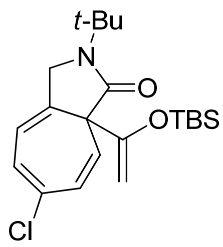

2-*tert*-Butyl-8a-[1-(*tert*-butyldimethylsilyloxy)vinyl]-6-chloro-2,3-dihydrocyclohepta[c]pyrrol-1(8a*H*)-one (2c). White solid, mp = 79.8 °C - 84.1 ℃. ^{1}H NMR ($CDCl_3$, 400 MHz) δ 6.57 (d, J = 6.8 Hz, 1H), 6.29 (dd, J = 10.4 Hz, J = 1.2 Hz, 1H), 6.12 (dt, J = 6.8 Hz, 2.0 Hz, 1H), 5.59 (d, J = 10.4 Hz, 1H), 4.29 (d, J = 15.2 Hz, 1H), 4.24 (d, J = 2.0 Hz, 1H), 4.17 (d, J = 15.2 Hz, 1H), 4.13 (d, J = 2.0 Hz, 1H), 1.44 (s, 9H), 0.87 (s, 9H), 0.11 (s, 3H), 0.04 (s, 3H); ^{13}C NMR ($CDCl_3$, 100 MHz) δ 173.3, 152.7, 134.4, 134.1, 128.8, 127.6, 126.2, 117.6, 91.2, 60.3, 54.5, 49.8, 27.6, 25.7, 18.1, -4.4, -5.3; HRMS (ESI) calculated for $C_{21}H_{33}ClNO_2Si$ $[M+H]^+$: 394.1964; found: 394.1966.

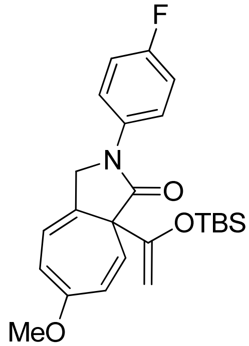

8a-[1-(*tert*-butyldimethylsilyloxy)vinyl]-2-(4-fluorophenyl)-6-methoxy-2,3-dihydrocyclohepta[c]pyrrol-1(8a*H*)-one (2o). ^{1}H NMR ($CDCl_3$, 400 MHz) δ 7.70-7.59 (m, 2H), 7.08 (dd, J = 9.2, 8.4 Hz, 2H), 6.39 (d, J = 8.4 Hz, 2H), 6.23 (d, J = 8.4 Hz, 2H), 4.07 (s, 2H), 3.44 (s, 3H), 2.39 (s, 2H), 0.95 (s, 9H), 0.22 (s, 6H); ^{13}C NMR ($CDCl_3$, 100 MHz) δ 164.6, 159.6 (d, J = 243.7 Hz), 156.3, 139.8, 136.33 (d, J = 2.8 Hz), 133.5, 121.78 (d, J = 7.8 Hz), 118.3, 115.83 (d, J = 22.3 Hz), 57.6, 52.5, 40.5, 26.3, 18.9, -3.6; HRMS (ESI) calculated for $C_{24}H_{31}FNO_3Si$ $[M+H]^+$: 428.2052; found: 428.2019.

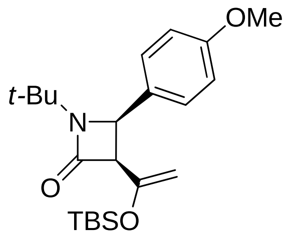

(3*S*,4*R*)-1-(*tert*-Butyl)-3-[1-(*tert*-butyldimethylsilyloxy)vinyl]-4-(4-methoxyphenyl)azetidin-2-one (3a). 85% yield; 92% *ee*; $[\alpha]_D^{20}$ = +41.8° (c = 1, EtOAc); HPLC conditions for determination of enantiomeric excess: IB-3 column, 210 nm, 0.5 mL/min, hexanes:IPA:EtOH = 98.5:1:0.5, t_r = 10.5, 11.3 min. ^{1}H NMR (400 MHz, $CDCl_3$): δ (ppm) 7.27 (d, *J* = 8.7 Hz, 2H), 6.82 (d, *J* = 8.7 Hz, 2H), 4.75 (d, *J* = 5.8 Hz, 1H), 4.48 (s, 1H), 4.06 (d, *J* = 1.4 Hz, 1H), 3.95 (d, *J* = 5.8 Hz, 1H), 3.79 (s, 3H), 1.26 (s, 9H), 0.72 (s, 9H), -0.03 (s, 3H), -0.32 (s, 3H); ^{13}C NMR ($CDCl_3$, 100 MHz): 166.9, 159.6, 151.2, 129.7, 129.1, 113.4, 93.1, 59.9, 57.7, 55.5, 54.5, 28.3, 25.7, 17.9, -4.9, -5.4; HRMS (ESI) calculated for $C_{22}H_{36}NO_3Si$ $[M+H]^+$: 390.2459; found: 390.2455.

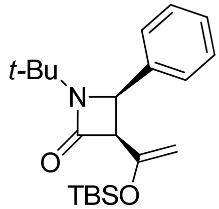

(3*S*,4*R*)-1-(*tert*-Butyl)-3-[1-(*tert*-butyldimethylsilyloxy)vinyl]-4-phenylazetidin-2-one (3b). Catalyzed by Rh_2(*S*-NTTL)$_4$ instead of Rh_2(*S*-PTTL)$_4$: 81% yield; 87% *ee*; HPLC conditions for determination of enantiomeric excess: AD-H column, 210 nm, 0.4 mL/min, hexanes:IPA = 99:1, t_r = 13.1, 14.7 min. ^{1}H NMR (400 MHz, $CDCl_3$): δ (ppm) 7.37-7.30 (m, 2H), 7.26-7.24 (m, 3H), 4.78 (d, *J* = 5.8 Hz, 1H), 4.51-4.45 (s, 1H), 4.03 (d, *J* = 1.6 Hz, 1H), 3.98 (d, *J* = 5.8 Hz, 1H), 1.26 (s, 9H), 0.70 (s, 9H), -0.06 (s, 3H), -0.38 (s, 3H); ^{13}C NMR ($CDCl_3$, 100 MHz): 166.8, 150.9, 137.5, 128.0, 127.8, 93.1, 59.9, 58.1, 54.5, 28.3, 25.6, 17.8, -5.0, -5.6; HRMS (ESI) calculated for $C_{21}H_{34}NO_2Si$ $[M+H]^+$: 360.2353; found: 360.2365.

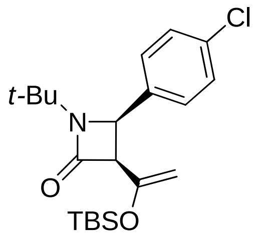

(3*S*,4*R*)-1-(*tert*-Butyl)-3-[1-(*tert*-butyldimethylsilyloxy)vinyl]-4-(4-chlorophenyl)azetidin-2-one (3c). 88% yield; 93% *ee*; $[\alpha]_D^{20}$ = +67.8° (c = 1, EtOAc); HPLC conditions for determination of enantiomeric excess: AD-H column, 210 nm, 0.4 mL/min, hexanes:IPA = 98:2, t_r = 11.2, 12.4 min. ^{1}H NMR (400 MHz, $CDCl_3$): δ (ppm) 7.31-7.25 (m, 4H), 4.76 (d, *J* = 5.8 Hz, 1H), 4.52-4.45 (m, 1H), 4.06 (d, *J* = 1.6 Hz, 1H), 3.98 (d, *J* = 5.8 Hz, 1H), 1.26 (s, 9H), 0.72 (s, 9H), -0.02 (s, 3H), -0.30 (s, 3H); ^{13}C NMR ($CDCl_3$, 100 MHz): 166.7, 150.7, 136.4, 133.9, 129.3, 128.1, 93.6, 60.0, 57.4, 54.8, 28.4, 25.7, 18.0, -4.9, -5.4; HRMS (ESI) calculated for $C_{21}H_{33}ClNO_2Si$ $[M+H]^+$: 394.1964; found: 394.1981.

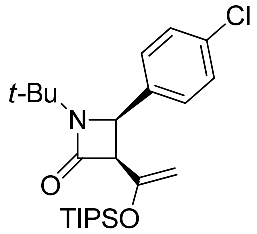

(3*S*,4*R*)-1-(*tert*-Butyl)-3-[1-(triisopropylsilyloxy)vinyl]-4-(4-chlorophenyl)azetidin-2-one (3c'). 84% yield; 92% *ee*; HPLC conditions for determination of enantiomeric excess: IC column, 190 nm, 0.5 mL/min, hexanes:IPA = 98:2, t_r = 11.1, 11.6 min. ^{1}H NMR (400 MHz, $CDCl_3$): δ (ppm) 7.71-7.01 (m, 4H), 4.78 (d, *J* = 5.9 Hz, 1H), 4.51 (d, *J* = 1.3 Hz, 1H), 4.11 (d, *J* = 1.3 Hz, 1H), 4.03 (d, *J* = 5.9 Hz, 1H), 1.28 (s, 9H), 1.01-0.92 (m, 3H), 0.90-0.84 (m, 18H); ^{13}C NMR ($CDCl_3$, 100 MHz): 166.8, 150.8, 136.5, 134.0, 129.3, 128.2, 93.8, 60.1, 57.5, 54.8, 18.1, 17.9, 12.5; HRMS (ESI) calculated for $C_{24}H_{39}ClNO_2Si$ $[M+H]^+$: 436.2433; found: 436.2410.

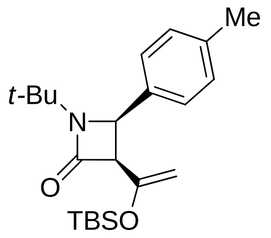

(3*S*,4*R*)-1-(*tert*-Butyl)-3-[1-(*tert*-butyldimethylsilyloxy)vinyl]-4-(4-methylphenyl)azetidin-2-one (3d). 92% yield; 93% *ee*; $[\alpha]_D^{20}$ = +67.9° (c = 1, EtOAc); HPLC conditions for determination of enantiomeric excess: AD-H column, 210 nm, 0.5 mL/min, hexanes:IPA:EtOH = 98:1:1, t_r = 10.8, 11.9 min. ^{1}H NMR (400 MHz, $CDCl_3$): δ (ppm) 7.20 (d, *J* = 8.0 Hz, 2H), 7.04 (d, *J* = 8.0 Hz, 2H), 4.73 (d, *J* = 5.8 Hz, 1H), 4.44-4.43 (m, 1H), 4.01 (d, *J* = 1.4 Hz, 1H), 3.93 (d, *J* = 5.8 Hz, 1H), 2.28 (s, 3H), 1.24 (s, 9H), 0.68 (s, 9H), -0.08 (s, 3H), -0.38 (s, 3H); ^{13}C NMR ($CDCl_3$, 100 MHz): 166.8, 151.0, 137.6, 134.5, 128.5, 127.9, 93.0, 59.9, 57.9, 54.5, 28.3, 25.6, 21.2, 17.9, -5.0, -5.7; HRMS (ESI) calculated for $C_{22}H_{36}NO_2Si$ $[M+H]^+$: 374.2510; found: 374.2540.

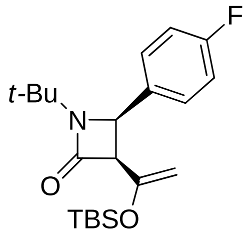

(3*S*,4*R*)-1-(*tert*-Butyl)-3-[1-(*tert*-butyldimethylsilyloxy)vinyl]-4-(4-fluorophenyl)azetidin-2-one (3e). 88% yield; 91% *ee*; $[\alpha]_D^{20}$ = +68.0° (c = 1, EtOAc); HPLC conditions for determination of enantiomeric excess: AD-H column, 210 nm, 0.5 mL/min, hexanes:IPA:EtOH = 99:0.5:0.5, t_r = 9.7, 11.8 min. ^{1}H NMR (400 MHz, $CDCl_3$): δ (ppm) 7.36-7.29 (m, 2H), 6.99 (t, *J* = 8.7 Hz, 2H), 4.79 (d, *J* = 5.8 Hz, 1H), 4.52-4.47 (m, 1H), 4.07 (d, *J* = 1.6 Hz, 1H), 3.99 (d, *J* = 5.8 Hz, 1H), 1.27 (s, 9H), 0.73 (s, 9H), -0.01 (s, 3H), -0.29 (s, 3H); ^{13}C NMR ($CDCl_3$, 100 MHz): 166.8, 162.8 (d, *J* = 246.0 Hz), 150.8, 133.5 (d, *J* = 3.1 Hz), 129.6 (d, *J* = 8.1 Hz), 114.8 (d, *J* = 21.6 Hz), 93.5, 60.0, 57.4, 54.7, 28.4, 25.6, 17.9, -5.0, -5.4; HRMS (ESI) calculated for $C_{21}H_{33}FNO_2Si$ $[M+H]^+$: 378.2259; found: 378.2243.

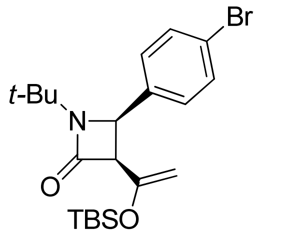

(3*S*,4*R*)-4-(4-Bromophenyl)-1-(*tert*-butyl)-3-[1-(*tert*-butyldimethylsilyloxy)vinyl]azetidin-2-one (3f). 89% yield; 89% *ee*; HPLC conditions for determination of enantiomeric excess: AD-H column, 210 nm, 0.5 mL/min, hexanes:IPA:EtOH = 99:0.5:0.5, t_r = 10.3, 12.4 min. ^{1}H NMR (400 MHz, $CDCl_3$): δ (ppm) 7.43 (d, *J* = 8.5 Hz, 2H), 7.25 (d, *J* = 8.5 Hz, 2H), 4.76 (d, *J* = 5.8 Hz, 1H), 4.52-4.45 (m, 1H), 4.08 (d, *J* = 1.6 Hz, 1H), 3.99 (d, *J* = 5.8 Hz, 1H), 1.28 (s, 9H), 0.73 (s, 9H), -0.01 (s, 3H), -0.28 (s, 3H); ^{13}C NMR ($CDCl_3$, 100 MHz): 166.7, 150.7, 136.9, 131.0, 129.7, 122.0, 93.6, 60.0, 57.4, 54.8, 28.4, 25.7, 18.0, -4.9, -5.4; HRMS (ESI) calculated for $C_{21}H_{33}BrNO_2Si$ $[M+H]^+$: 438.1458; found: 438.1443.

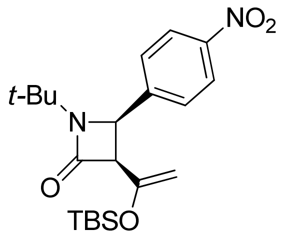

(3*S*,4*R*)-1-(*tert*-Butyl)-3-[1-(*tert*-butyldimethylsilyloxy)vinyl]-4-(4-nitrophenyl)azetidin-2-one (3g). 88% yield; 83% *ee*; HPLC conditions for determination of enantiomeric excess: AD-H column, 210 nm, 0.5 mL/min, hexanes:IPA:EtOH = 98:0.5:1.5, t_r = 11.5, 12.7 min. ^{1}H NMR (400 MHz, $CDCl_3$): δ (ppm) 8.15 (d, *J* = 8.8 Hz, 2H), 7.54 (d, *J* = 8.8 Hz, 2H), 4.90 (d, *J* = 6.0 Hz, 1H), 4.52-4.45 (m, 1H), 4.07-4.01 (comp, 2H), 1.26 (s, 9H), 0.69 (s, 9H), -0.05 (s, 3H), -0.34 (s, 3H).; ^{13}C NMR ($CDCl_3$, 100 MHz): 166.3, 150.2, 147.8, 145.6, 128.7, 123.0, 94.2, 60.3, 57.0, 54.9, 28.3, 25.5, 17.8, -5.1, -5.4; HRMS (ESI) calculated for $C_{21}H_{33}N_2O_4Si$ $[M+H]^+$: 405.2204; found: 405.2235.

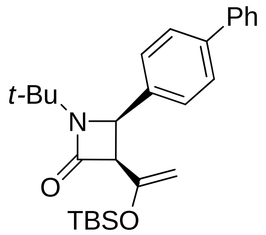

(3*S*,4*R*)-1-(*tert*-Butyl)-3-[1-(*tert*-butyldimethylsilyloxy)vinyl]-4-(4-phenylphenyl)azetidin-2-one (3h). 89% yield; 91% *ee*; $[\alpha]_D^{20}$ = +49.5° (c = 1, EtOAc); HPLC conditions for determination of enantiomeric excess: AD-H column, 210 nm, 0.5 mL/min, hexanes:IPA:EtOH = 98:1:1, t_r = 12.8, 14.9 min. ^{1}H NMR (400 MHz, $CDCl_3$): δ (ppm) 7.59-7.36 (comp, 9H), 4.84 (d, *J* = 5.8 Hz, 1H), 4.56-4.50 (m, 1H), 4.08 (d, *J* = 1.6 Hz, 1H), 4.03 (d, *J* = 5.8 Hz, 1H), 1.32 (s, 9H), 0.72 (s, 9H), -0.03 (s, 3H), -0.35 (s, 3H). ^{13}C NMR ($CDCl_3$, 100 MHz): 166.9, 151.0, 141.2, 141.1, 136.8, 129.0, 128.5, 127.4, 127.2, 126.7, 93.2, 60.1, 57.9, 54.7, 28.4, 25.7, 18.0, -4.9, -5.5; HRMS (ESI) calculated for $C_{27}H_{38}NO_2Si$ $[M+H]^+$: 436.2666; found: 436.2690.

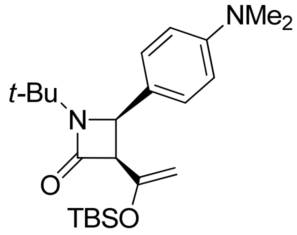

(3*S*,4*R*)-1-(*tert*-Butyl)-3-[1-(*tert*-butyldimethylsilyloxy)vinyl]-4-[4-(dimethylamino)phenyl]azetidin-2-one (3i). 80% yield; 99% *ee*; $[\alpha]_D^{20}$ = +54.1° (c = 1, EtOAc); HPLC conditions for determination of enantiomeric excess: OD-H column, 254 nm, 0.7 mL/min, hexanes:IPA = 98:2, t_r = 7.3, 10.1 min. ^{1}H NMR (400 MHz, $CDCl_3$): δ (ppm) 7.21 (d, *J* = 8.8 Hz, 2H), 6.66 (d, *J* = 8.8 Hz, 2H), 4.72 (d, *J* = 5.7 Hz, 1H), 4.51-4.47 (m, 1H), 4.07 (d, *J* = 1.4 Hz, 1H), 3.94 (d, *J* = 5.7 Hz, 1H), 2.93 (s, 6H), 1.29 (s, 9H), 0.74 (s, 9H), -0.01 (s, 3H), -0.30 (s, 3H).; ^{13}C NMR ($CDCl_3$, 100 MHz): 167.1, 151.5, 150.8, 128.8, 125.3, 112.3, 93.0, 59.9, 58.1, 54.4, 41.0, 28.4, 25.8, 18.0, -4.8, -5.4; HRMS (ESI) calculated for $C_{23}H_{39}N_2O_2Si$ $[M+H]^+$: 403.2775; found: 403.2789.

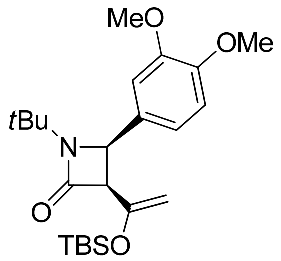

(3*S*,4*R*)-1-(*tert*-Butyl)-3-[1-(*tert*-butyldimethylsilyloxy)vinyl]-4-(3,4-dimethoxyphenyl)azetidin-2-one (3j). 81% yield; 77% *ee*; HPLC conditions for determination of enantiomeric excess: OD-H column, 230 nm, 0.7 mL/min, hexanes:IPA = 98:2, t_r = 9.6, 12.8 min. ^{1}H NMR (400 MHz, $CDCl_3$): δ (ppm) 6.93-6.86 (comp, 2H), 6.78 (d, *J* = 8.2 Hz, 1H), 4.74 (d, *J* = 5.6 Hz, 1H), 4.54 (d, *J* = 1.3 Hz, 1H), 4.10 (d, *J* = 1.3 Hz, 1H), 3.96 (d, *J* = 5.6 Hz, 1H), 3.86 (s, 3H), 3.84 (s, 3H), 1.28 (s, 9H), 0.70 (s, 9H), -0.01 (s, 3H), -0.31 (s, 3H); ^{13}C NMR ($CDCl_3$, 100 MHz): 166.8, 151.5, 149.0, 148.5, 130.2, 120.7, 111.0, 110.8, 93.1, 59.8, 58.0, 56.2, 55.8, 54.6, 28.3, 25.6, 17.9, -5.0, -5.4; HRMS (ESI) calculated for $C_{23}H_{38}NO_4Si$ $[M+H]^+$: 420.2565; found: 420.2553.

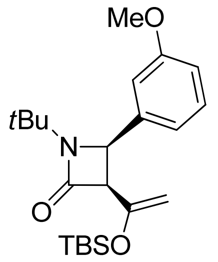

(3*S*,4*R*)-1-(*tert*-Butyl)-3-[1-(*tert*-butyldimethylsilyloxy)vinyl]-4-(3-methoxyphenyl)azetidin-2-one (3k). 92% yield; 77% *ee*; HPLC conditions for determination of enantiomeric excess: AD-H column, 230 nm, 0.5 mL/min, hexanes:IPA:Et = 98:1:1, t_r = 11.7, 12.9 min. ^{1}H NMR (400 MHz, $CDCl_3$): δ (ppm) 7.18 (t, *J* = 7.9 Hz, 1H), 6.93-6.91 (m, 2H), 6.82-6.79 (m, 1H), 4.75 (d, *J* = 5.8 Hz, 1H), 4.50 (d, *J* = 1.5 Hz, 1H), 4.07 (d, *J* = 1.5 Hz, 1H), 3.98 (d, *J* = 5.8 Hz, 1H), 3.78 (s, 3H), 1.28 (s, 9H), 0.71 (s, 9H), -0.03 (s, 3H), -0.33 (s, 3H); ^{13}C NMR ($CDCl_3$, 100 MHz): 166.9, 159.3, 151.1, 139.3, 129.0, 120.7, 113.7, 113.5, 93.2, 59.9, 58.2, 55.3, 54.7, 28.3, 25.7, 17.9, -5.0, -5.5; HRMS (ESI) calculated for $C_{22}H_{36}NO_3Si$ $[M+H]^+$: 390.2459; found: 390.2470.

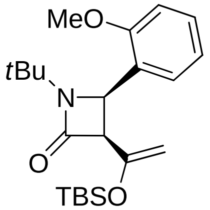

(3*S*,4*R*)-1-(*tert*-Butyl)-3-[1-(*tert*-butyldimethylsilyloxy)vinyl]-4-(2-methoxyphenyl)azetidin-2-one (3l). 85% yield; 25% *ee*; HPLC conditions for determination of enantiomeric excess: AD-H column, 230 nm, 0.5 mL/min, hexanes:IPA:Et = 98:1:1, t_r = 10.2, 11.6 min. ^{1}H NMR (400 MHz, $CDCl_3$): δ (ppm) 7.41 (d, *J* = 7.4 Hz, 1H), 7.22-7.14 (m, 1H), 6.88 (t, *J* = 7.4 Hz, 1H), 6.78 (d, *J* = 7.7 Hz, 1H), 5.35 (d, *J* = 5.6 Hz, 1H), 4.41 (d, *J* = 1.4 Hz, 1H), 4.01 (d, *J* = 1.4 Hz, 1H), 3.97 (d, *J* = 5.6 Hz, 1H), 3.78 (s, 3H), 1.26 (s, 9H), 0.71 (s, 9H), -0.07 (s, 3H), -0.32 (s, 3H); ^{13}C NMR ($CDCl_3$, 100 MHz): 157.2, 151.4, 128.7, 128.6, 125.5, 119.7, 109.8, 93.2, 59.4, 55.2, 54.3, 50.3, 28.2, 25.5, 17.9, -5.0, -5.4; HRMS (ESI) calculated for $C_{22}H_{36}NO_3Si$ $[M+H]^+$: 390.2459; found: 390.2471.

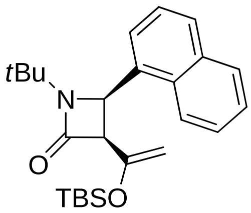

(3*S*,4*R*)-1-(*tert*-Butyl)-3-[1-(*tert*-butyldimethylsilyloxy)vinyl]-4-(1-naphthyl)azetidin-2-one (3m). 85% yield; 24% *ee*; HPLC conditions for determination of enantiomeric excess: AD-H column, 230 nm, 0.5 mL/min, hexanes:IPA:Et = 98:1:1, t_r = 10.2, 12.9 min. ^{1}H NMR (400 MHz, $CDCl_3$): δ (ppm) 8.10 (d, *J* = 8.2 Hz, 1H), 7.88-7.81 (m, 1H), 7.76 (d, *J* = 8.2 Hz, 1H), 7.73-7.66 (m, 1H), 7.52-7.41 (comp, 3H), 5.73 (d, *J* = 5.8 Hz, 1H), 4.36 (d, *J* = 1.4 Hz, 1H), 4.18 (d, *J* = 5.8 Hz, 1H), 3.94 (d, *J* = 1.4 Hz, 1H), 1.33 (s, 9H), 0.47 (s, 9H), -0.23 (s, 3H), -0.64 (s, 3H); ^{13}C NMR ($CDCl_3$, 100 MHz): 167.6, 151.0, 133.6, 132.6, 131.5, 129.0, 128.1, 126.3, 125.48, 152.46, 124.7, 122.5, 93.6, 60.5, 54.6, 53.5, 28.2, 25.4, 17.6, -5.1, -5.8; HRMS (ESI) calculated for $C_{25}H_{36}NO_2Si$ $[M+H]^+$: 410.2510; found: 410.2535.

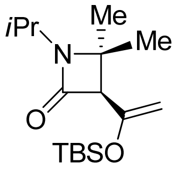

3-[1-(*tert*-Butyldimethylsilyloxy)vinyl]-1-isopropyl-4,4-dimethylazetidin-2-one (3n). 81% yield; 67% *ee*; HPLC conditions for determination of enantiomeric excess: AD-H column, 230 nm, 0.5 mL/min, hexanes:IPA = 98:2, t_r = 10.3, 12.4 min. ^{1}H NMR (400 MHz, $CDCl_3$): δ (ppm) 4.40-4.35 (m, 1H), 4.23 (d, *J* = 1.4 Hz, 1H), 3.61-3.54 (m, 1H), 3.35 (d, *J* = 0.5 Hz, 1H), 1.43 (s, 3H), 1.37 (s, 3H), 1.31 (dd, *J* = 6.8, 1.9 Hz, 6H), 0.92 (s, 9H), 0.20 (s, 3H), 0.19 (s, 3H).; ^{13}C NMR ($CDCl_3$, 100 MHz): 164.8, 152.6, 93.0, 65.3, 60.2, 44.2, 27.4, 25.8, 22.0, 21.9, 21.7, 18.0, -4.6, -4.7; HRMS (ESI) calculated for $C_{16}H_{32}NO_2Si$ $[M+H]^+$: 298.2197; found: 298.2162.

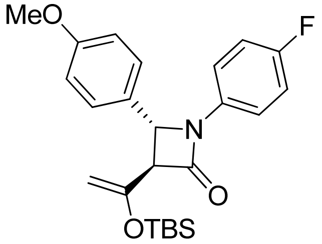

(3*S*,4*R*)-3-[1-(*tert*-butyldimethylsilyloxy)vinyl]-1-(4-fluorophenyl)-4-(4-methoxyphenyl)azetidin-2-one (3o). ^{1}H NMR (400 MHz, $CDCl_3$): δ (ppm) 7.39-7.29 (m, 2H), 7.23 (d, *J* = 8.7 Hz, 2H), 6.95 (t, *J* = 8.7 Hz, 2H), 6.85 (d, *J* = 8.7 Hz, 2H), 5.17 (d, *J* = 6.0 Hz, 1H), 4.46 (dd, *J* = 1.8, 0.8 Hz, 1H), 4.28 (d, *J* = 6.0 Hz, 1H), 4.10 (d, *J* = 1.8 Hz, 1H), 3.80 (s, 3H), 0.80 (s, 9H), -0.00 (s, 3H), -0.21 (s, 3H); ^{13}C NMR ($CDCl_3$, 100 MHz): 164.1, 159.9, 159.2 (d, *J* = 242.0 Hz), 150.5, 134.1 (d, *J* = 2.7 Hz), 129.0, 126.2, 118.8 (d, *J* = 7.8 Hz), 116.0 (d, *J* = 22.6 Hz), 113.9, 93.8, 61.4, 58.9, 55.5, 25.1, 18.1, -4.9, -5.3; HRMS (ESI) calculated for $C_{24}H_{31}FNO_3Si$ $[M+H]^+$: 428.2052; found: 428.2021.

Procedure for Reactions of Donor-Acceptor Cyclopropene 6 with Copper(I) and Silver(I) Catalysts

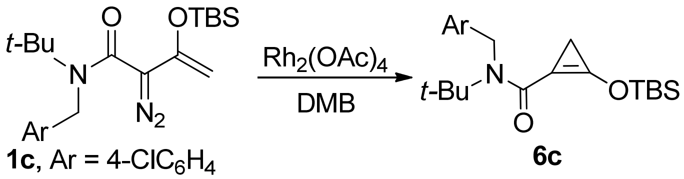

To an 10-mL oven-dried vial containing a magnetic stirring bar, $Rh_2(OAc)_4$ (1.8 mg 2.0 mol%) in DMB (1.0 mL), was added enoldiazoacetates **1c** (84 mg, 0.20 mmol) in DMB (0.5 mL) at room temperature. The reaction mixture was stirred for another 5 min, during which time the diazo compound was converted to the corresponding cyclopropene. Then the reaction mixture was passed through Celite to remove $Rh_2(OAc)_4$. After removing DMB by vacuum evaporation, the resulting cyclopropene intermediate **6c** was used for subsequent reactions.

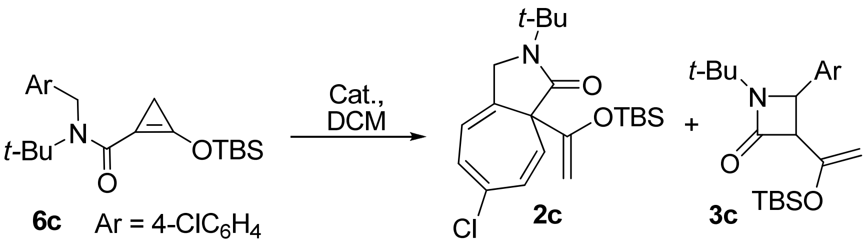

In a two dram vial equipped with N_2, $Cu(MeCN)_4PF_6$ (0.02 mol) or $AgSbF_6$ (0.02 mol) and 2,2′-isopropylidenebis[(4*S*)-4-*tert*-butyl-2-oxazoline] (0.024 mol) in DCM (0.5 mL) were stirred for 1 h under room temperature. The temperature of the solution was then cooled down to 0 °C. The cyclopropene intermediate **6c** in DCM (0.5 mL) was added dropwise to the solution under N_2 protection. The reaction mixture was stirred for another 12 h at 0 °C and then passed through Celite to remove catalyst. After removing solvent, the ratio of **2c** to **3c** was determined by ^1H NMR analysis of the corresponding reaction mixture. The resulting reaction mixture was then purified by column chromatography on silica gel (eluent: hexanes:EtOAc = 100:0 to 90:10) to give pure β-lactams **3c** and compound **2c**.

Procedure for Acidic Hydrolysis of β-Lactams 3c.

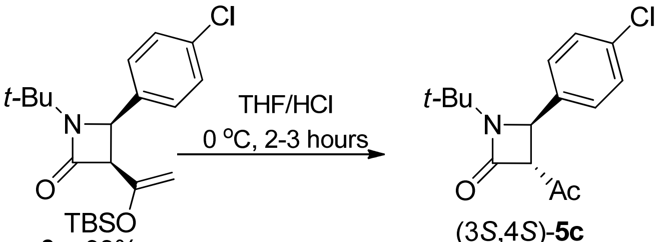

3c, 93% ee

(3*S*,4*S*)-**5c**
77% yield, 93% *ee*

To a 10-mL oven-dried vial containing a magnetic stirring bar, β-lactams **3c** (59 mg, 1.5 mol) in THF (1.5 mL), was added HCl (2 M, 0.5 mL) at 0 °C. The reaction mixture was stirred 2-3 h under this temperature. After the reaction was finished [monitored by thin layer chromatography (TLC) until all of the material was consumed], the reaction mixture was neutralized with saturated aqueous $NaHCO_3$, extracted twice with ether (10 mL X 2), and the combined organic phase was dried over anhydrous Na_2SO_4. After evaporating the solvents, the residue was purified by column chromatography on silica gel (eluent: hexanes:EtOAc = 90:10 to 80:20) to give pure β-lactam **5c** in 77% yield (32 mg) with 93% *ee* and >20:1 *dr*.

(3*R*,4*R*)-3-Acetyl-1-(*tert*-butyl)-4-(4-chlorophenyl)azetidin-2-one (5c). 77% yield; 93% *ee*; HPLC conditions for determination of enantiomeric excess: AD-H column, 230 nm, 0.7 mL/min, hexanes:IPA = 97:3, t_r = 14.7, 19.0 min. ^{1}H NMR (400 MHz, $CDCl_3$): δ (ppm) 7.34 (s, 4H), 5.00 (d, *J* = 2.2 Hz, 1H), 3.87 (d, *J* = 2.2 Hz, 1H), 2.28 (s, 3H), 1.23 (s, 9H); ^{13}C NMR ($CDCl_3$, 100 MHz): 199.6, 163.0, 138.2, 134.5, 129.3, 128.1, 71.0, 55.4, 53.9, 30.2, 28.3; HRMS (ESI) calculated for $C_{15}H_{19}ClNO_2$ $[M+H]^+$: 280.1099; found: 280.1090.

General Procedure for Enantioselective C-H Functionalization of 4a.

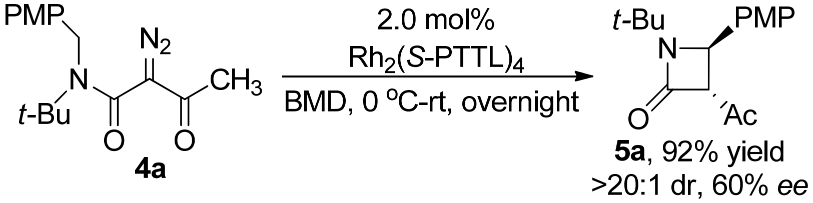

To a 10-mL oven-dried vial containing a magnetic stirring bar, Rh_2(*S*-PTTL)$_4$ (2.0 mol%, 5.0 mg) in DMB (0.5 mL), was added diazoacetamide **4a** (61 mg, 0.20 mmol) in DMB (0.5 mL) over 10 min via a syringe pump at 0 °C. The reaction mixture was stirred overnight and warmed to room temperature slowly. After the reaction was complete [monitored by thin layer chromatography (TLC) until all of the material was consumed], the reaction mixture was directly purified by column chromatography on silica gel without any further treatment (eluent: hexanes:EtOAc = 90:0 to 80:10) to give pure β-lactam **5a** in 92% yield (51 mg) with 60% *ee* and >20:1 *dr*.

(3*R*,4*R*)-3-Acetyl-1-(*tert*-butyl)-4-(4-methoxyphenyl)azetidin-2-one (5a). 92% yield; 60% *ee*; HPLC conditions for determination of enantiomeric excess: AD-H column, 210 nm, 1.0 mL/min, hexanes:IPA = 95:5, t_r = 13.2, 17.1 min. ^{1}H NMR (400 MHz, $CDCl_3$): δ (ppm) 7.34 (d, *J* = 8.7 Hz, 2H), 6.92 (d, *J* = 8.7 Hz, 2H), 4.99 (d, *J* = 2.2 Hz, 1H), 3.92 (d, *J* = 2.2 Hz, 1H), 3.84 (s, 3H), 2.31 (s, 3H), 1.26 (s, 9H); ^{13}C NMR ($CDCl_3$, 100 MHz): 200.2, 163.3, 160.0, 131.3, 128.1, 114.5, 71.1, 55.5, 55.3, 54.3, 30.3, 28.3; HRMS (ESI) calculated for $C_{16}H_{22}NO_3$ $[M+H]^+$: 276.1594; found: 276.1584.

References

1. (a) S. Hashimoto, N. Watanabe, T. Sato, M. Shiro and S. Ikegami, *Tetrahedron lett.*, 1993, **34**, 5109; (b) H. Tsutsui, T. Abe, S. Nakamura, M. Anada and S. Hashimoto, *Chem. Pharm. Bull.*, 2005, **53**, 1366; (c) S. Kitagaki, M. Anada, O. Kataoka, K. Matsuno, C. Umeda, N. Watanabe and S. Hashimoto, *J. Am. Chem. Soc.*, 1999, **121**, 1417.
2. a) M. K. Choi, W. Yu and C. Che, *Org. Lett.*, 2005, **7**, 1081; (b) M. Grohmann, and G. Maas, *Tetrahedron*, 2007, **63**, 12172; (c) M. P. Doyle, M. S. Shanklin, S. M. Oon, H. Q. Pho, F. R. van der Heide and W. R. Veal, *J. Org. Chem.*, 1988, **53**, 3384.

Proton NMR Spectral Observation of Cyclopropene 6c

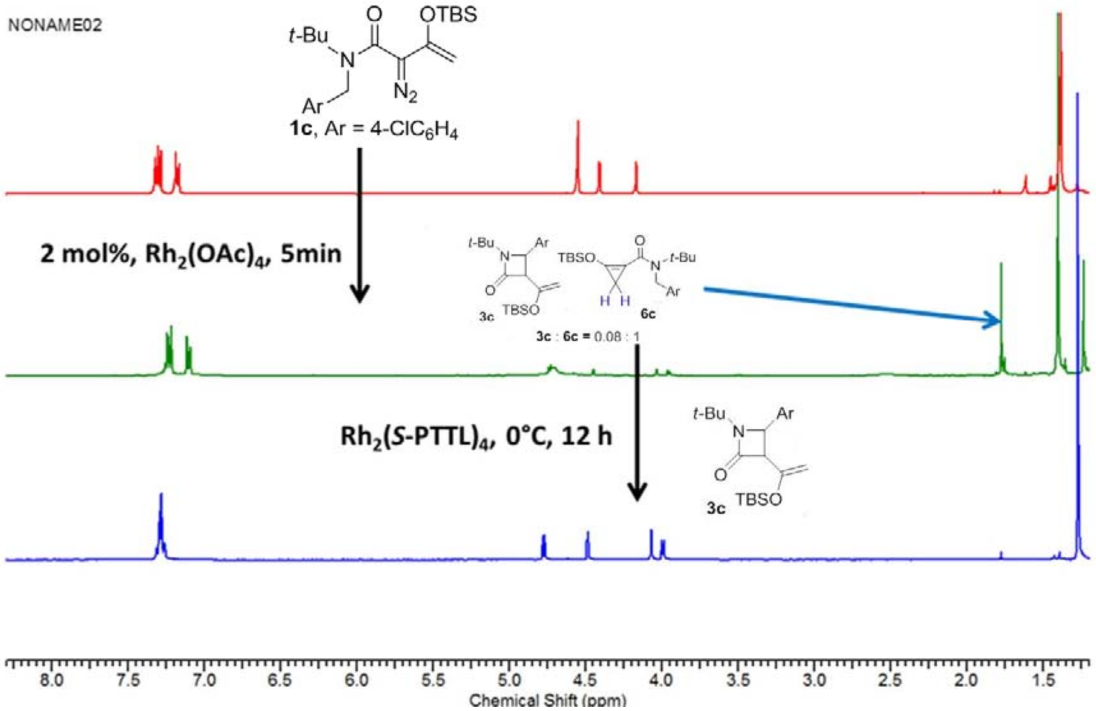

Chemoselectivity Determination by ^{1}H NMR Spectrum of the Reaction Mixture

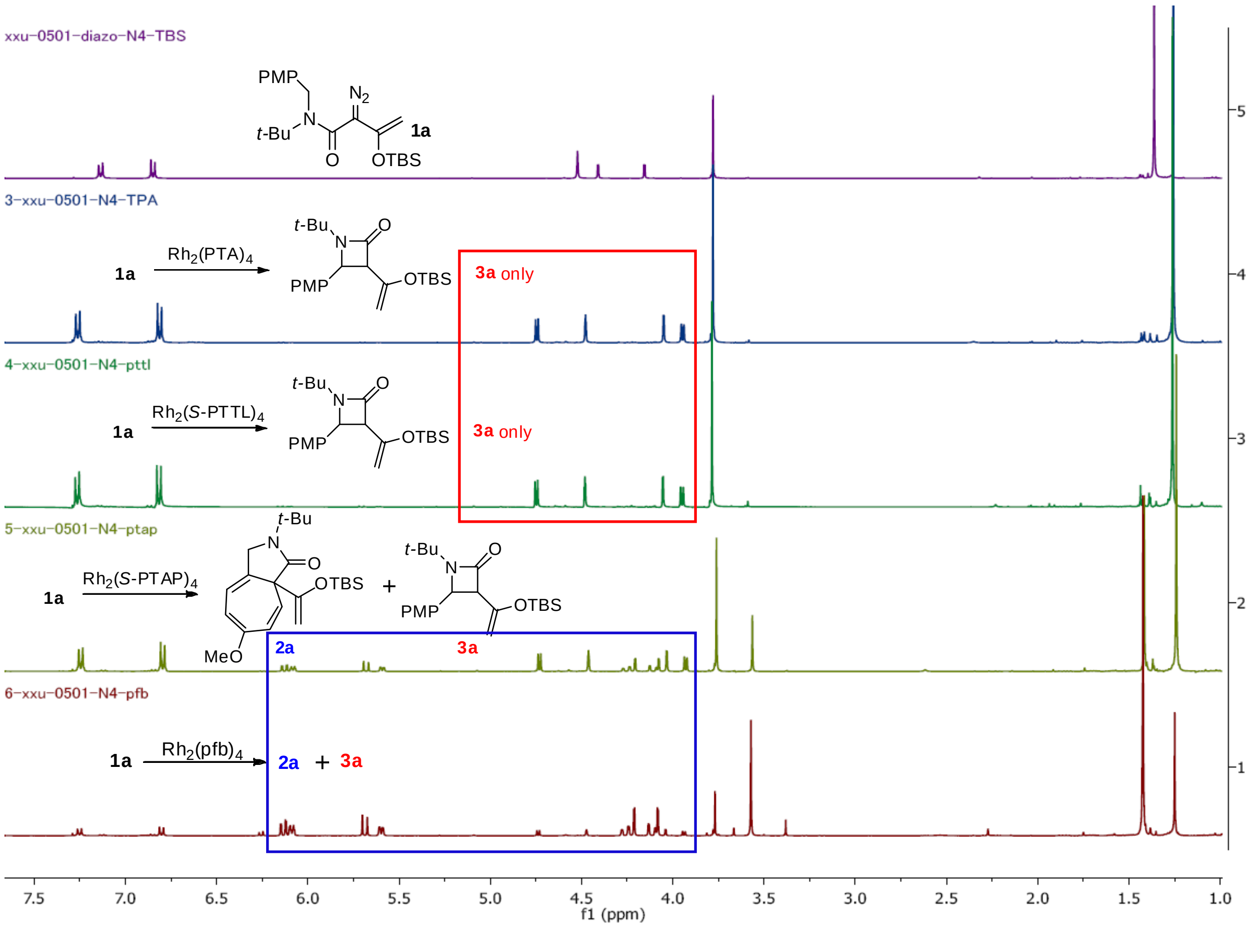

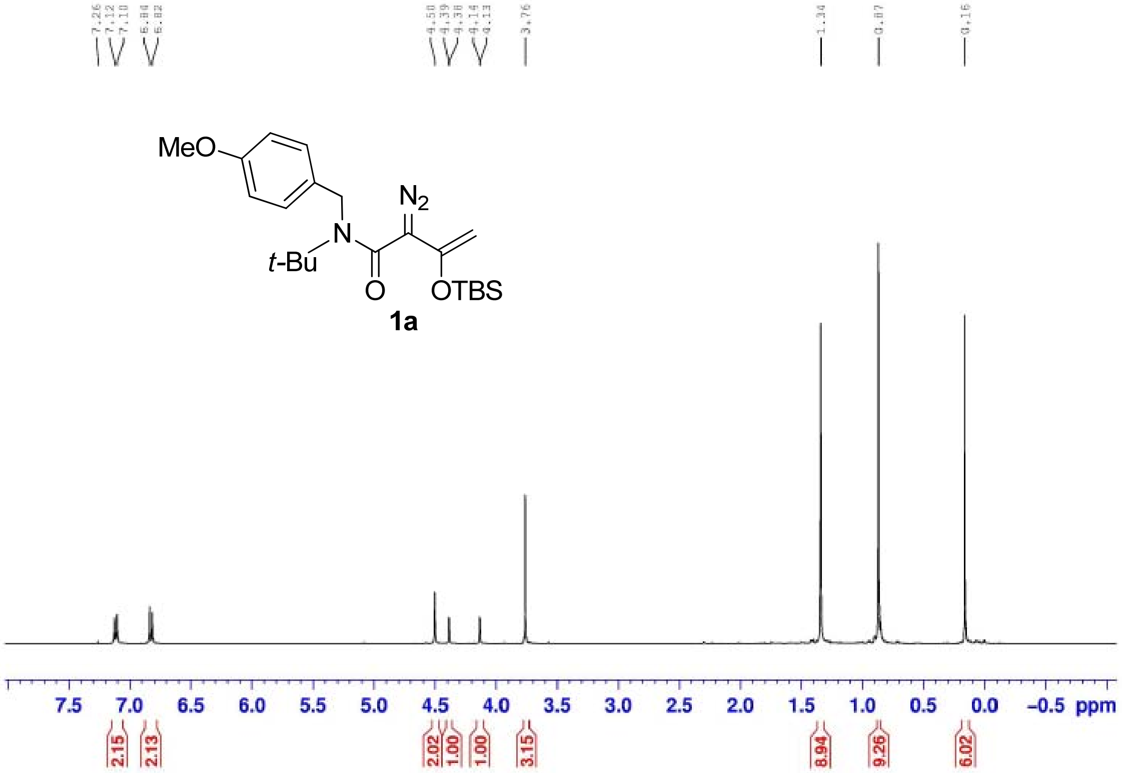

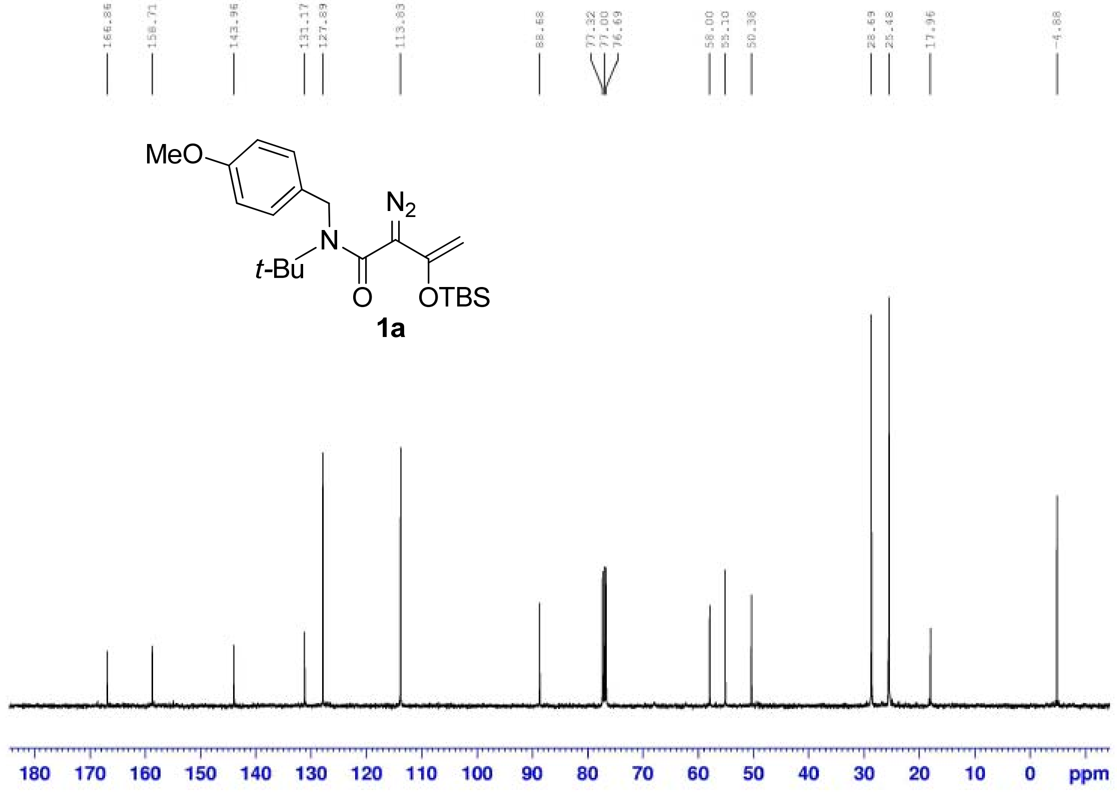

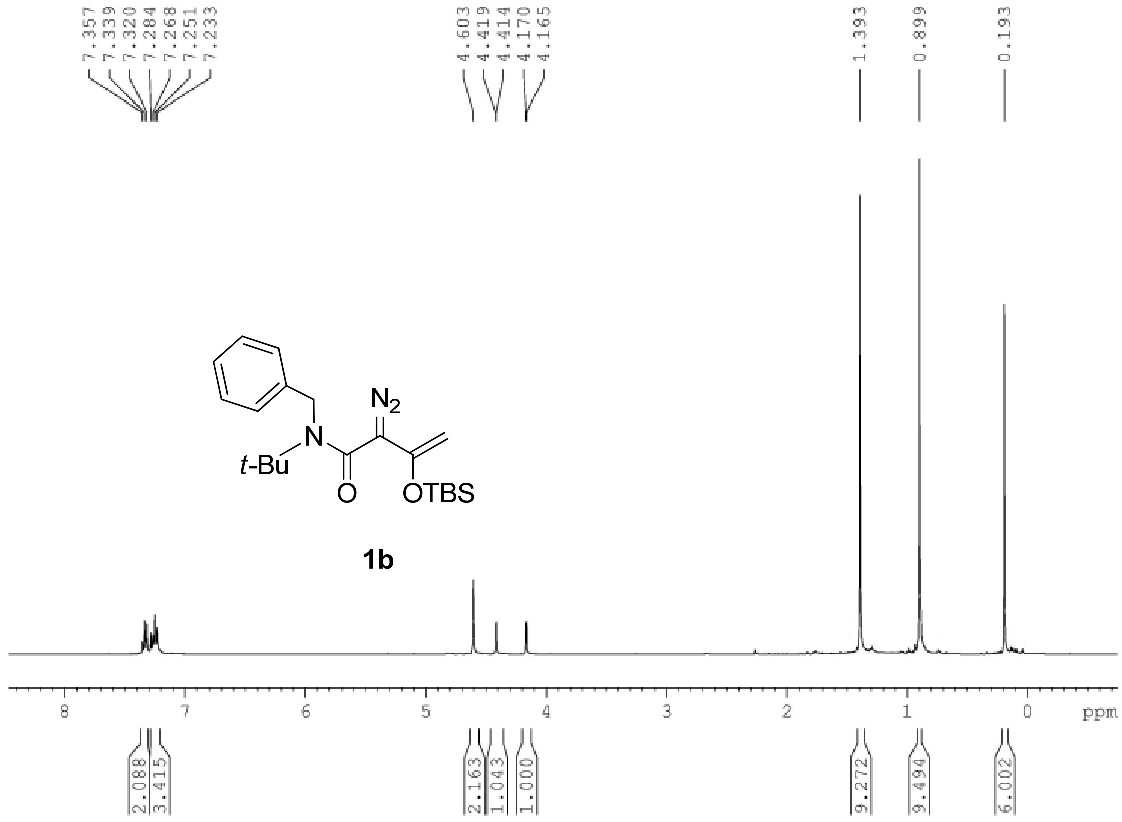
7.357
7.339
7.320
7.284
7.268
7.251
7.233
4.603
4.419
4.414
4.170
4.165
1.393
0.899
0.193
N2
t-Bu
N
O
OTBS
1b
8
7
6
5
4
3
2
1
0
ppm
2.088
3.415
2.163
1.043
1.000
9.272
9.494
6.002

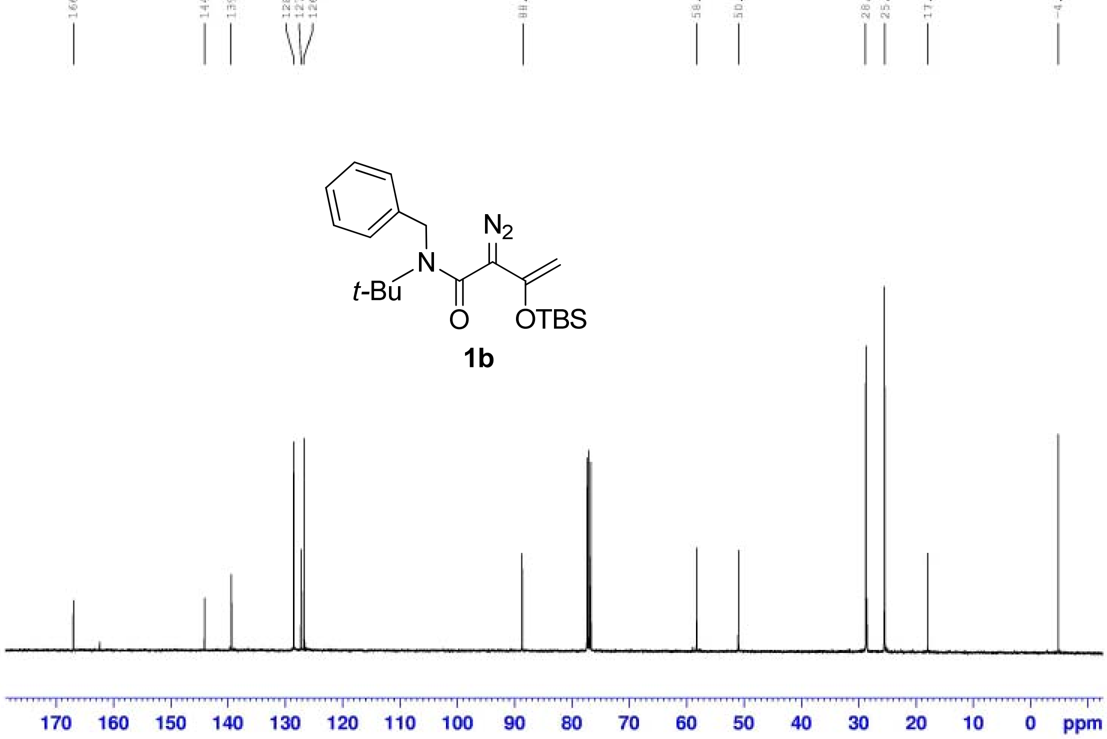
166.95
144.00
139.41
128.51
127.24
126.73
88.73
58.23
50.87
28.82
25.44
17.92
-4.79
N2
t-Bu
N
O
OTBS
1b
170
160
150
140
130
120
110
100
90
80
70
60
50
40
30
20
10
0
ppm

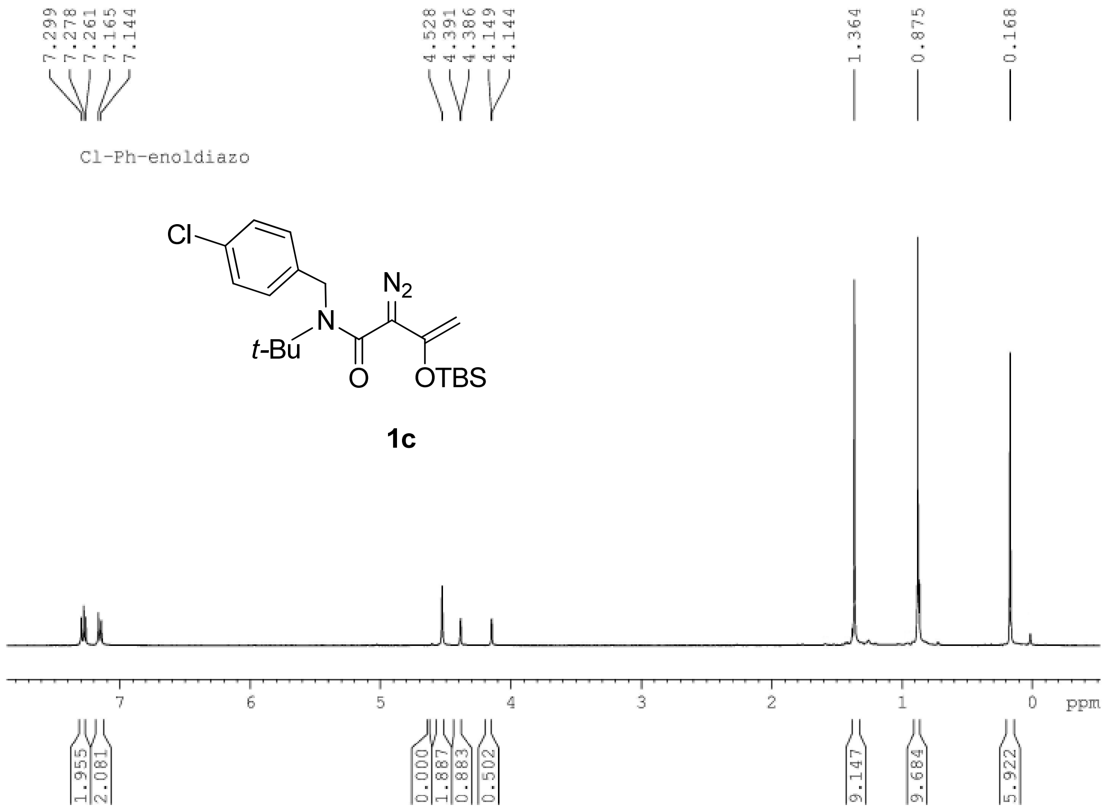
7.299
7.278
7.261
7.165
7.144
4.528
4.391
4.386
4.149
4.144
1.364
0.875
0.168
Cl-Ph-enoldiazo
Cl
N
N_2
t-Bu
O
OTBS
1c
7
6
5
4
3
2
1
0
ppm
1.955
2.081
0.000
1.887
0.883
0.502
9.147
9.684
5.922

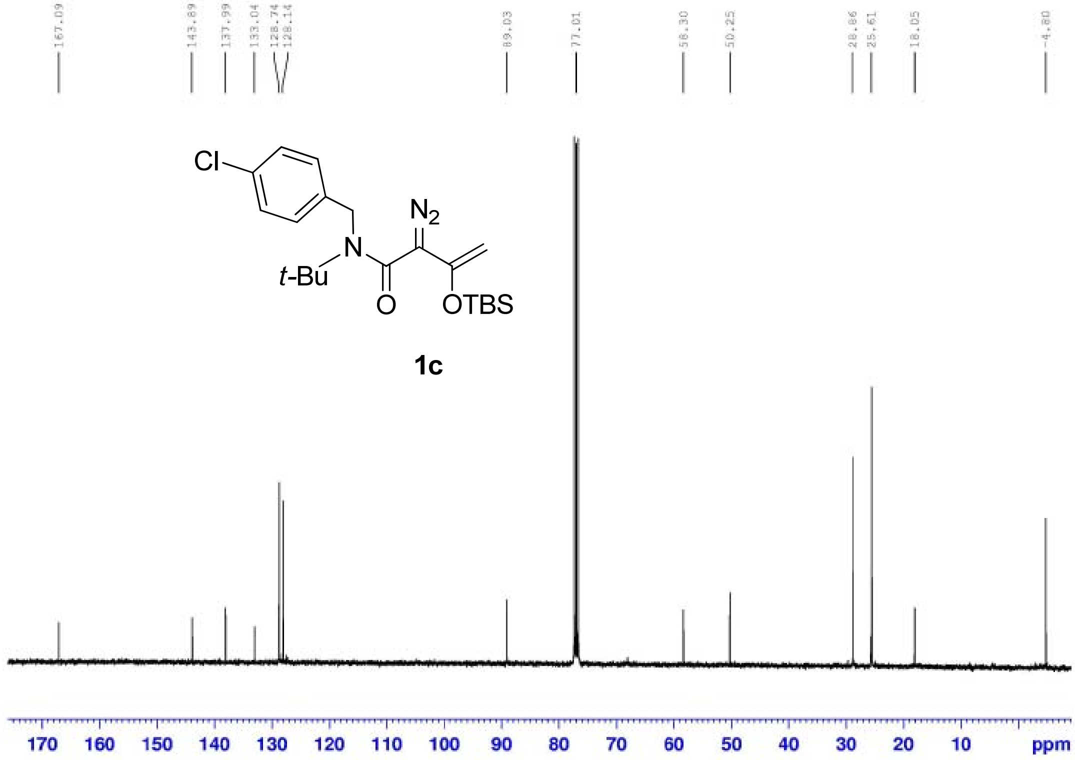
167.09
143.89
137.99
133.04
128.74
128.14
89.03
77.01
58.30
50.25
28.86
25.61
18.05
-4.80
Cl
N
N_2
t-Bu
O
OTBS
1c
170
160
150
140
130
120
110
100
90
80
70
60
50
40
30
20
10
ppm

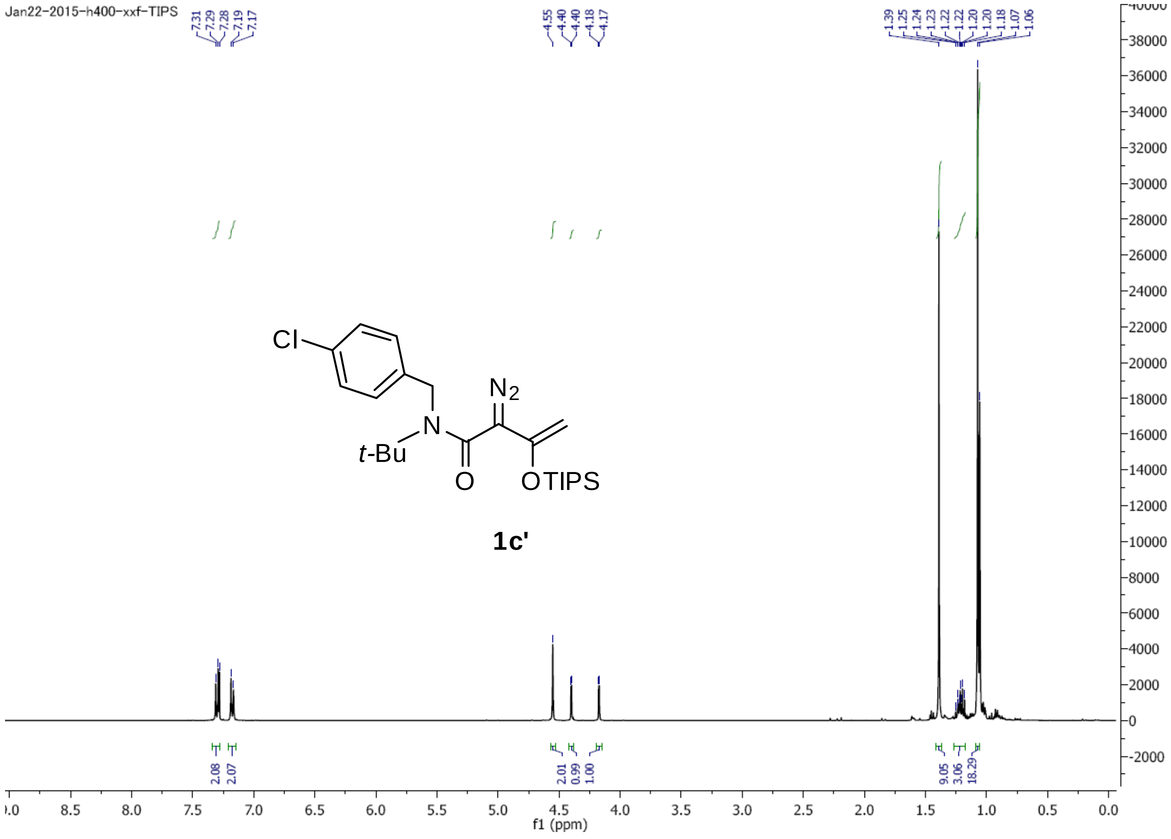
Jan22-2015-h400-xxf-TIPS
Cl
N
N2
t-Bu
O
OTIPS
1c'
f1 (ppm)

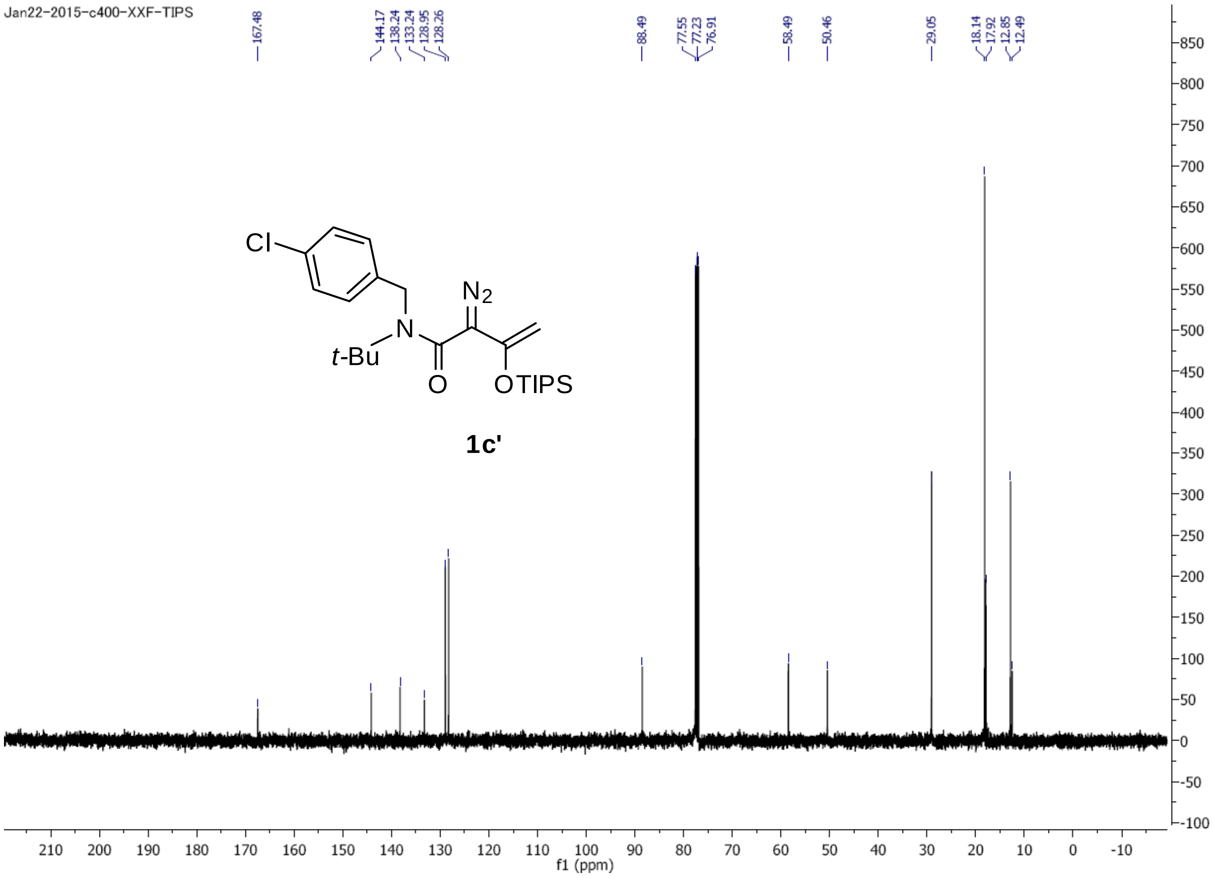
Jan22-2015-c400-XXF-TIPS
Cl
N
N2
t-Bu
O
OTIPS
1c'
f1 (ppm)

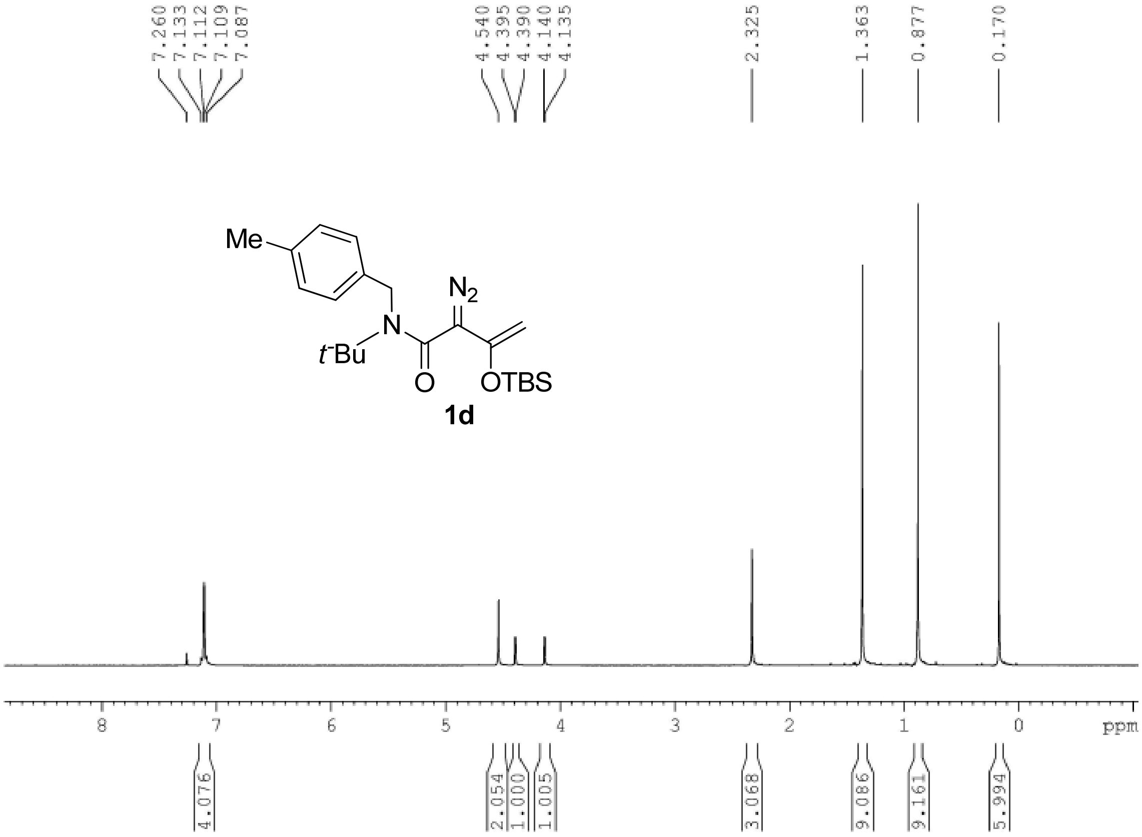

1d

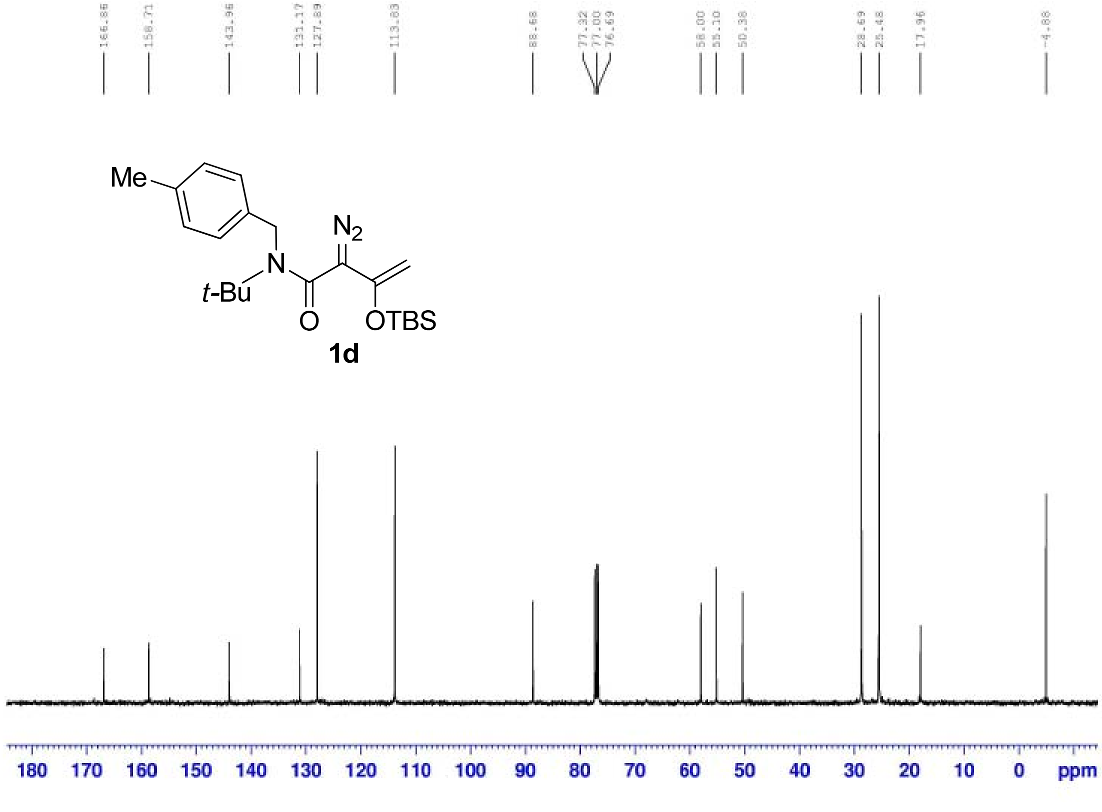

1d

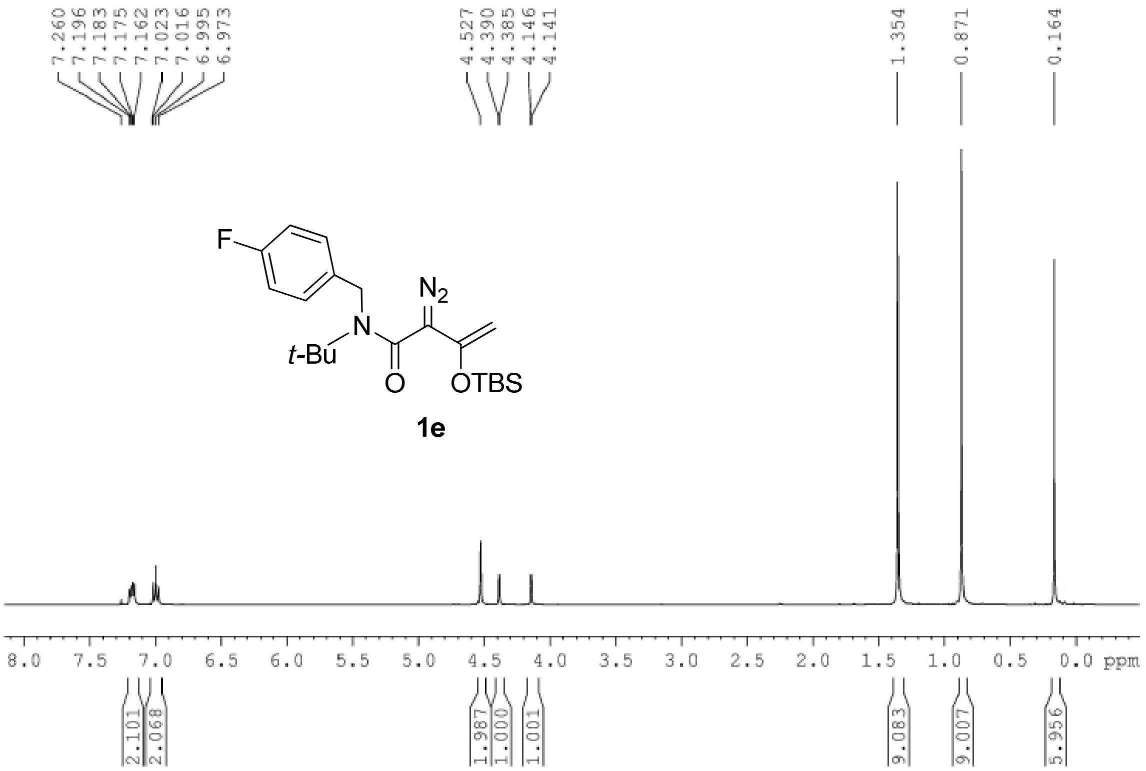
7.260
7.196
7.183
7.175
7.162
7.023
7.016
6.995
6.973
4.527
4.390
4.385
4.146
4.141
1.354
0.871
0.164
F
N$_2$
N
t-Bu
O
OTBS
1e
ppm
2.101
2.068
1.987
1.000
1.001
9.083
9.007
5.956

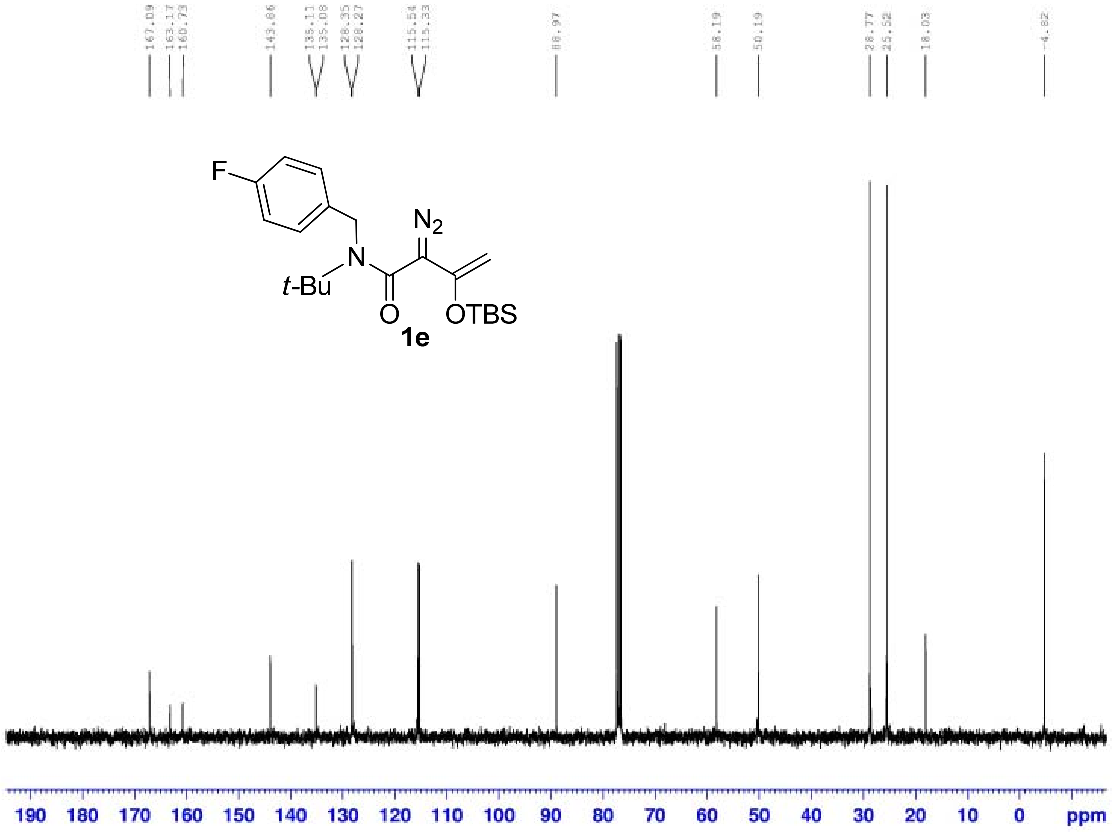
167.09
163.17
160.73
143.86
135.11
135.08
128.35
128.27
115.54
115.33
88.97
58.19
50.19
28.77
25.52
18.03
-4.82
F
N$_2$
N
t-Bu
O
OTBS
1e
ppm

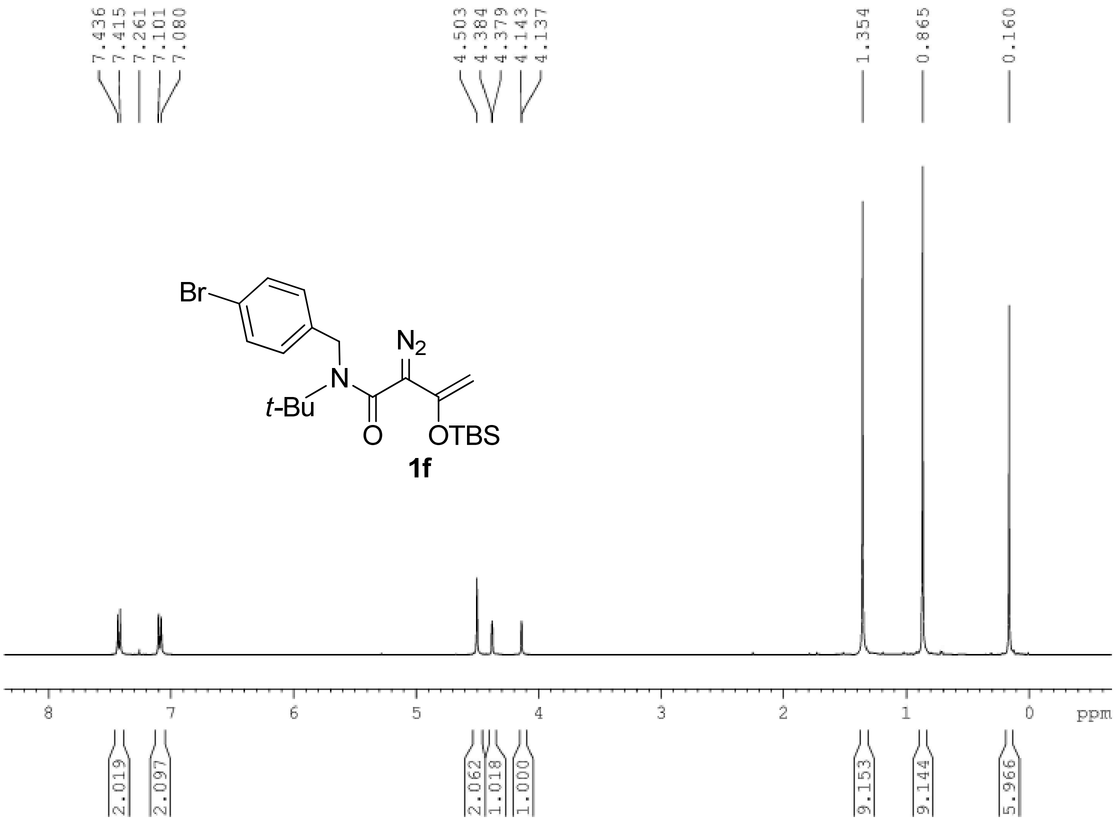
7.436
7.415
7.261
7.101
7.080
4.503
4.384
4.379
4.143
4.137
1.354
0.865
0.160
Br
N2
N
t-Bu
O
OTBS
1f
8
7
6
5
4
3
2
1
0
ppm
2.019
2.097
2.062
1.018
1.000
9.153
9.144
5.966

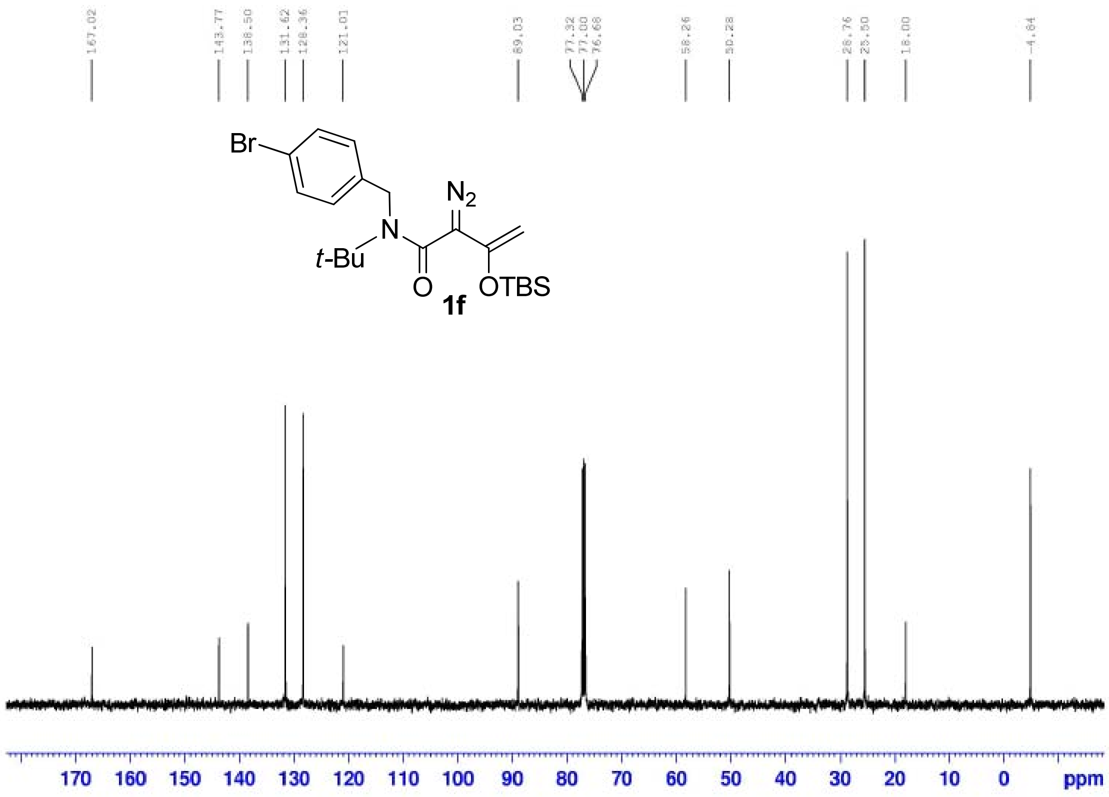
167.02
143.77
138.50
131.62
128.36
121.01
89.03
77.32
77.00
76.68
58.26
50.28
28.76
25.50
18.00
-4.84
Br
N2
N
t-Bu
O
OTBS
1f
170
160
150
140
130
120
110
100
90
80
70
60
50
40
30
20
10
0
ppm

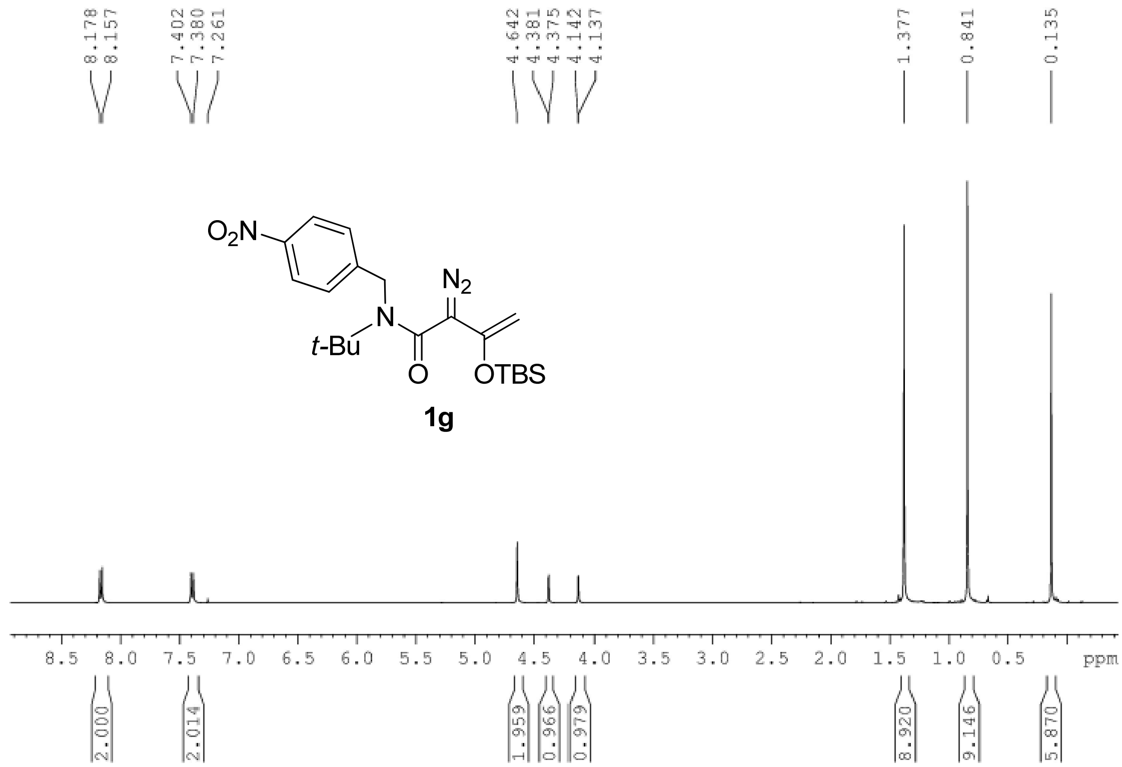
8.178
8.157
7.402
7.380
7.261
4.642
4.381
4.375
4.142
4.137
1.377
0.841
0.135
O_2N
N_2
t-Bu
N
O
OTBS
1g
8.5 8.0 7.5 7.0 6.5 6.0 5.5 5.0 4.5 4.0 3.5 3.0 2.5 2.0 1.5 1.0 0.5 ppm
2.000
2.014
1.959
0.966
0.979
8.920
9.146
5.870

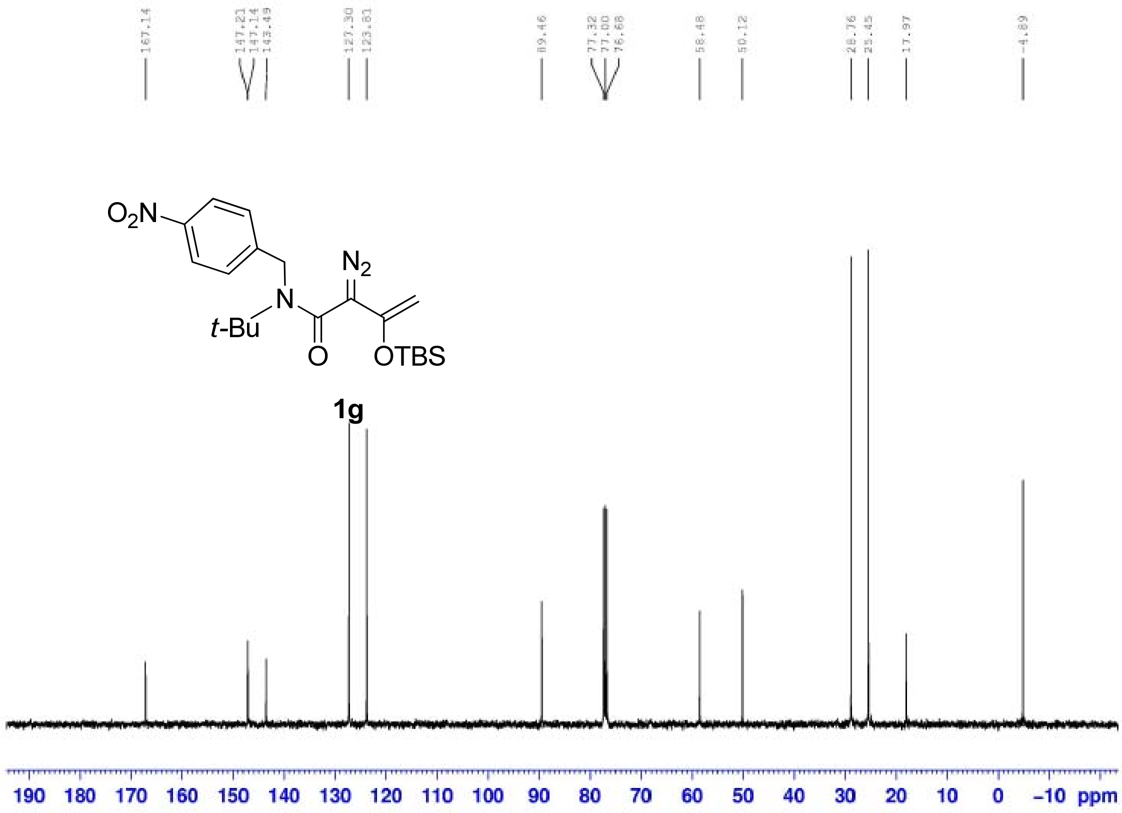
167.14
147.21
147.14
143.49
127.30
123.81
89.46
77.32
77.00
76.68
58.48
50.12
28.76
25.45
17.97
-4.89
O_2N
N_2
t-Bu
N
O
OTBS
1g
190 180 170 160 150 140 130 120 110 100 90 80 70 60 50 40 30 20 10 0 –10 ppm

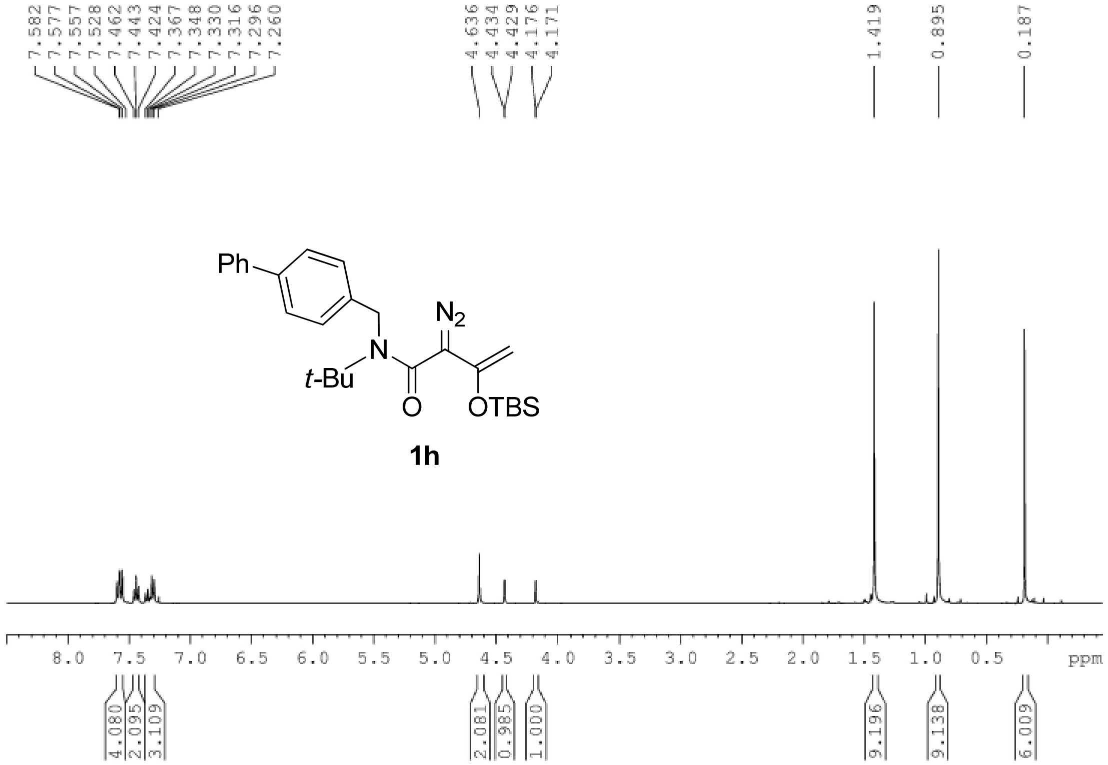
7.582
7.577
7.557
7.528
7.462
7.443
7.424
7.367
7.348
7.330
7.316
7.296
7.260
4.636
4.434
4.429
4.176
4.171
1.419
0.895
0.187
Ph
N$_2$
N
t-Bu
O
OTBS
1h
8.0 7.5 7.0 6.5 6.0 5.5 5.0 4.5 4.0 3.5 3.0 2.5 2.0 1.5 1.0 0.5 ppm
4.080
2.095
3.109
2.081
0.985
1.000
9.196
9.138
6.009

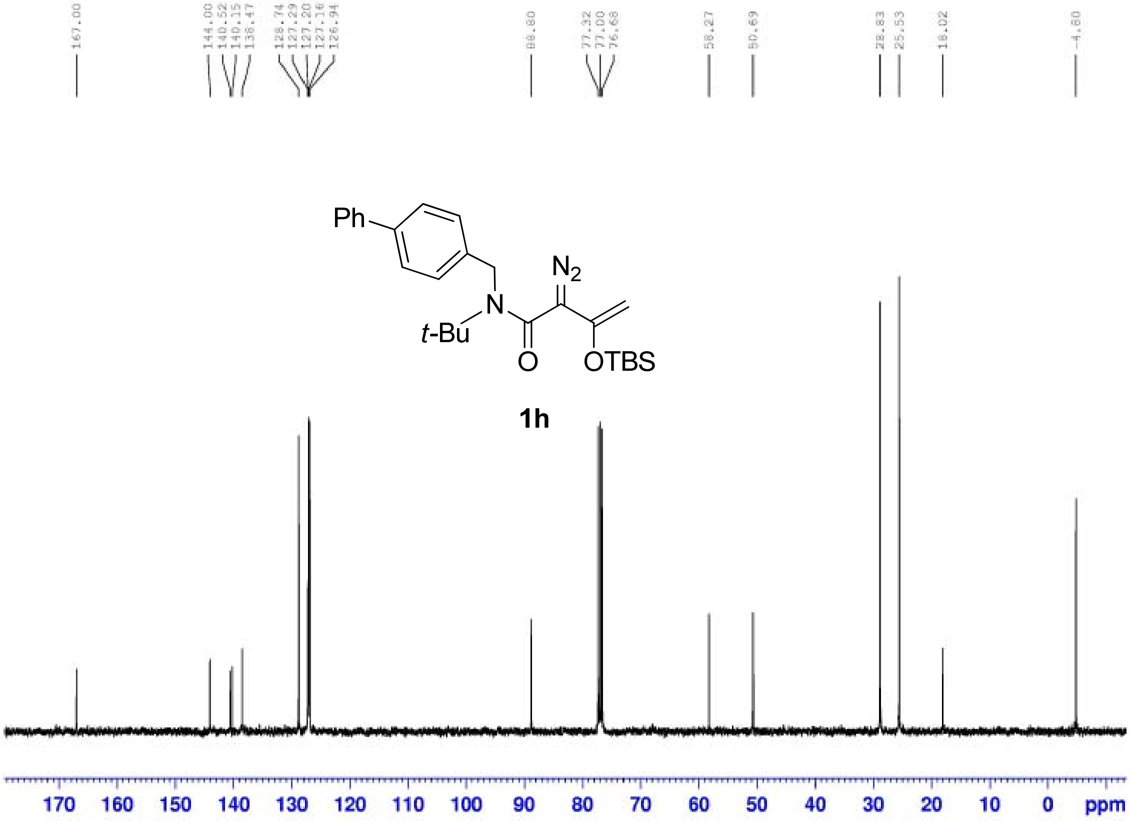
167.00
144.00
140.52
140.15
138.47
128.74
127.29
127.20
127.16
126.94
88.80
77.32
77.00
76.68
58.27
50.69
28.83
25.53
18.02
-4.80
Ph
N$_2$
N
t-Bu
O
OTBS
1h
170 160 150 140 130 120 110 100 90 80 70 60 50 40 30 20 10 0 ppm

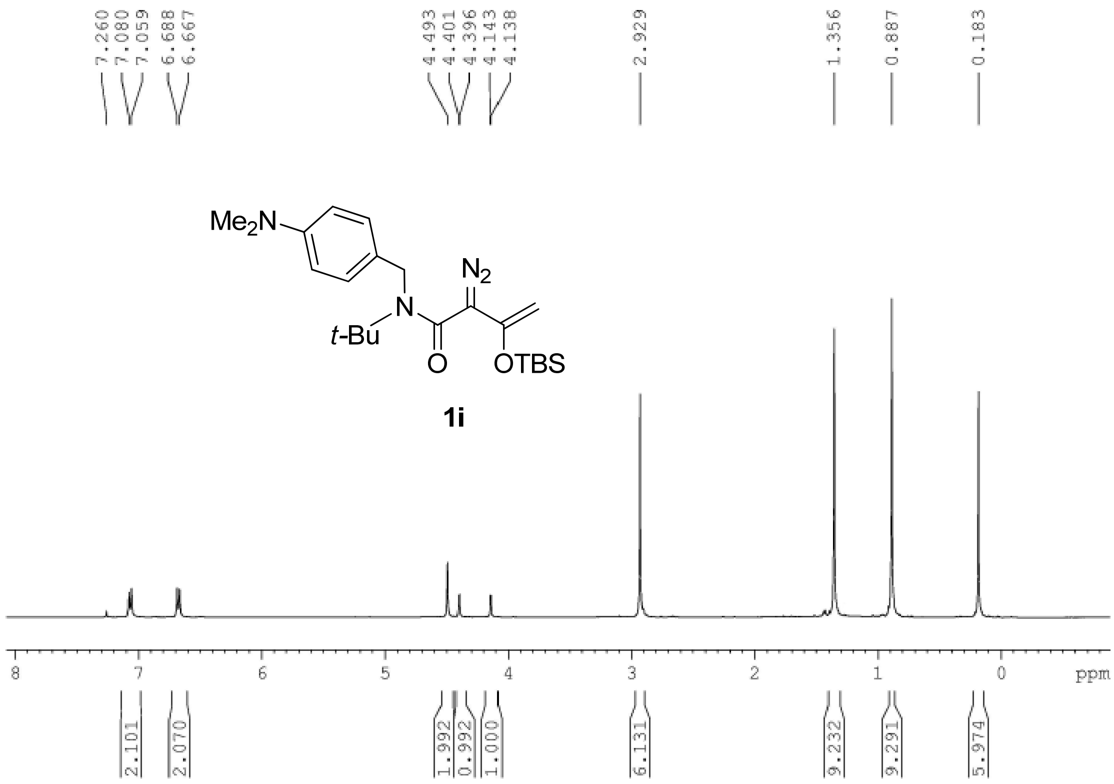
7.260
7.080
7.059
6.688
6.667
4.493
4.401
4.396
4.143
4.138
2.929
1.356
0.887
0.183
Me2N
N2
t-Bu
N
O
OTBS
1i
ppm
2.101
2.070
1.992
0.992
1.000
6.131
9.232
9.291
5.974

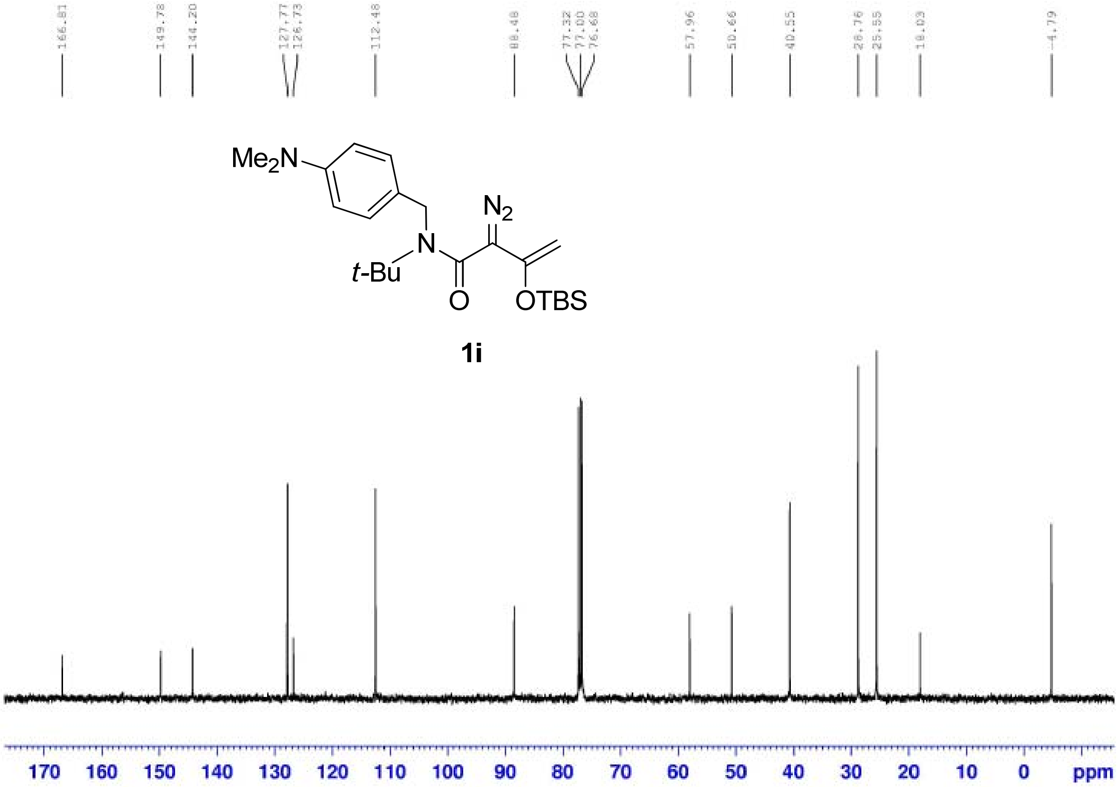
166.81
149.78
144.20
127.77
126.73
112.48
88.48
77.32
77.00
76.68
57.96
50.66
40.55
28.76
25.55
18.03
-4.79
Me2N
N2
t-Bu
N
O
OTBS
1i
ppm

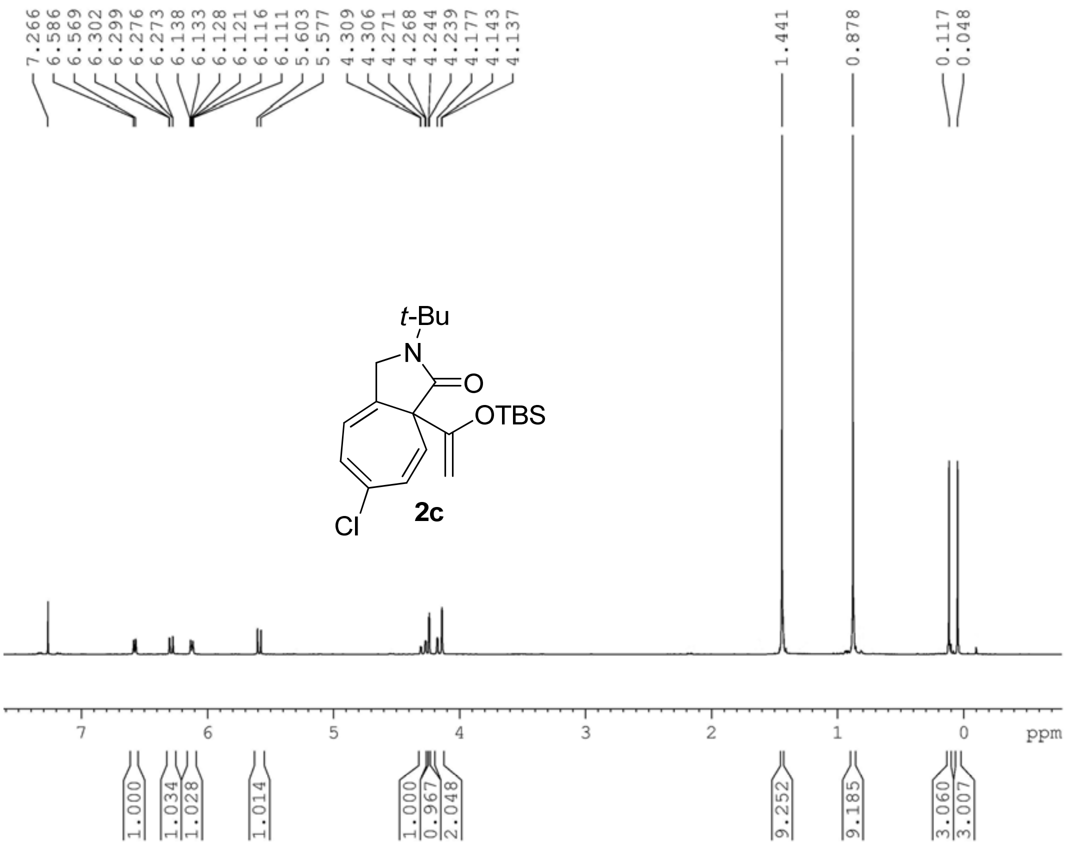
7.266
6.586
6.569
6.302
6.299
6.276
6.273
6.138
6.133
6.128
6.121
6.116
6.111
5.603
5.577
4.309
4.306
4.271
4.268
4.244
4.239
4.177
4.143
4.137
1.441
0.878
0.117
0.048
t-Bu
N
O
OTBS
Cl
2c
ppm
1.000
1.034
1.028
1.014
1.000
0.967
2.048
9.252
9.185
3.060
3.007

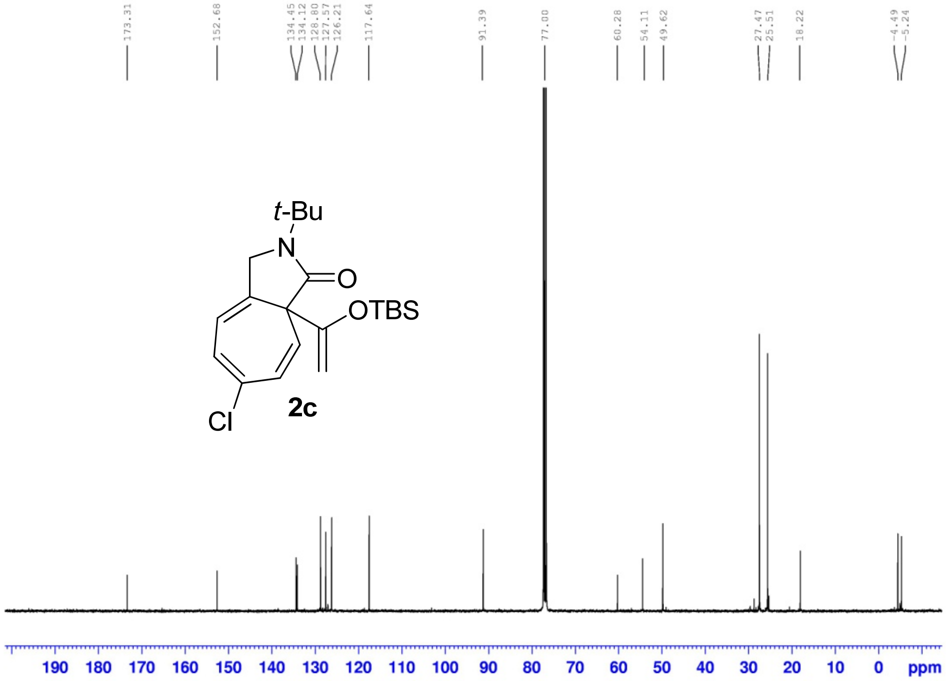
173.31
152.68
134.45
134.12
128.80
127.57
126.21
117.64
91.39
77.00
60.28
54.11
49.62
27.47
25.51
18.22
-4.49
-5.24
t-Bu
N
O
OTBS
Cl
2c
190 180 170 160 150 140 130 120 110 100 90 80 70 60 50 40 30 20 10 0 ppm

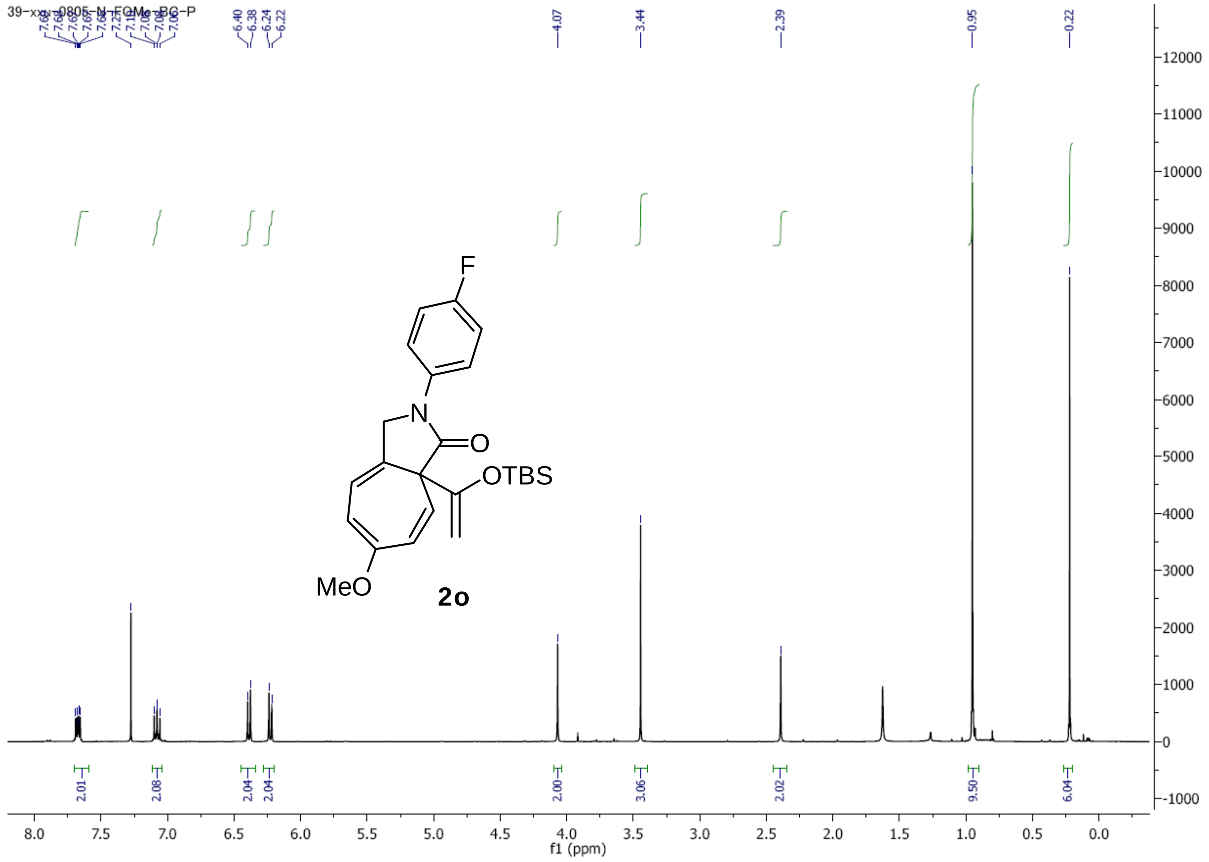

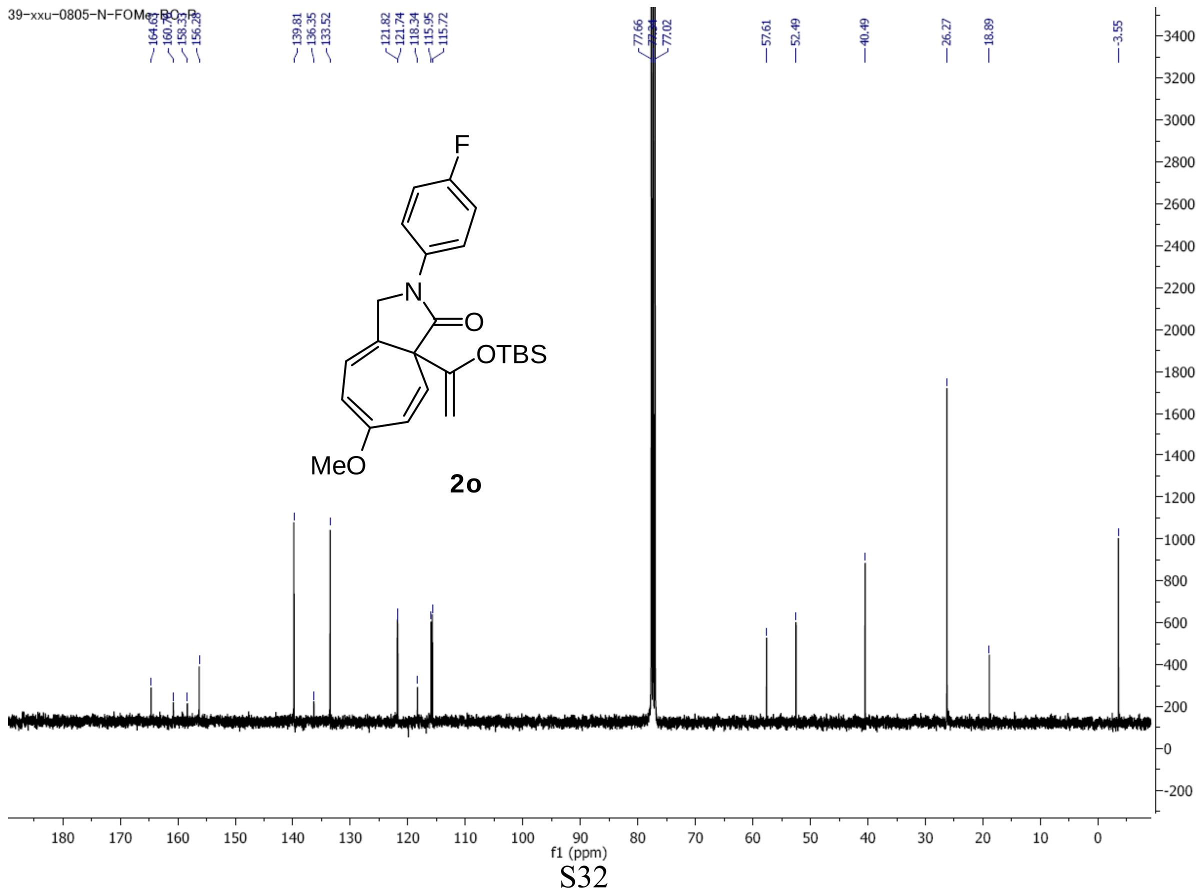

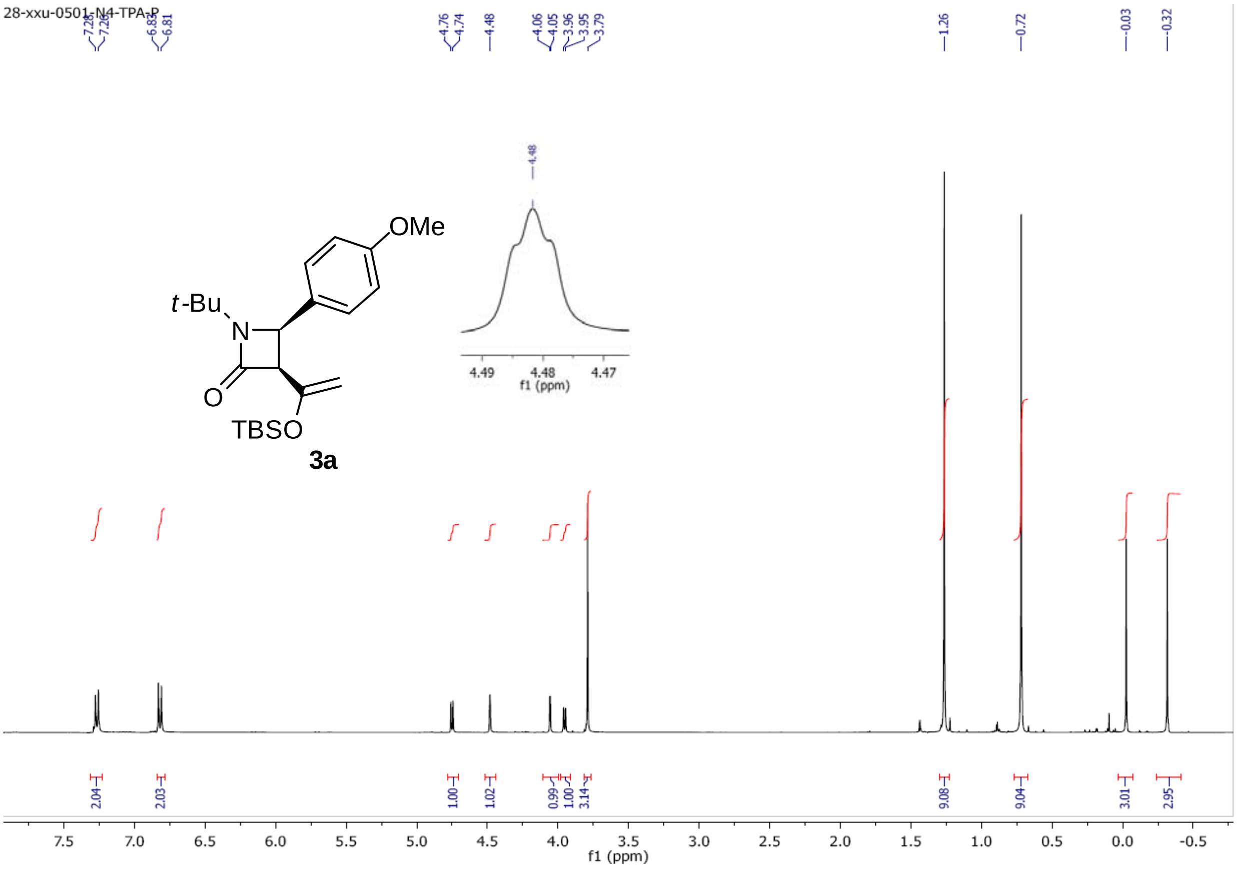
28-xxu-0501-N4-TPA-P
OMe
t-Bu
N
O
TBSO
3a
4.49
4.48
4.47
f1 (ppm)
2.04
2.03
1.00
1.02
0.99
1.00
3.14
9.08
9.04
3.01
2.95
7.5
7.0
6.5
6.0
5.5
5.0
4.5
4.0
3.5
3.0
2.5
2.0
1.5
1.0
0.5
0.0
-0.5
f1 (ppm)

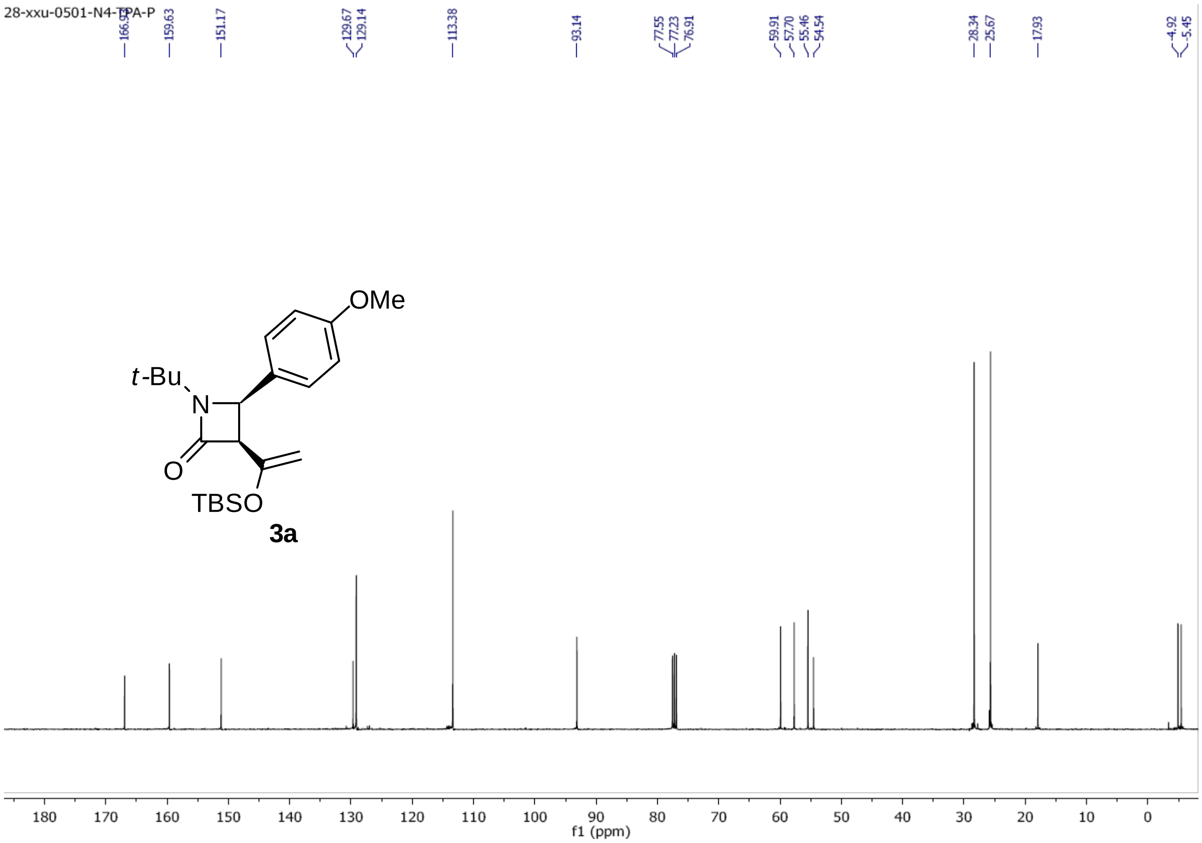
28-xxu-0501-N4-TPA-P
159.63
151.17
129.67
129.14
113.38
93.14
77.55
77.23
76.91
59.91
57.70
55.46
54.54
28.34
25.67
17.93
-4.92
-5.45
OMe
t-Bu
N
O
TBSO
3a
180
170
160
150
140
130
120
110
100
90
80
70
60
50
40
30
20
10
0
f1 (ppm)

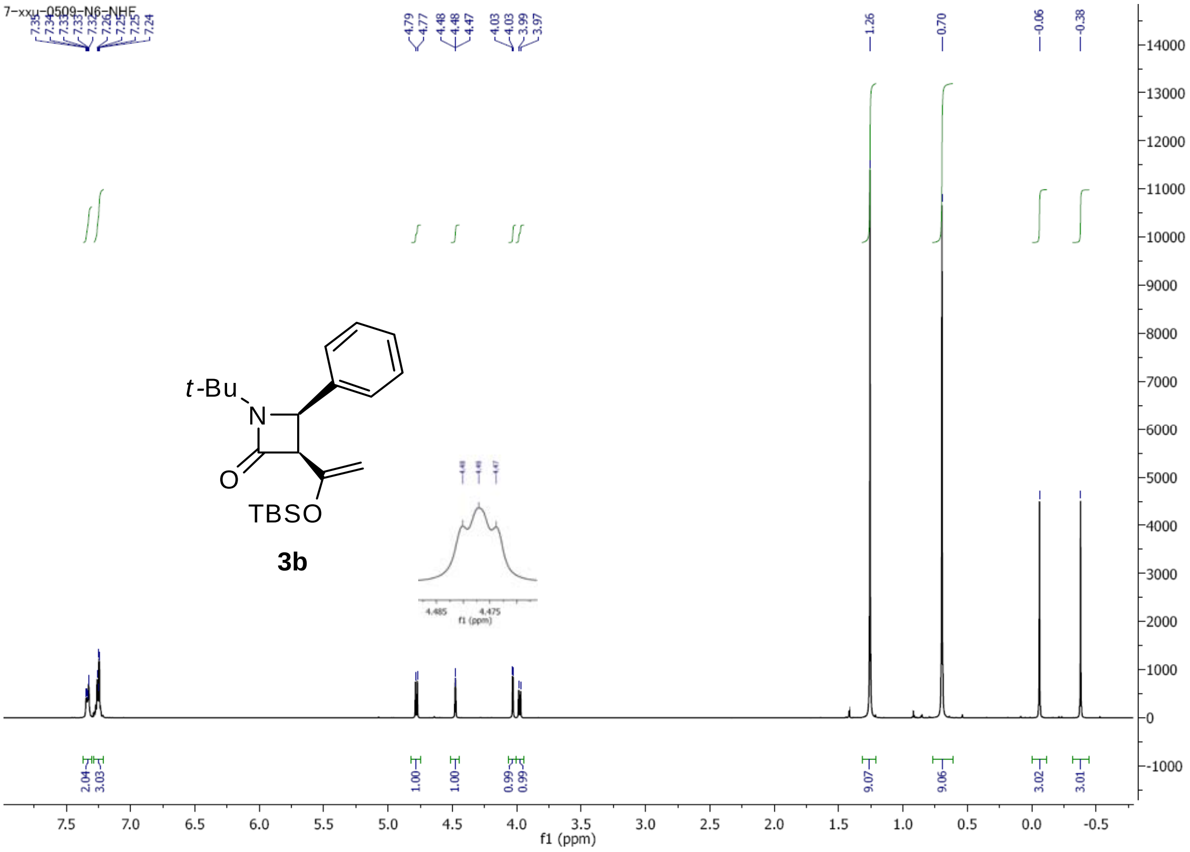

7-xxu-0509-N6-NHF
t-Bu
N
O
TBSO
3b
f1 (ppm)

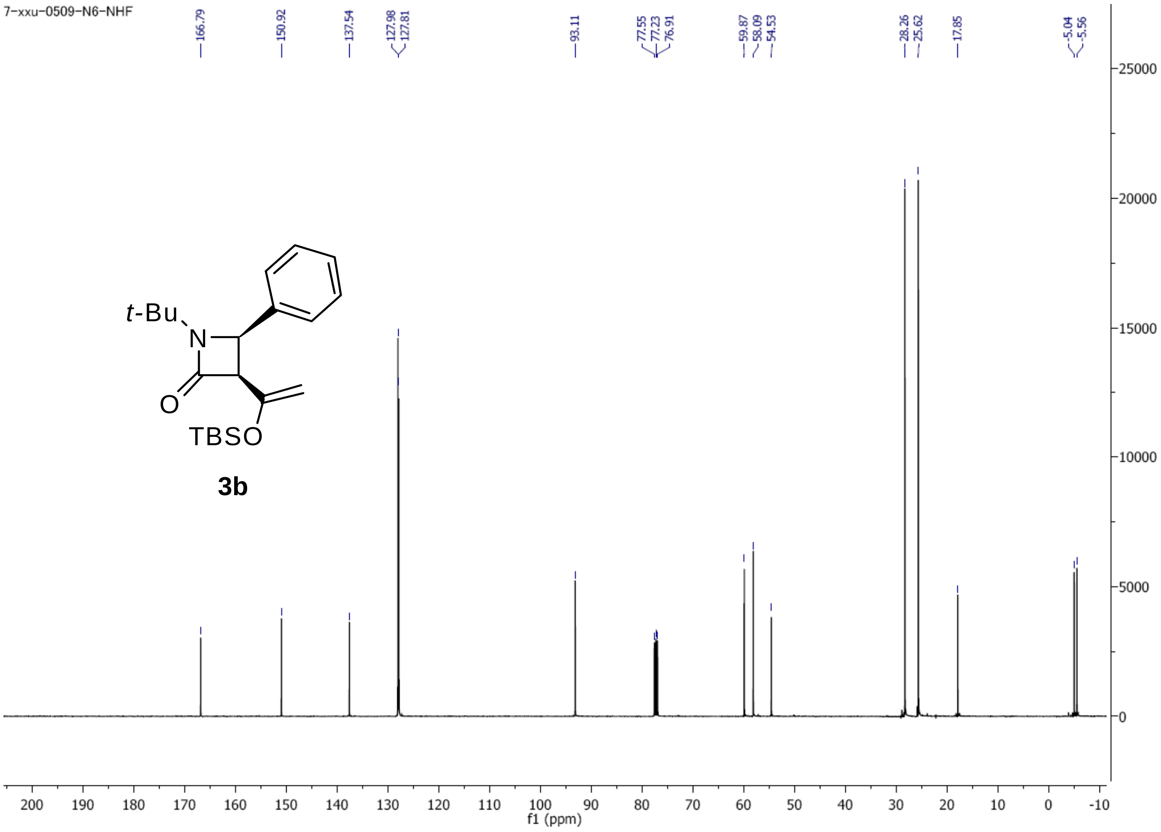

7-xxu-0509-N6-NHF
166.79
150.92
137.54
127.98
127.81
93.11
77.55
77.23
76.91
59.87
58.09
54.53
28.26
25.62
17.85
-5.04
-5.56
t-Bu
N
O
TBSO
3b
f1 (ppm)

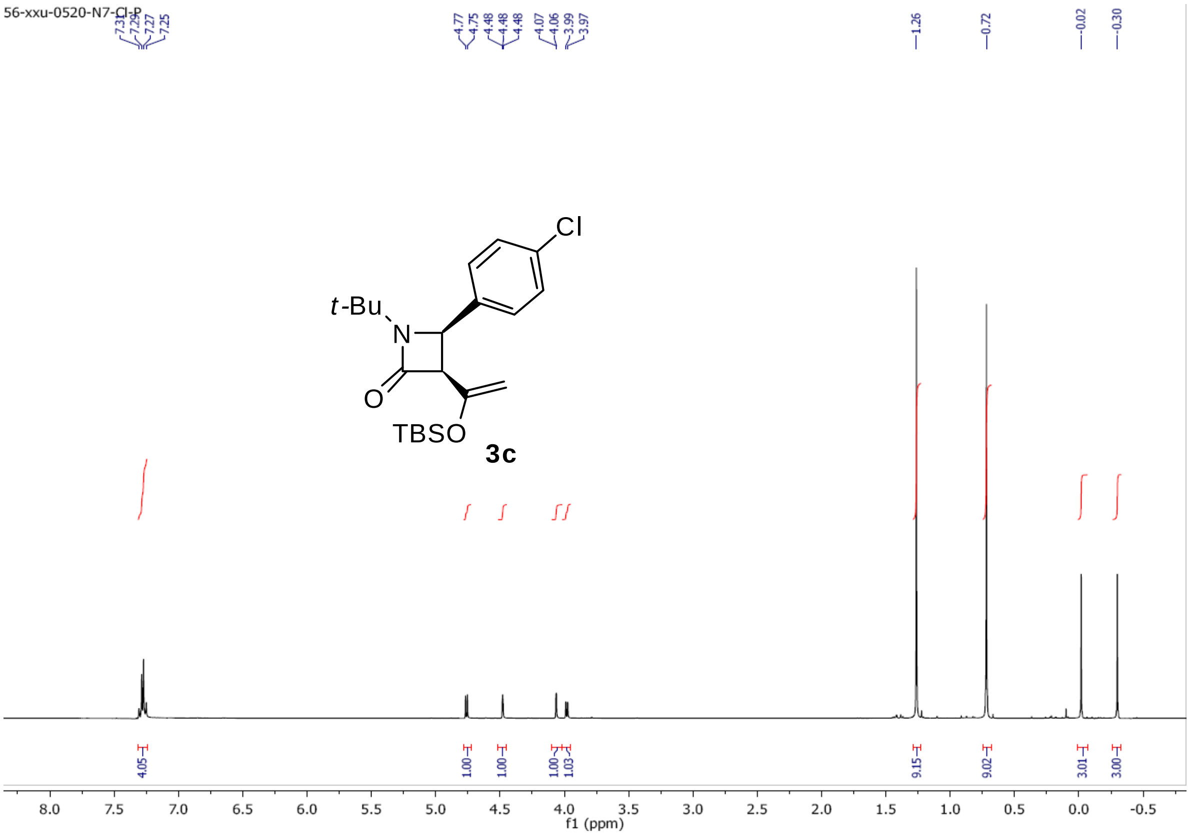
56-xxu-0520-N7-Cl-P
7.31
7.29
7.27
7.25
4.77
4.75
4.48
4.48
4.48
4.07
4.06
3.99
3.97
1.26
0.72
-0.02
-0.30
Cl
t-Bu
N
O
TBSO
3c
4.05
1.00
1.00
1.00
1.03
9.15
9.02
3.01
3.00
8.0 7.5 7.0 6.5 6.0 5.5 5.0 4.5 4.0 3.5 3.0 2.5 2.0 1.5 1.0 0.5 0.0 -0.5
f1 (ppm)

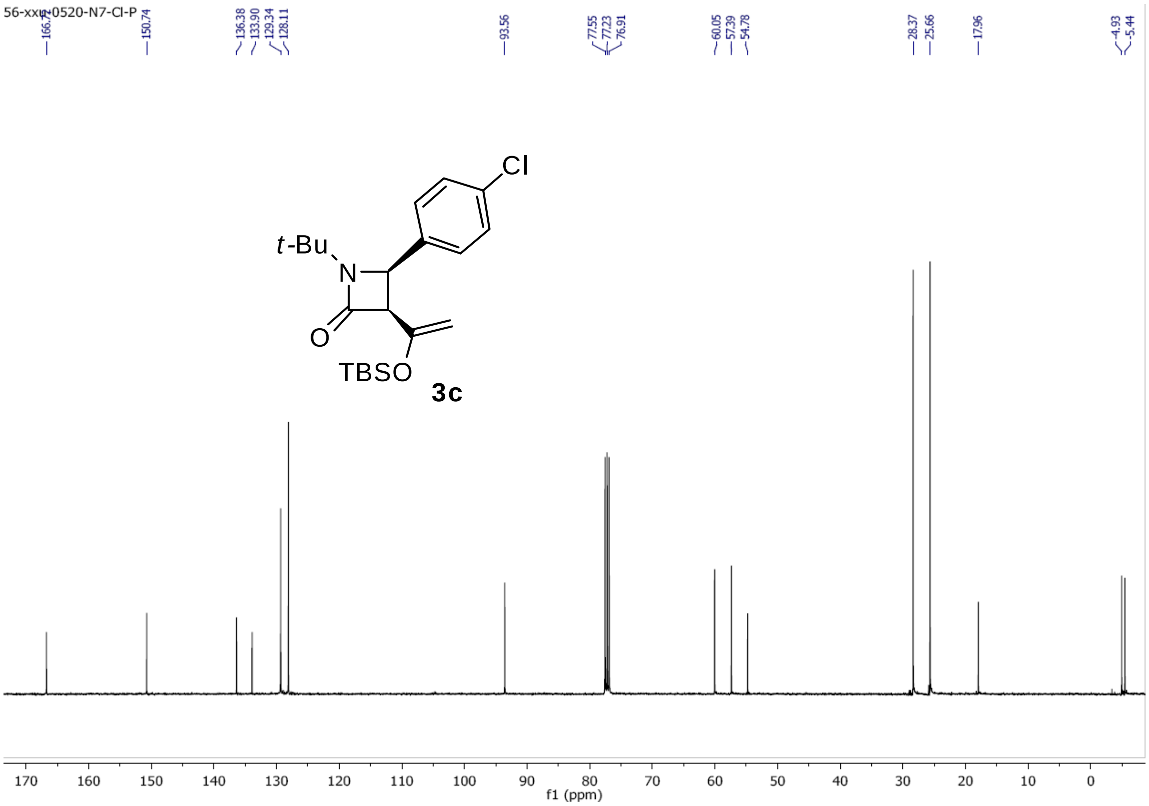
56-xxu-0520-N7-Cl-P
166.74
150.74
136.38
133.90
129.34
128.11
93.56
77.55
77.23
76.91
60.05
57.39
54.78
28.37
25.66
17.96
-4.93
-5.44
Cl
t-Bu
N
O
TBSO
3c
170 160 150 140 130 120 110 100 90 80 70 60 50 40 30 20 10 0
f1 (ppm)

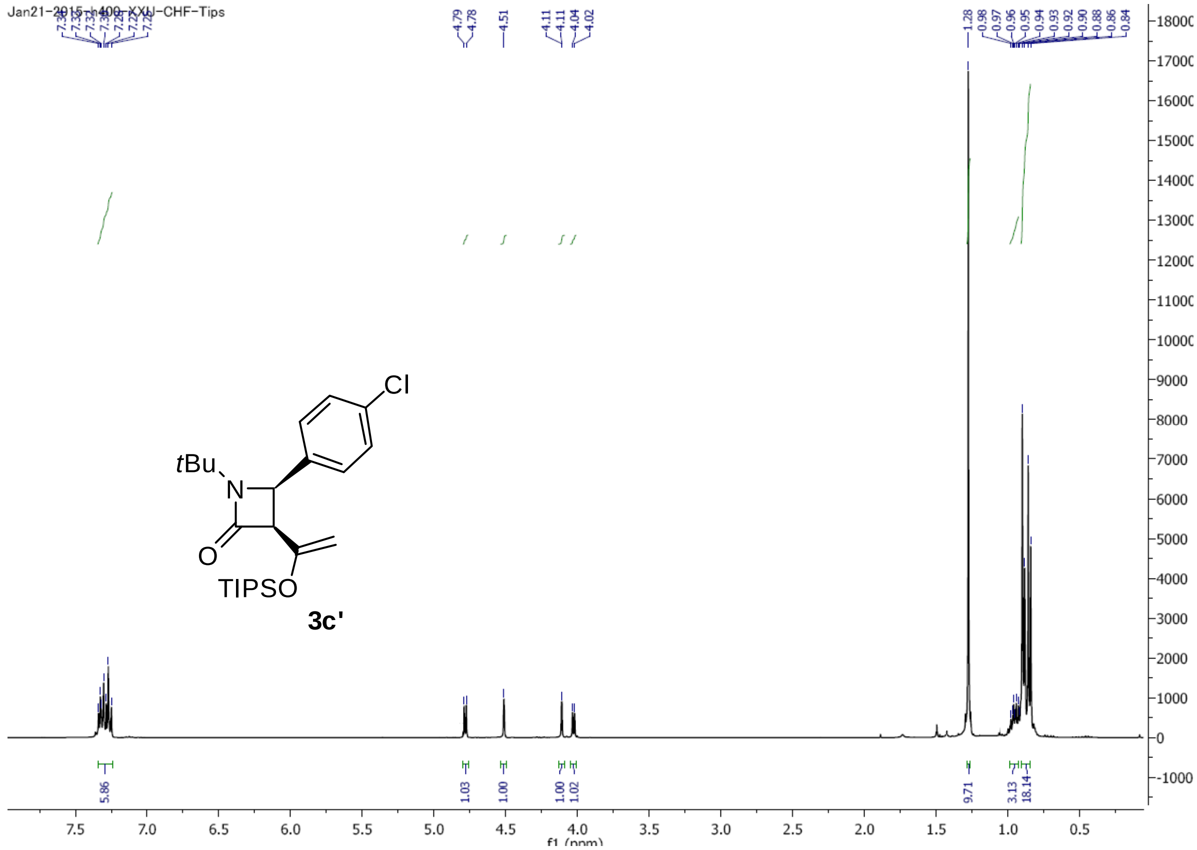
Jan21-2015-h400-XXU-CHF-Tips
4.79 4.78 4.51 4.11 4.11 4.04 4.02
1.28 0.98 0.97 0.96 0.95 0.94 0.93 0.92 0.90 0.88 0.86 0.84
Cl
tBu
N
O
TIPSO
3c'
5.86 1.03 1.00 1.00 1.02 9.71 3.13 18.14
f1 (ppm)

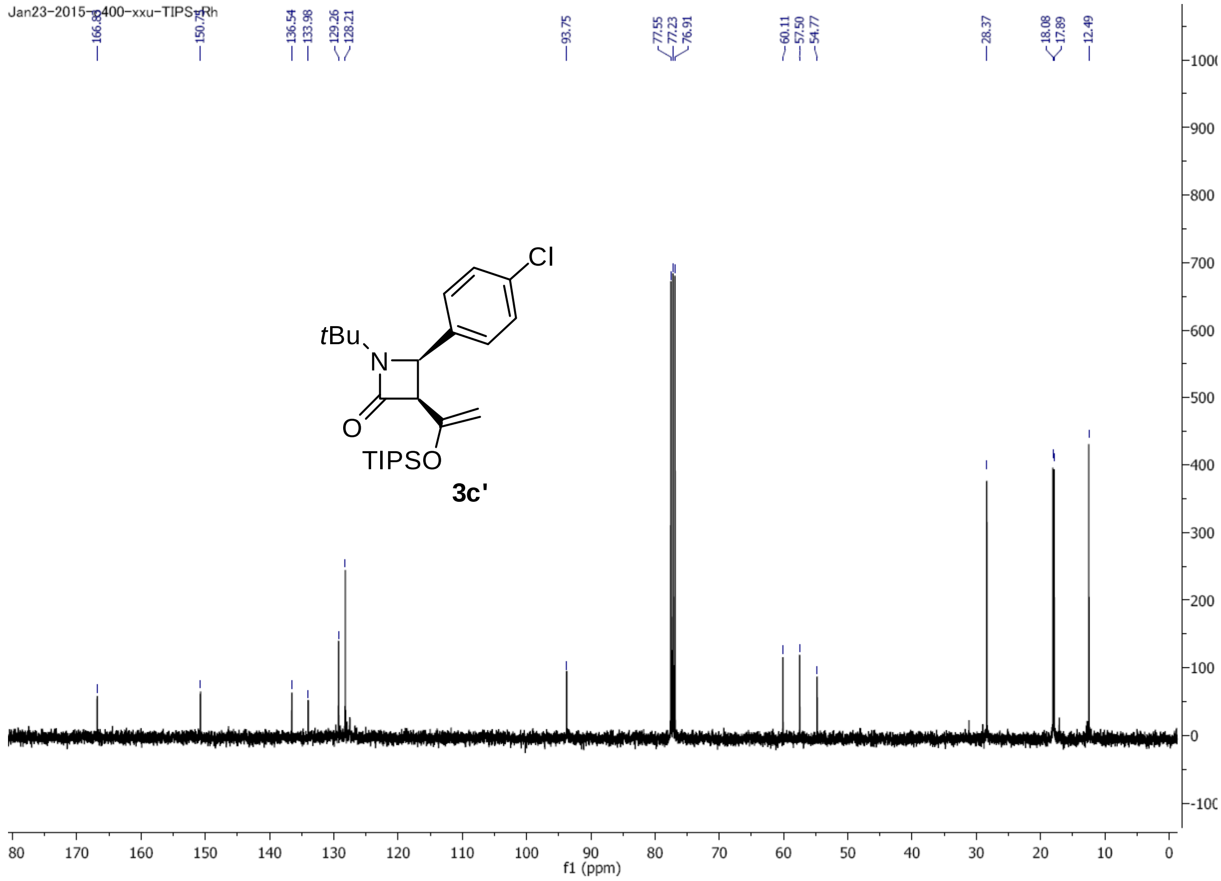
Jan23-2015-c400-xxu-TIPS-Rh
136.54 133.98 129.26 128.21 93.75 77.55 77.23 76.91 60.11 57.50 54.77 28.37 18.08 17.89 12.49
Cl
tBu
N
O
TIPSO
3c'
f1 (ppm)

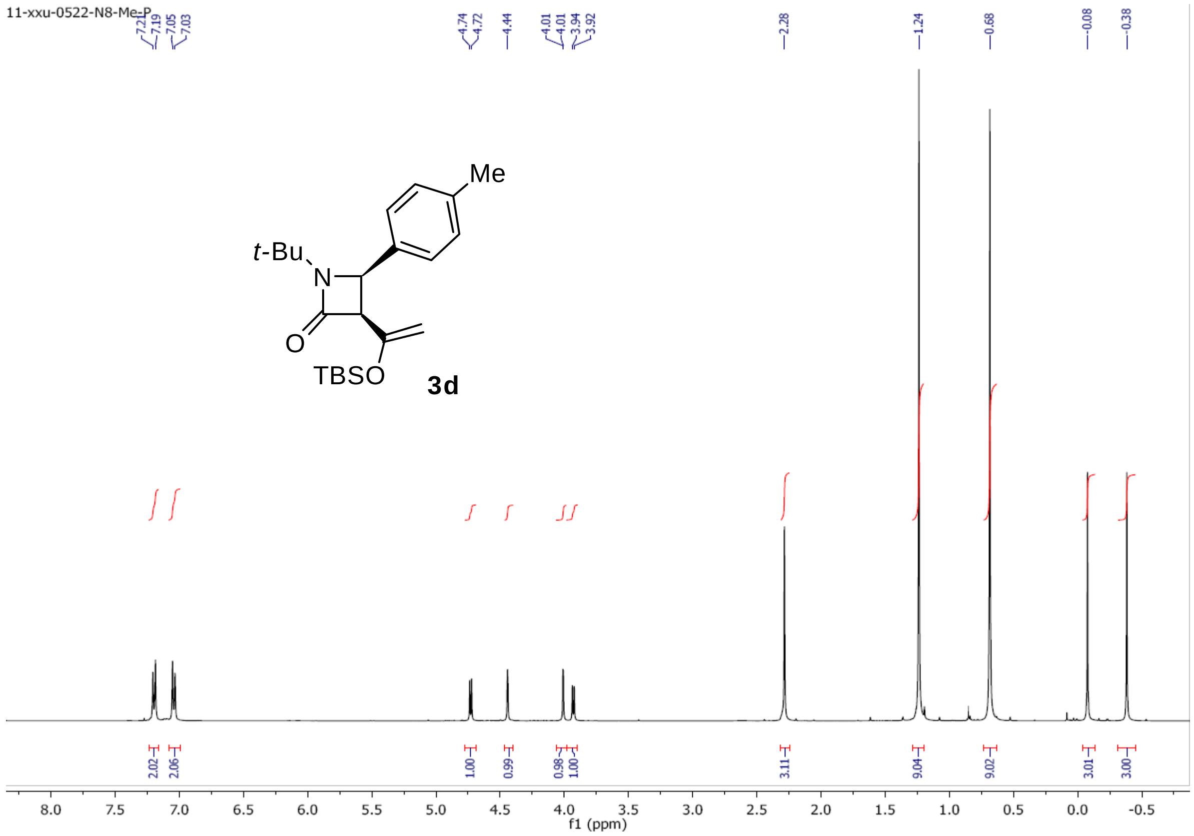
11-xxu-0522-N8-Me-P
7.21
7.19
7.05
7.03
4.74
4.72
4.44
4.01
4.01
3.94
3.92
2.28
1.24
0.68
0.08
-0.38
t-Bu
N
O
TBSO
Me
3d
2.02
2.06
1.00
0.99
0.98
1.00
3.11
9.04
9.02
3.01
3.00
f1 (ppm)

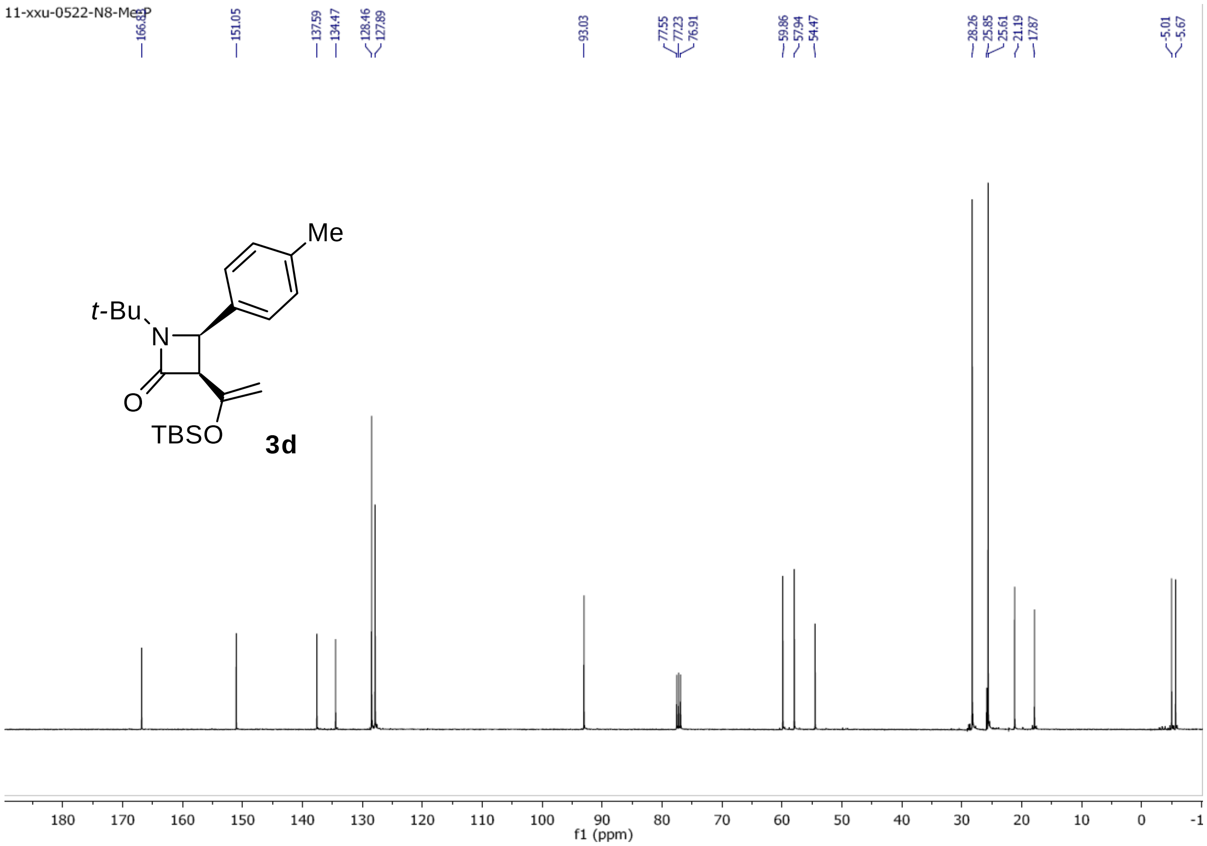
11-xxu-0522-N8-Me-P
166.83
151.05
137.59
134.47
128.46
127.89
93.03
77.55
77.23
76.91
59.86
57.94
54.47
28.26
25.85
25.61
21.19
17.87
-5.01
-5.67
t-Bu
N
O
TBSO
Me
3d
f1 (ppm)

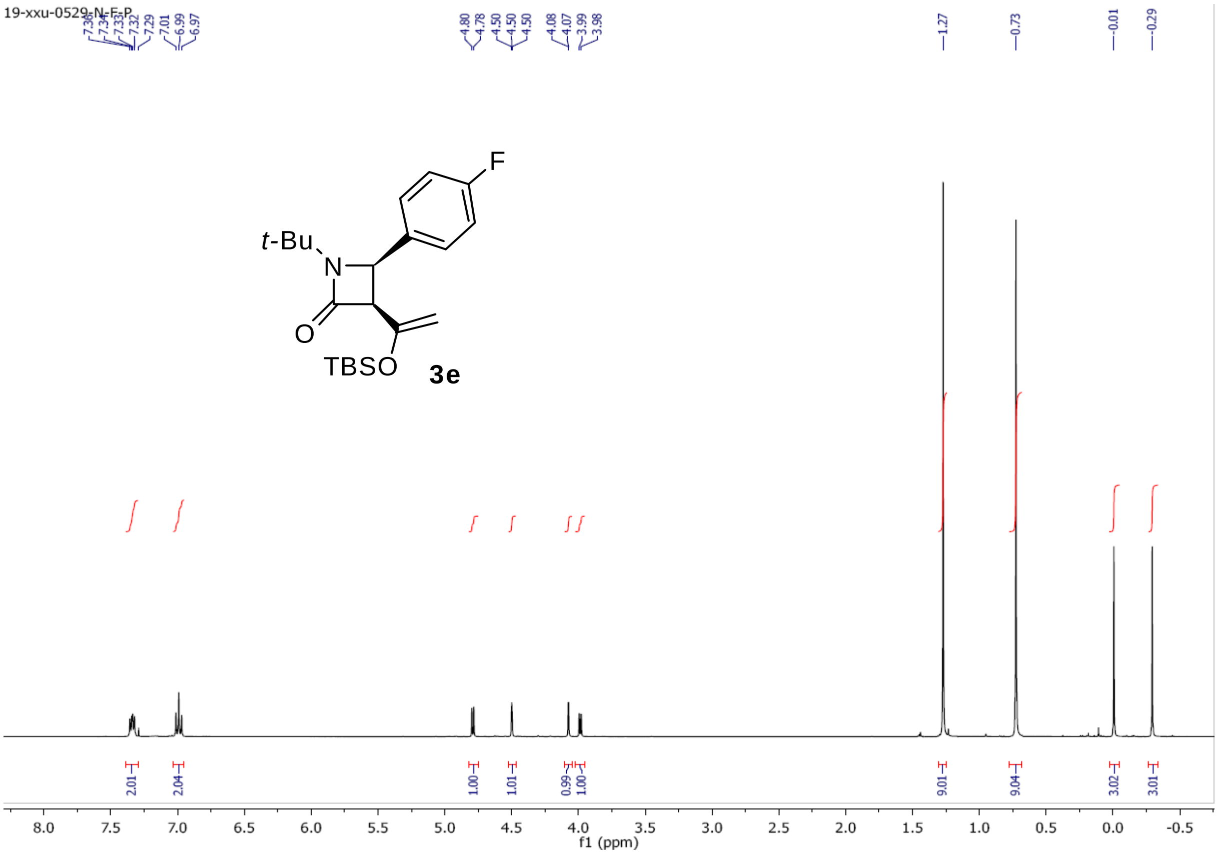
19-xxu-0529-N-F-P
7.36
7.34
7.33
7.32
7.29
7.01
6.99
6.97
4.80
4.78
4.50
4.50
4.50
4.50
4.08
4.07
3.99
3.98
1.27
0.73
-0.01
-0.29
t-Bu
N
O
TBSO
F
3e
2.01
2.04
1.00
1.01
0.99
1.00
9.01
9.04
3.02
3.01
f1 (ppm)

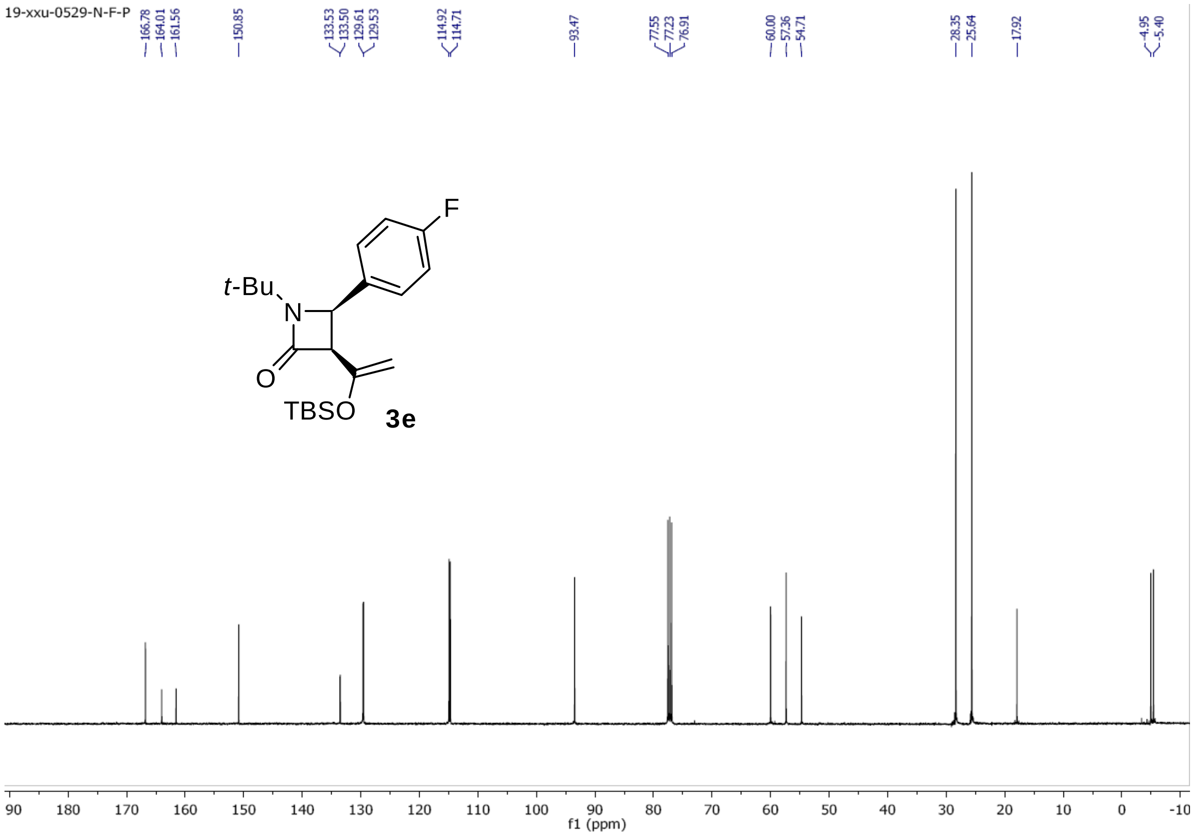
19-xxu-0529-N-F-P
166.78
164.01
161.56
150.85
133.53
133.50
129.61
129.53
114.92
114.71
93.47
77.55
77.23
76.91
60.00
57.36
54.71
28.35
25.64
17.92
-4.95
-5.40
t-Bu
N
O
TBSO
F
3e
f1 (ppm)

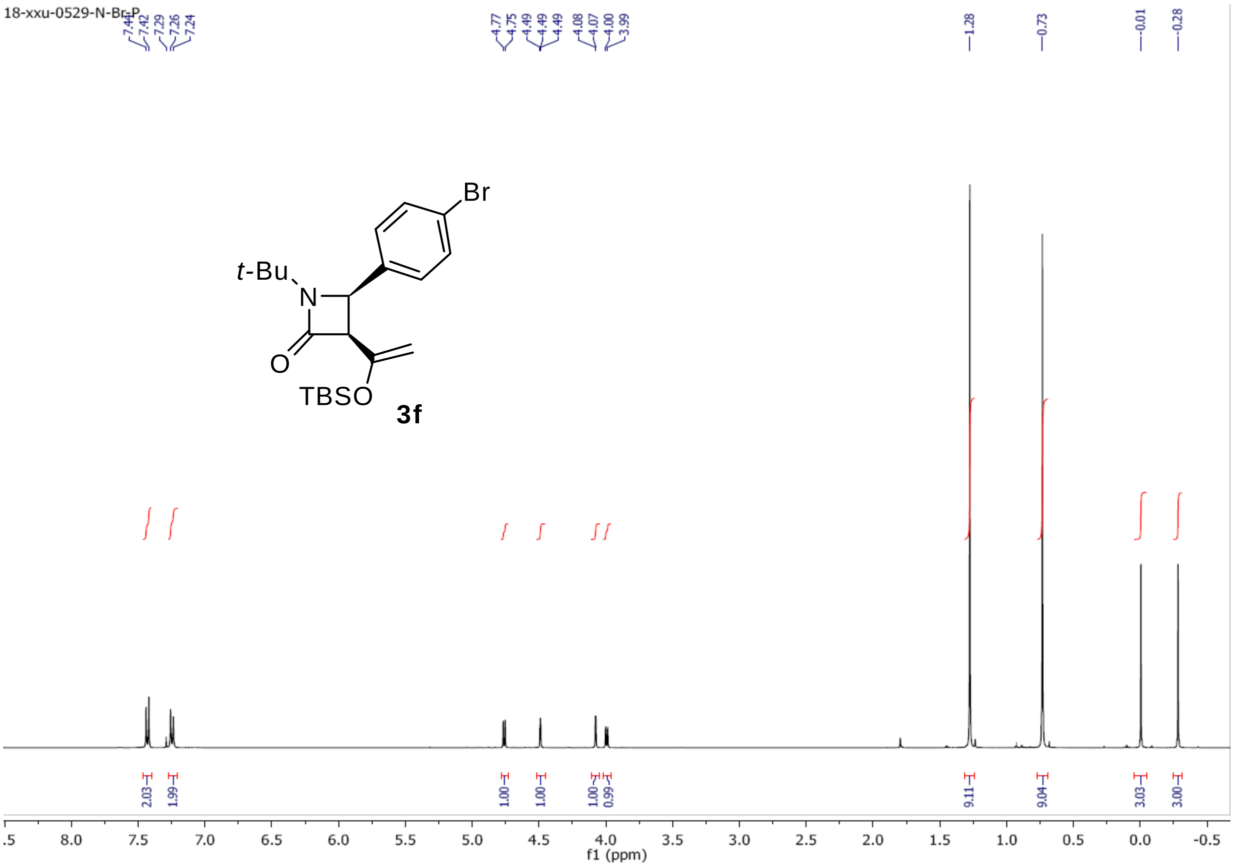
18-xxu-0529-N-Br-P
t-Bu
N
O
TBSO
Br
3f
f1 (ppm)

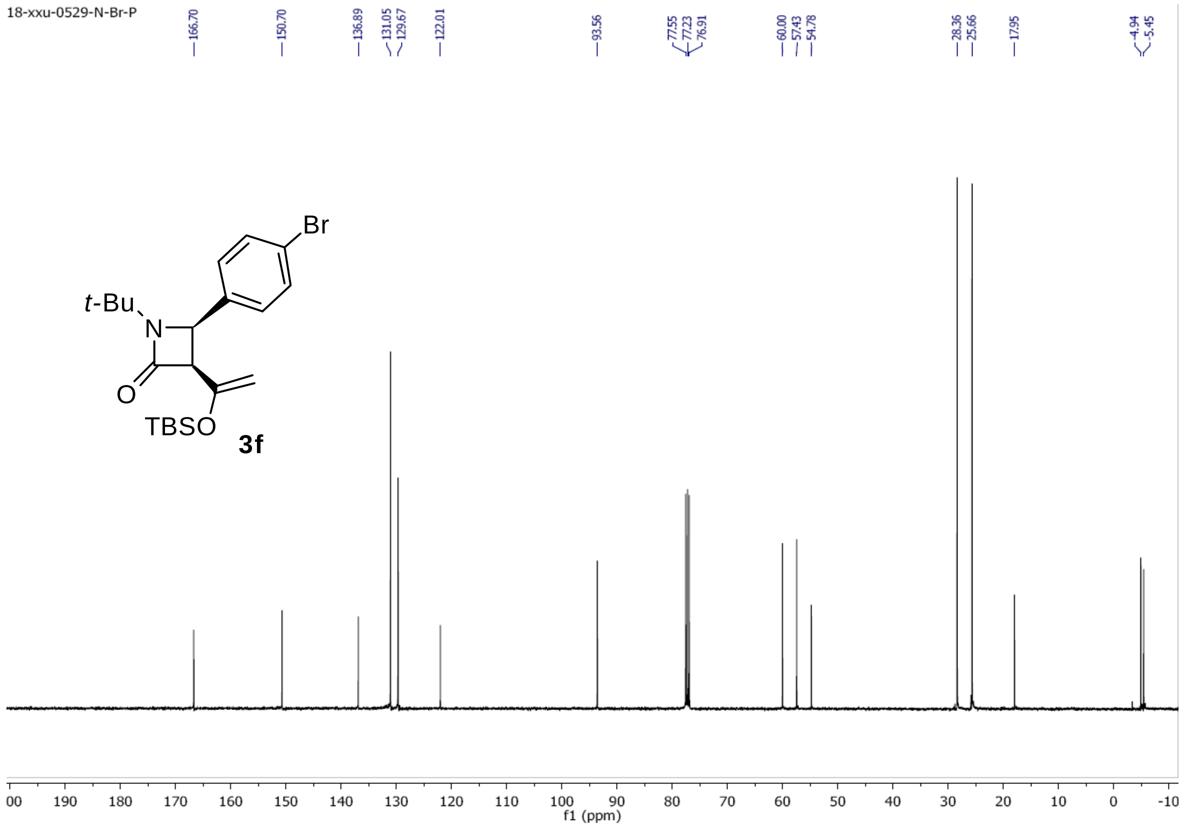
18-xxu-0529-N-Br-P
t-Bu
N
O
TBSO
Br
3f
f1 (ppm)

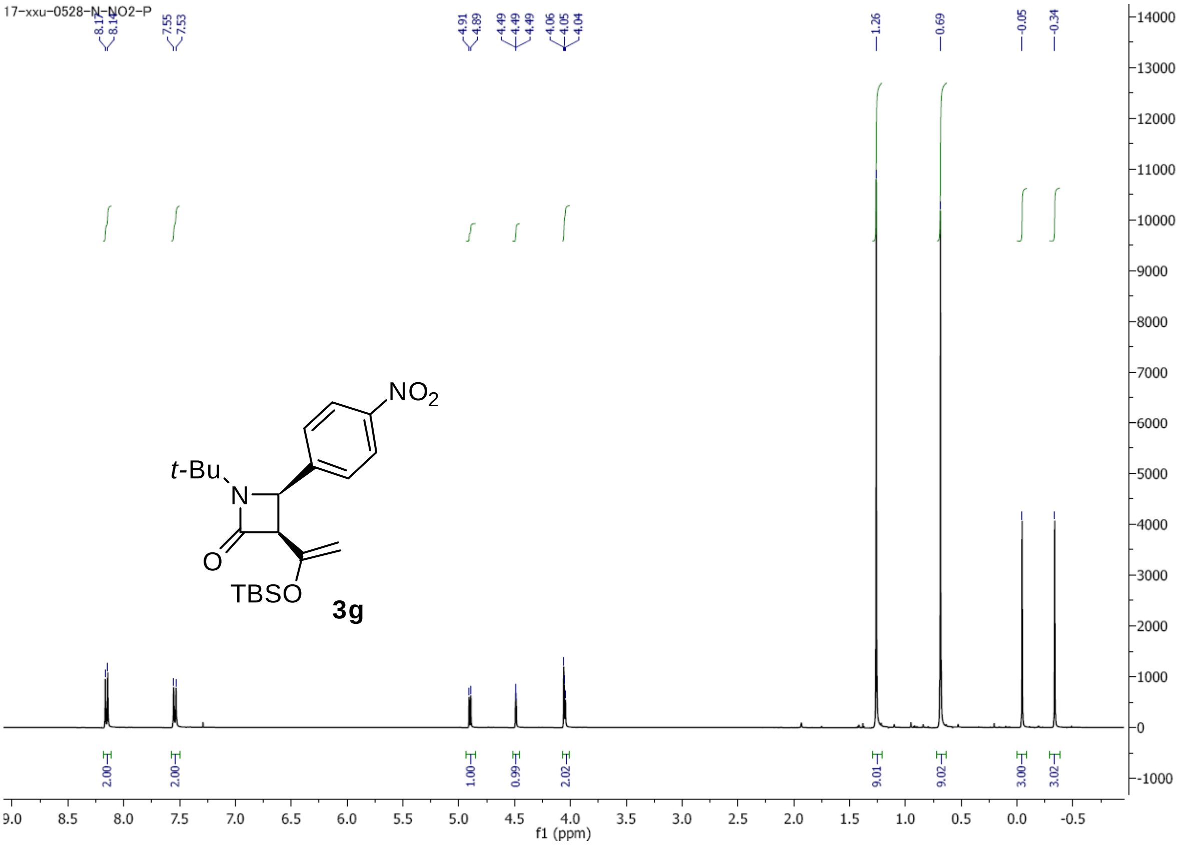
17-xxu-0528-N-NO2-P
8.17
8.14
7.55
7.53
4.91
4.89
4.49
4.49
4.49
4.06
4.05
4.04
1.26
0.69
-0.05
-0.34
NO2
t-Bu
N
O
TBSO
3g
2.00
2.00
1.00
0.99
2.02
9.01
9.02
3.00
3.02
f1 (ppm)

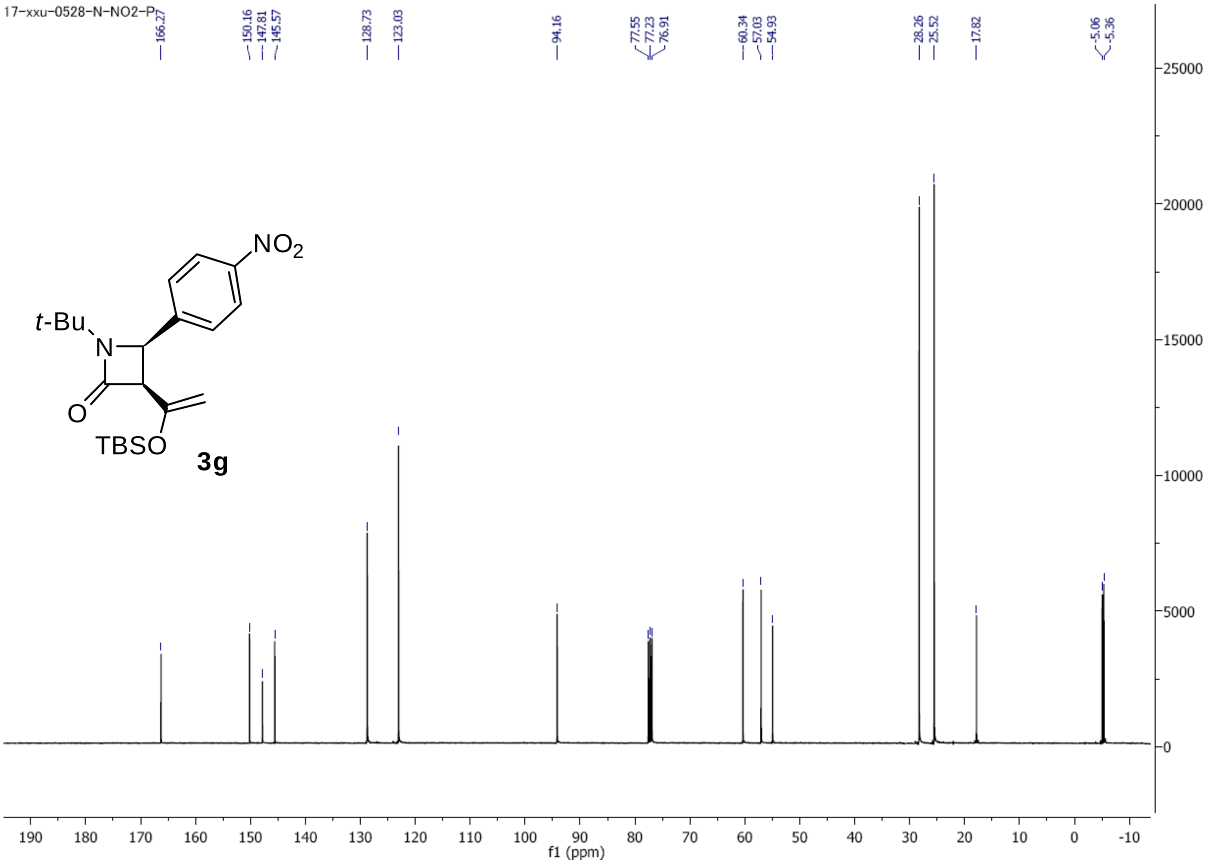
17-xxu-0528-N-NO2-P
166.27
150.16
147.81
145.57
128.73
123.03
94.16
77.55
77.23
76.91
60.34
57.03
54.93
28.26
25.52
17.82
-5.06
-5.36
NO2
t-Bu
N
O
TBSO
3g
f1 (ppm)

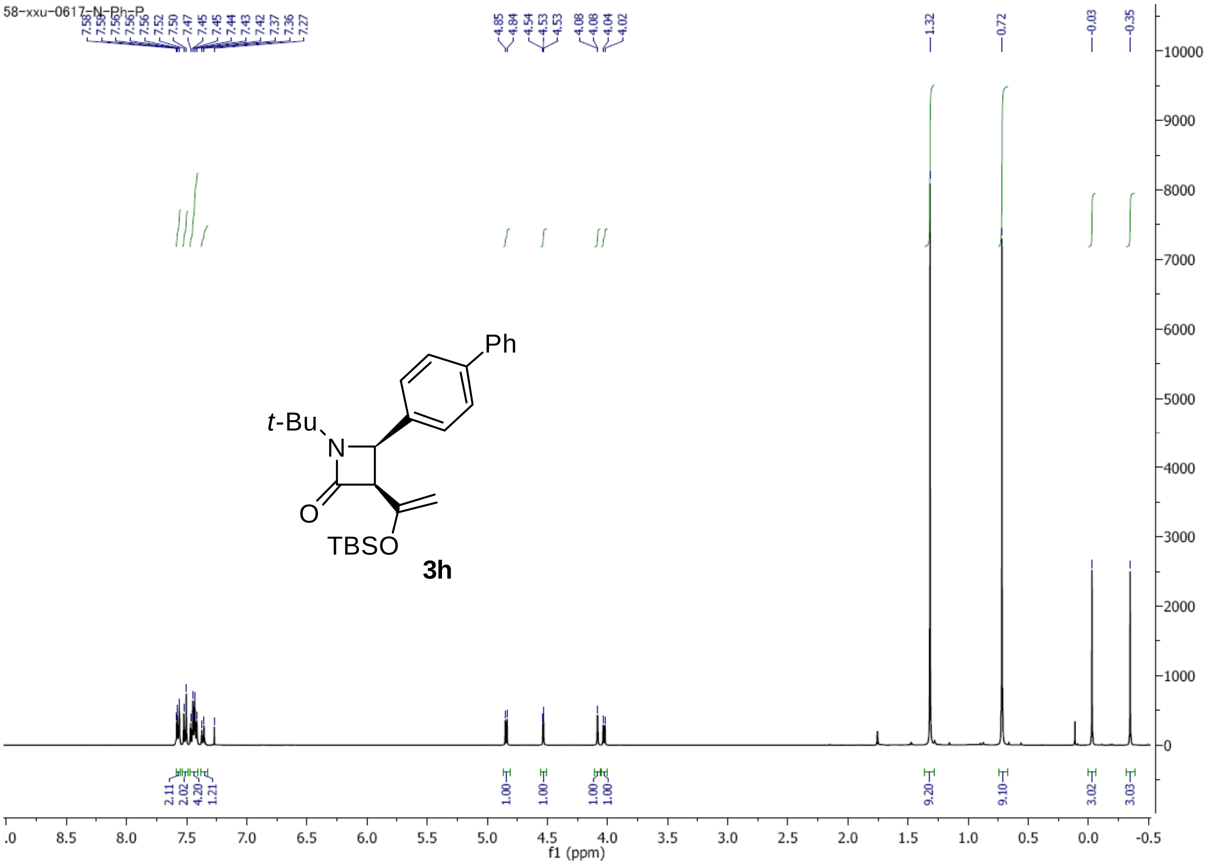
58-xxu-0617-N-Ph-P
t-Bu
N
O
TBSO
Ph
3h
f1 (ppm)

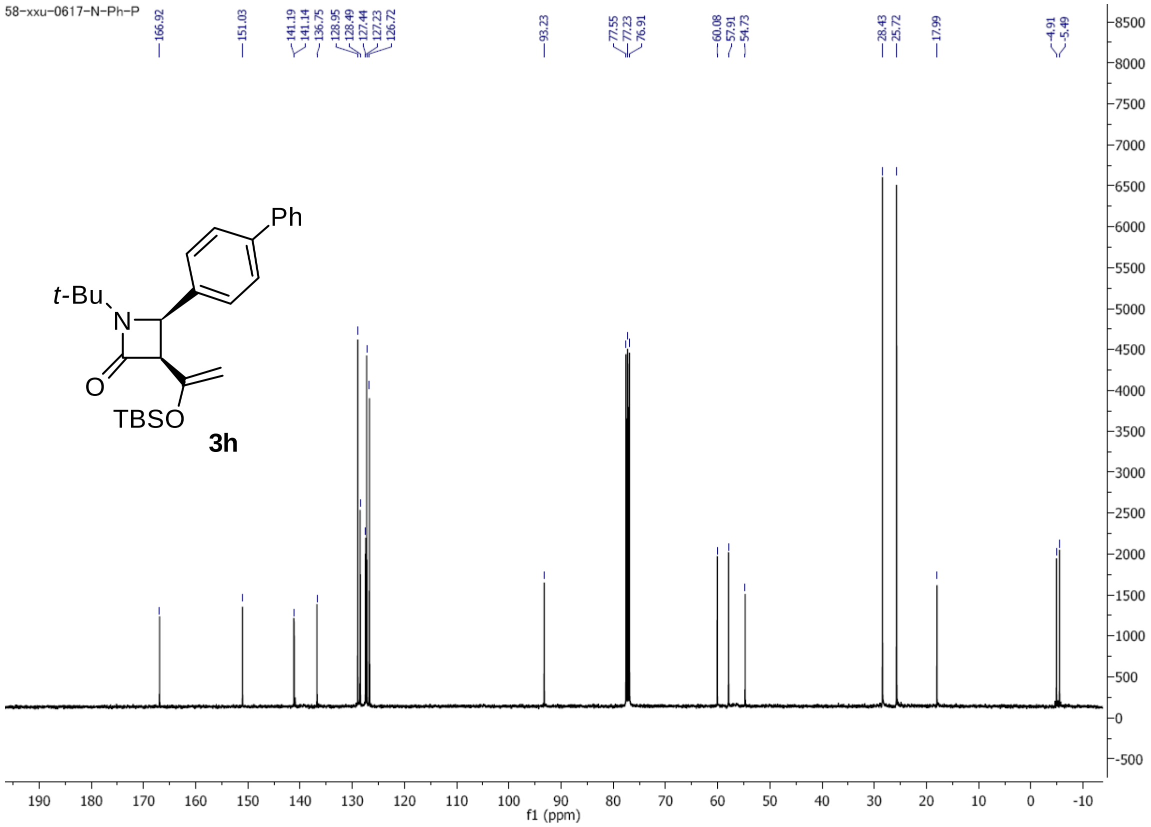
58-xxu-0617-N-Ph-P
t-Bu
N
O
TBSO
Ph
3h
f1 (ppm)

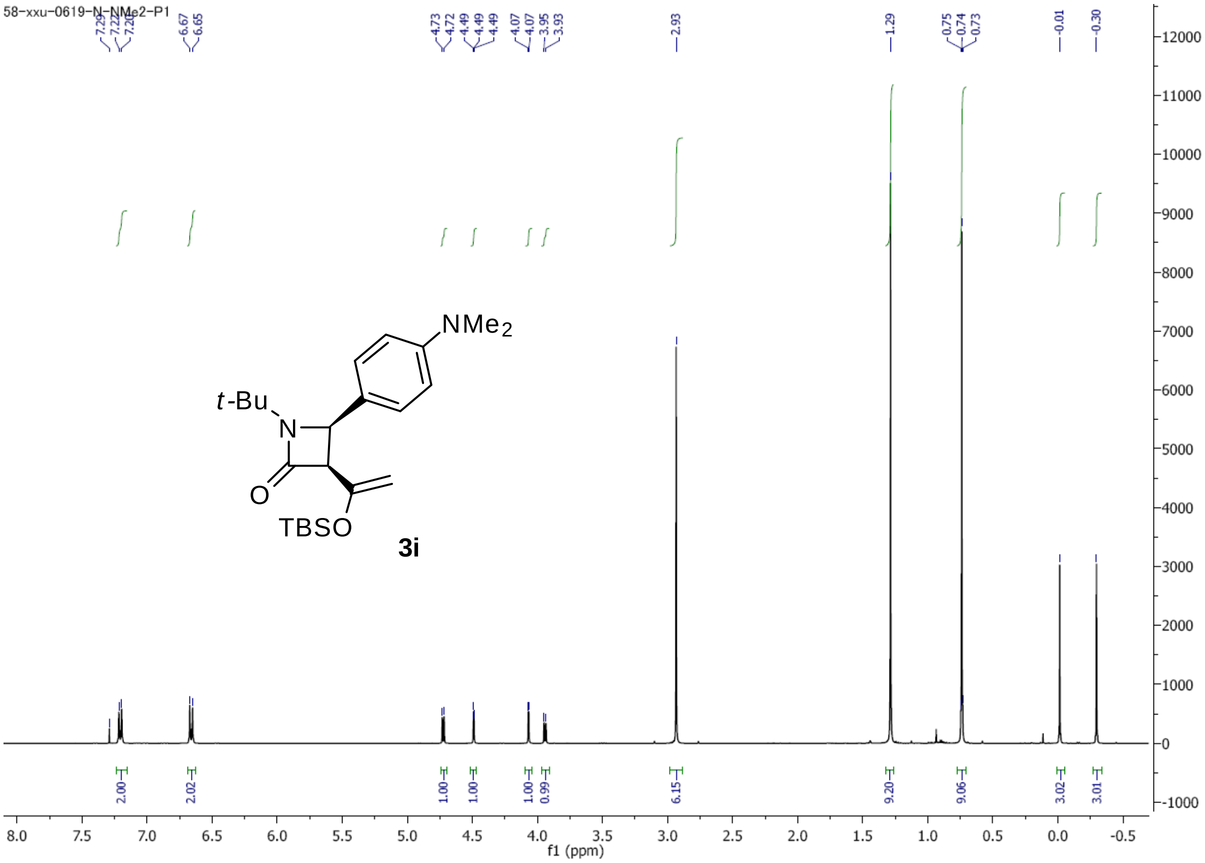
58-xxu-0619-N-NMe2-P1
7.29
7.22
7.20
6.67
6.65
4.73
4.72
4.49
4.49
4.49
4.07
4.07
3.95
3.93
2.93
1.29
0.75
0.74
0.73
-0.01
-0.30
NMe2
t-Bu
N
O
TBSO
3i
2.00
2.02
1.00
1.00
1.00
0.99
6.15
9.20
9.06
3.02
3.01
f1 (ppm)

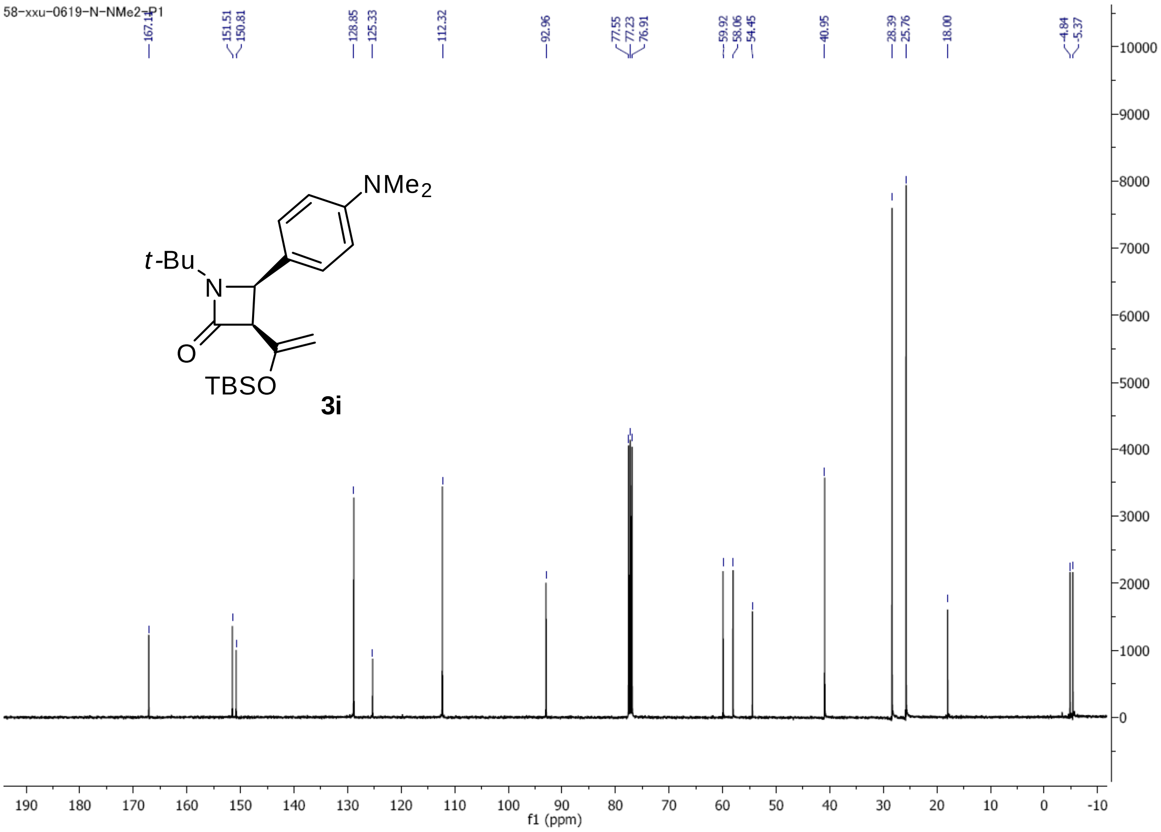
58-xxu-0619-N-NMe2-P1
167.11
151.51
150.81
128.85
125.33
112.32
92.96
77.55
77.23
76.91
59.92
58.06
54.45
40.95
28.39
25.76
18.00
-4.84
-5.37
NMe2
t-Bu
N
O
TBSO
3i
f1 (ppm)

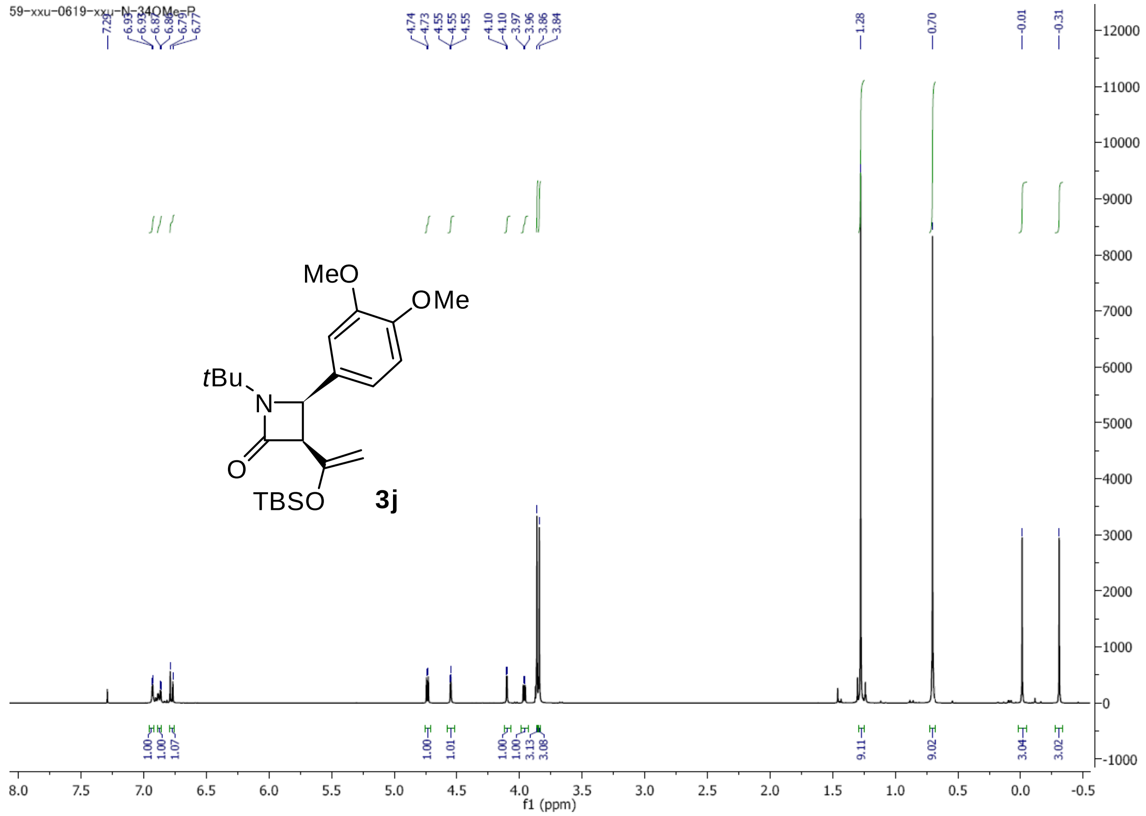
59-xxu-0619-xxu-N-34OMe-P
7.29
6.93
6.93
6.87
6.86
6.79
6.77
4.74
4.73
4.55
4.55
4.55
4.10
4.10
3.97
3.96
3.86
3.84
1.28
0.70
-0.01
-0.31
MeO
OMe
tBu
N
O
TBSO
3j
1.00
1.00
1.07
1.00
1.01
1.00
1.00
3.13
3.08
9.11
9.02
3.04
3.02
f1 (ppm)

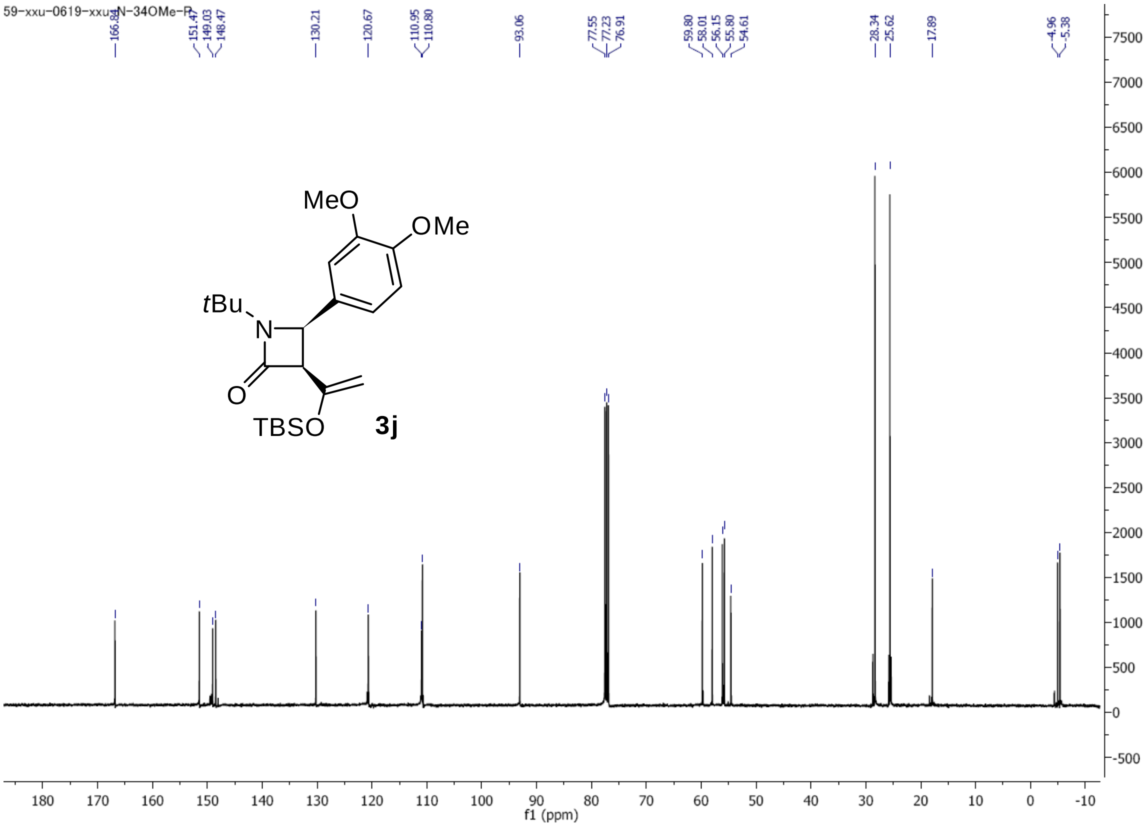
59-xxu-0619-xxu-N-34OMe-P
166.84
151.47
149.03
148.47
130.21
120.67
110.95
110.80
93.06
77.55
77.23
76.91
59.80
58.01
56.15
55.80
54.61
28.34
25.62
17.89
-4.96
-5.38
MeO
OMe
tBu
N
O
TBSO
3j
f1 (ppm)

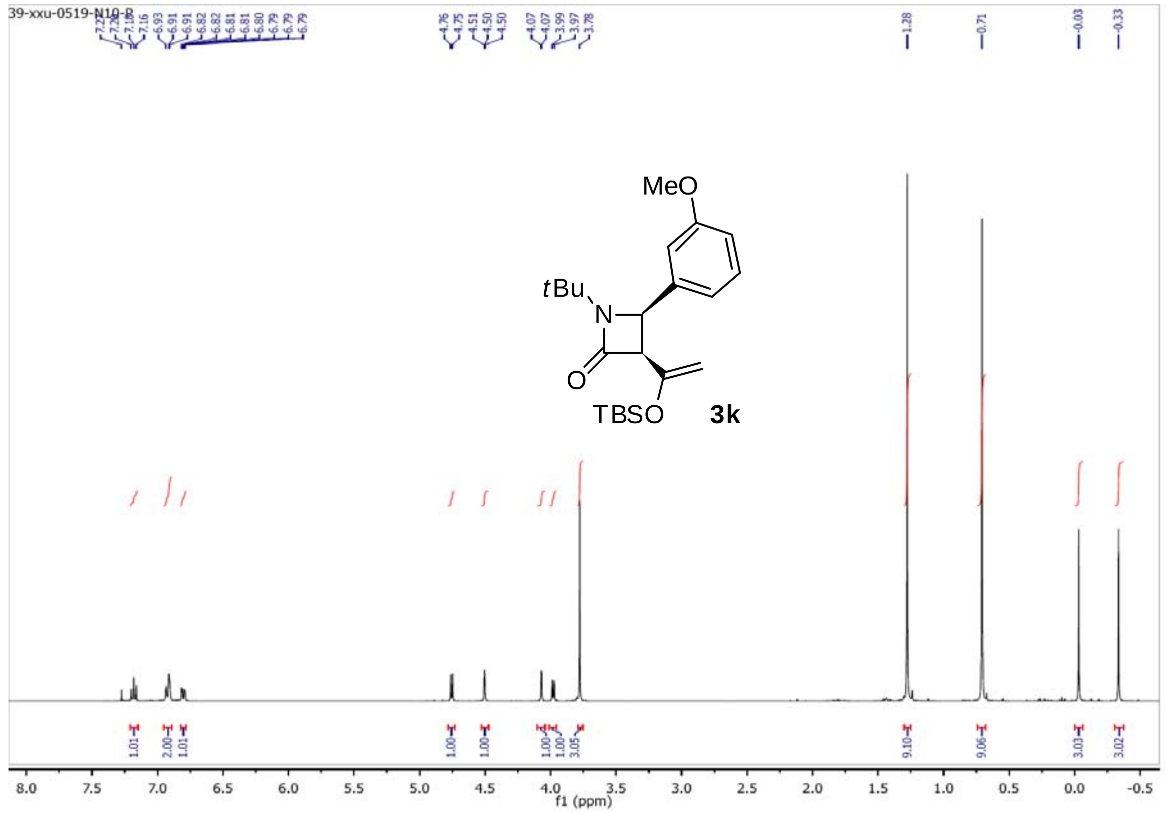
39-xxu-0519-N10-P
7.22
7.20
7.18
7.16
6.93
6.91
6.91
6.82
6.82
6.81
6.81
6.80
6.79
6.79
6.79
4.76
4.75
4.51
4.50
4.50
4.07
4.07
3.99
3.97
3.78
1.28
0.71
-0.03
-0.33
MeO
tBu
N
O
TBSO
3k
1.01
2.00
1.01
1.00
1.00
1.00
1.00
3.05
9.10
9.06
3.03
3.02
8.0
7.5
7.0
6.5
6.0
5.5
5.0
4.5
4.0
3.5
3.0
2.5
2.0
1.5
1.0
0.5
0.0
-0.5
f1 (ppm)

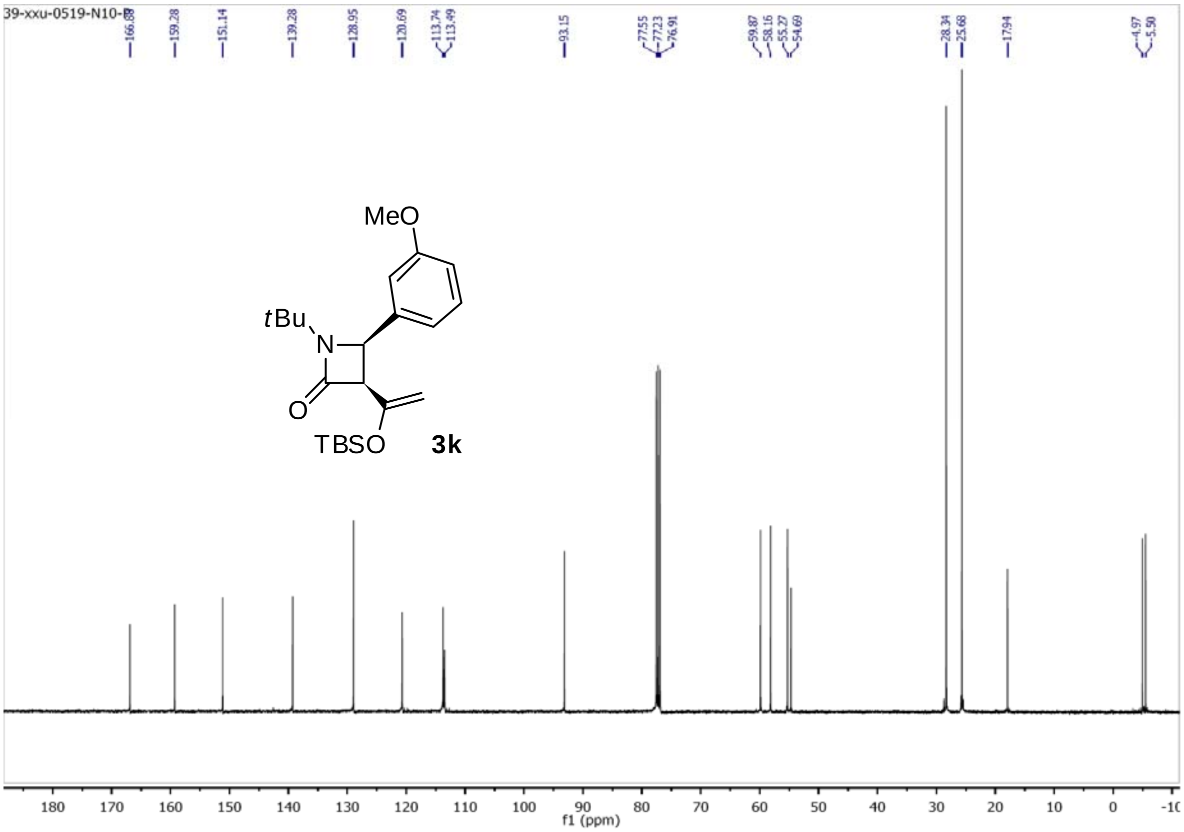
39-xxu-0519-N10-C
166.88
159.28
151.14
139.28
128.95
120.69
113.74
113.49
93.15
77.55
77.23
76.91
59.87
58.16
55.27
54.69
28.34
25.68
17.94
-4.97
-5.50
MeO
tBu
N
O
TBSO
3k
180
170
160
150
140
130
120
110
100
90
80
70
60
50
40
30
20
10
0
-10
f1 (ppm)

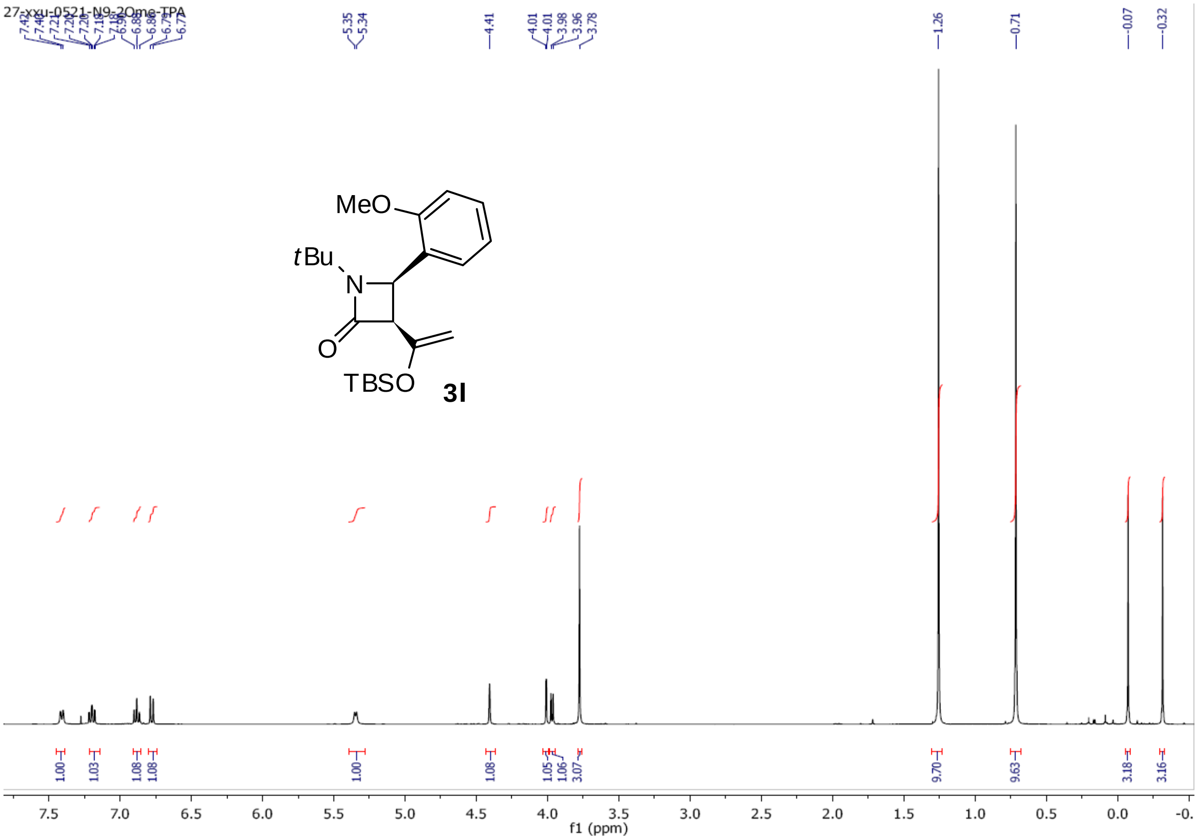
27-xxu-0521-N9-2Ome-TPA
MeO
tBu
N
O
TBSO
3l
f1 (ppm)

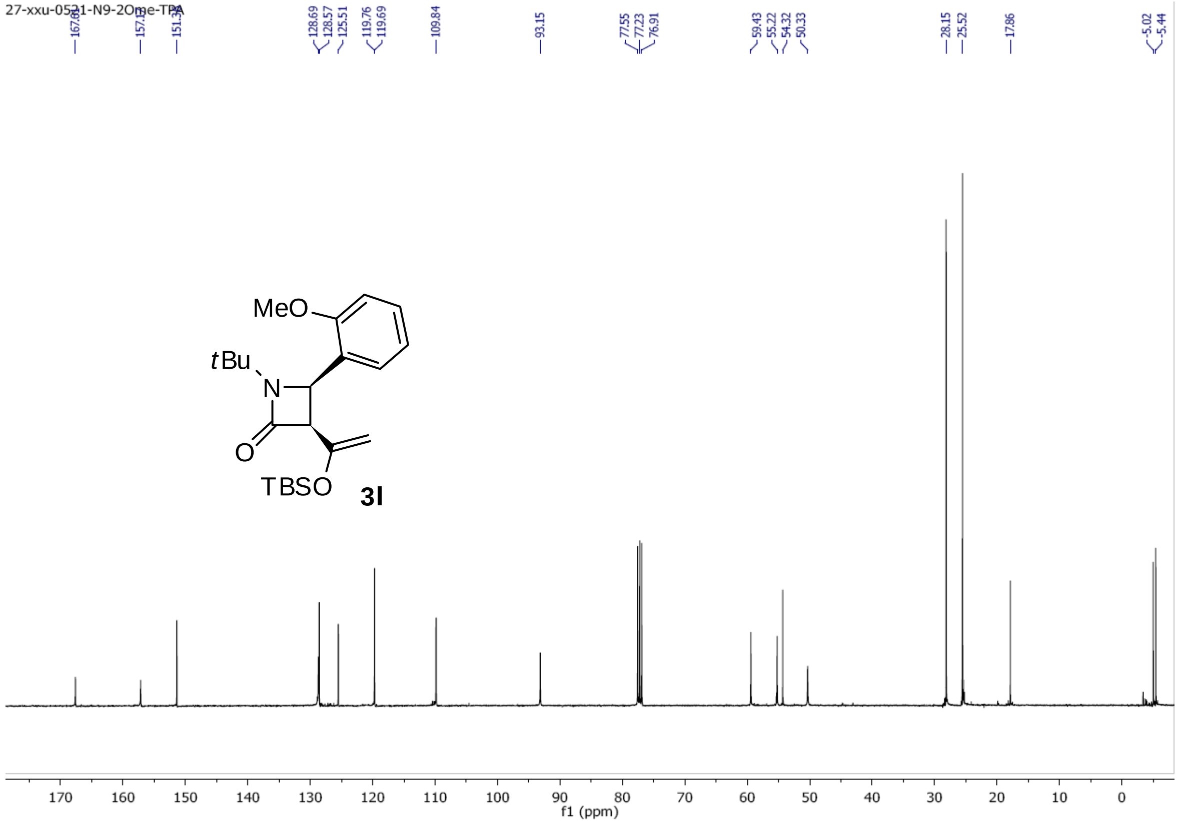
27-xxu-0521-N9-2Ome-TPA
MeO
tBu
N
O
TBSO
3l
f1 (ppm)

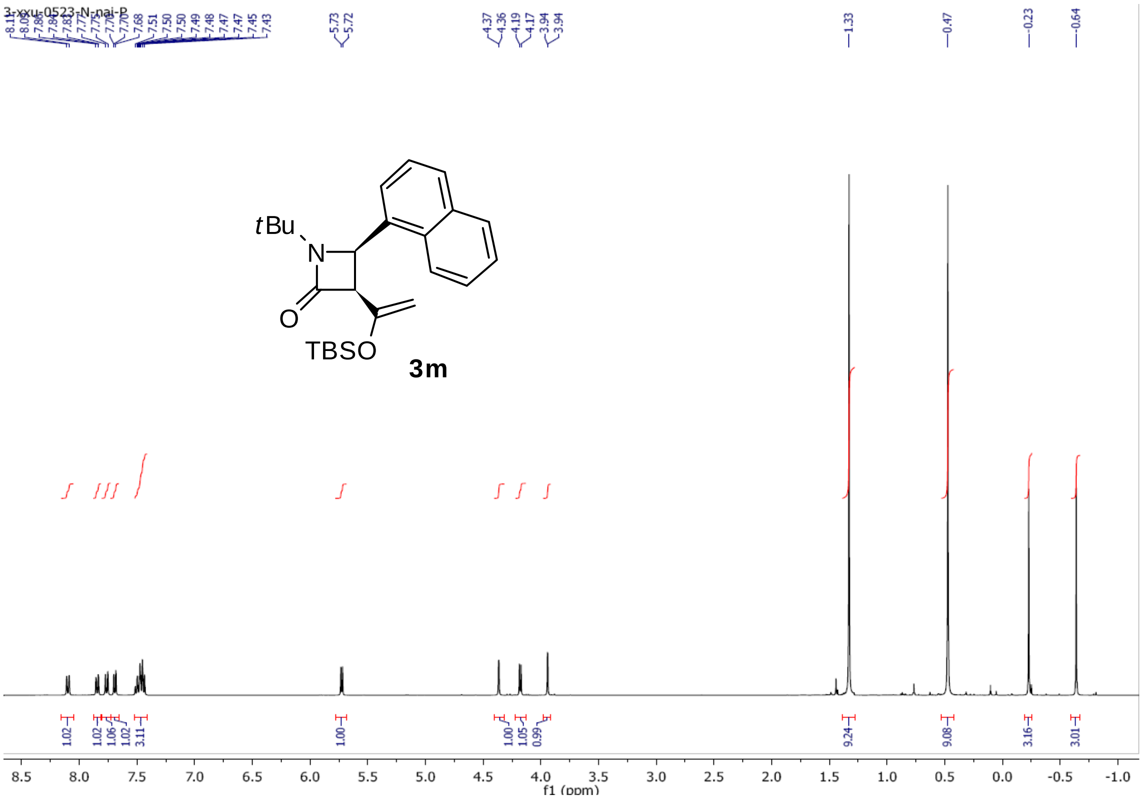
3-xxu-0523-N-nai-P
tBu
N
O
TBSO
3m
f1 (ppm)

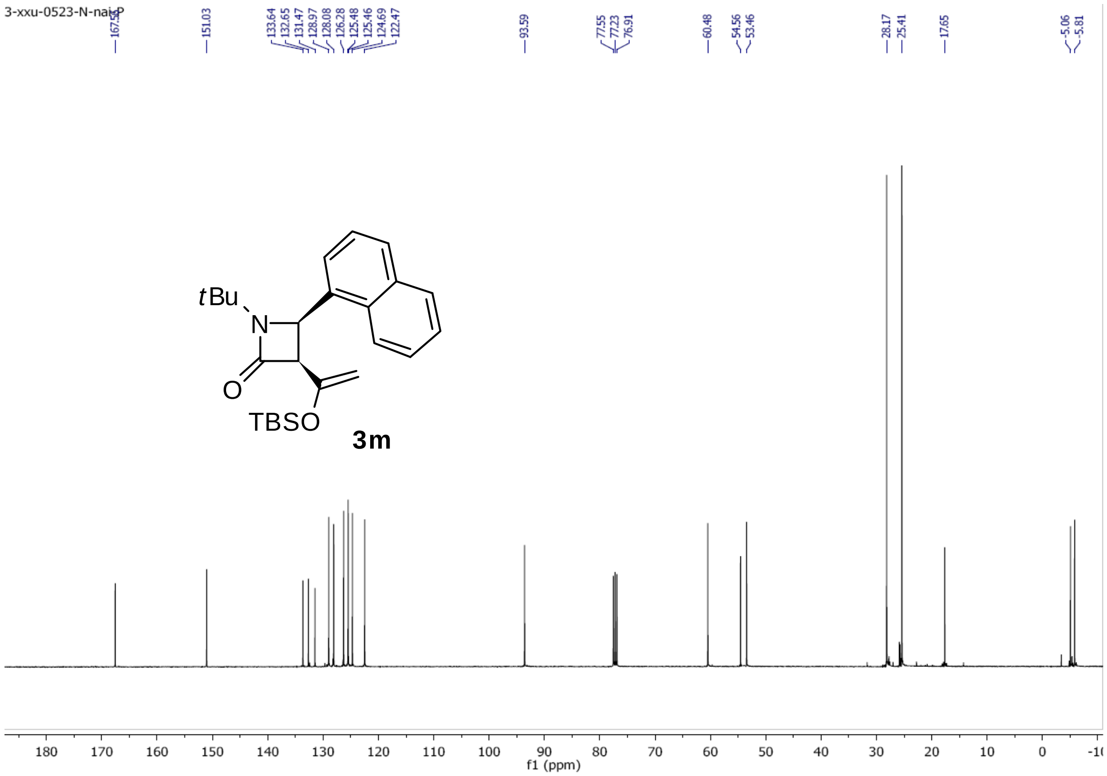
3-xxu-0523-N-nai-P
tBu
N
O
TBSO
3m
f1 (ppm)

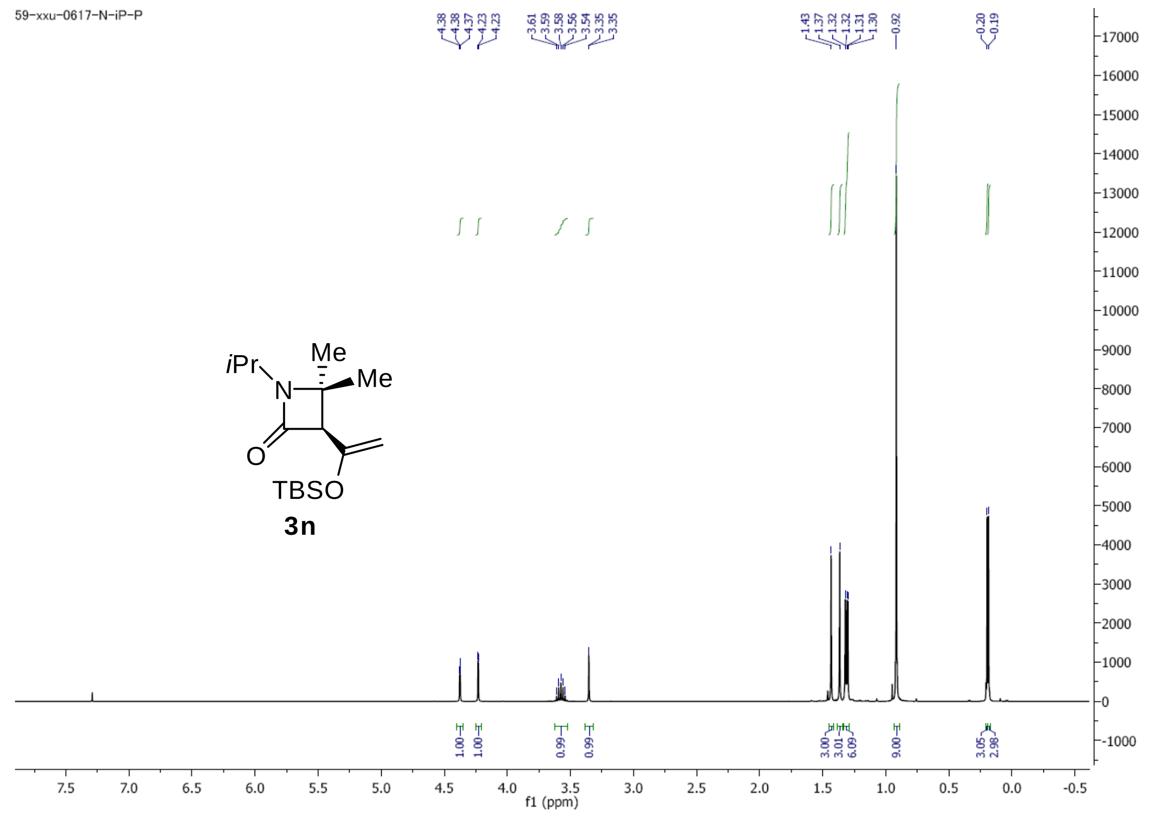
59-xxu-0617-N-iP-P
4.38 4.38 4.37 4.23 4.23
3.61 3.59 3.58 3.56 3.54 3.35 3.35
1.43 1.37 1.32 1.32 1.31 1.30
0.92
0.20 0.19
1.00 1.00 0.99 0.99 3.00 3.01 6.09 9.00 3.05 2.98
iPr
N
Me
Me
O
TBSO
3n
f1 (ppm)

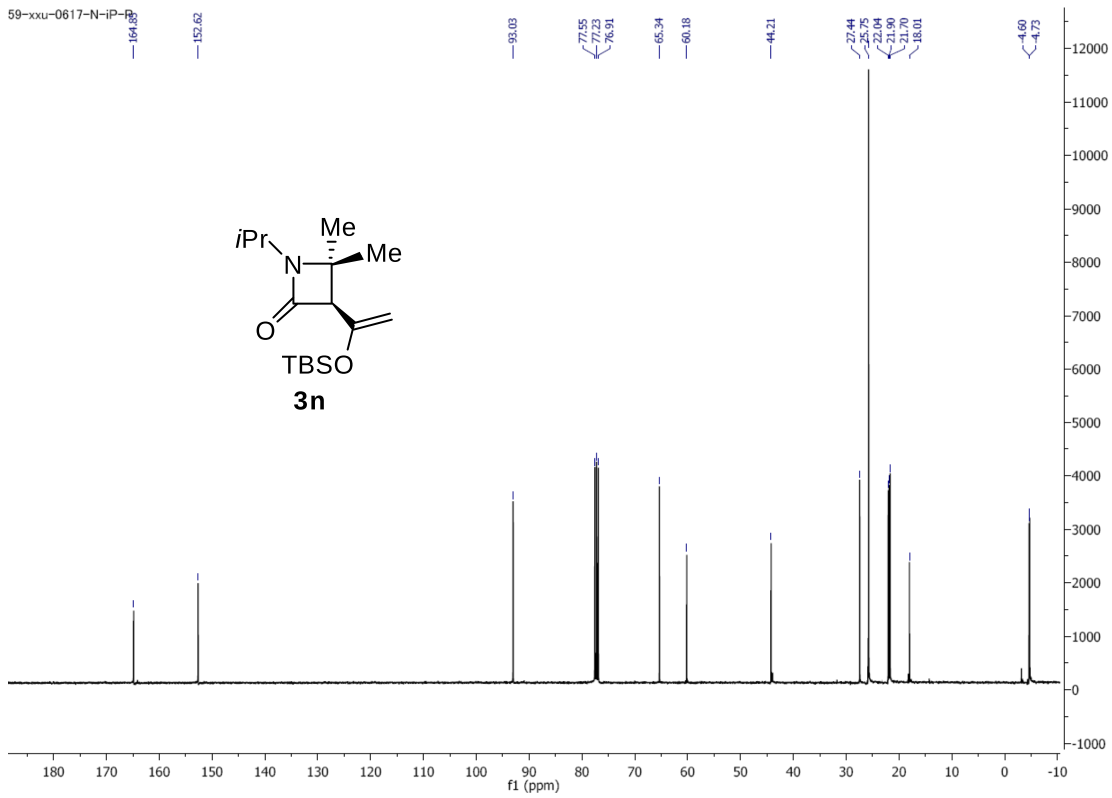
59-xxu-0617-N-iP-P
164.85
152.62
93.03
77.55 77.23 76.91
65.34
60.18
44.21
27.44 25.75 22.04 21.90 21.70 18.01
-4.60 -4.73
iPr
N
Me
Me
O
TBSO
3n
f1 (ppm)

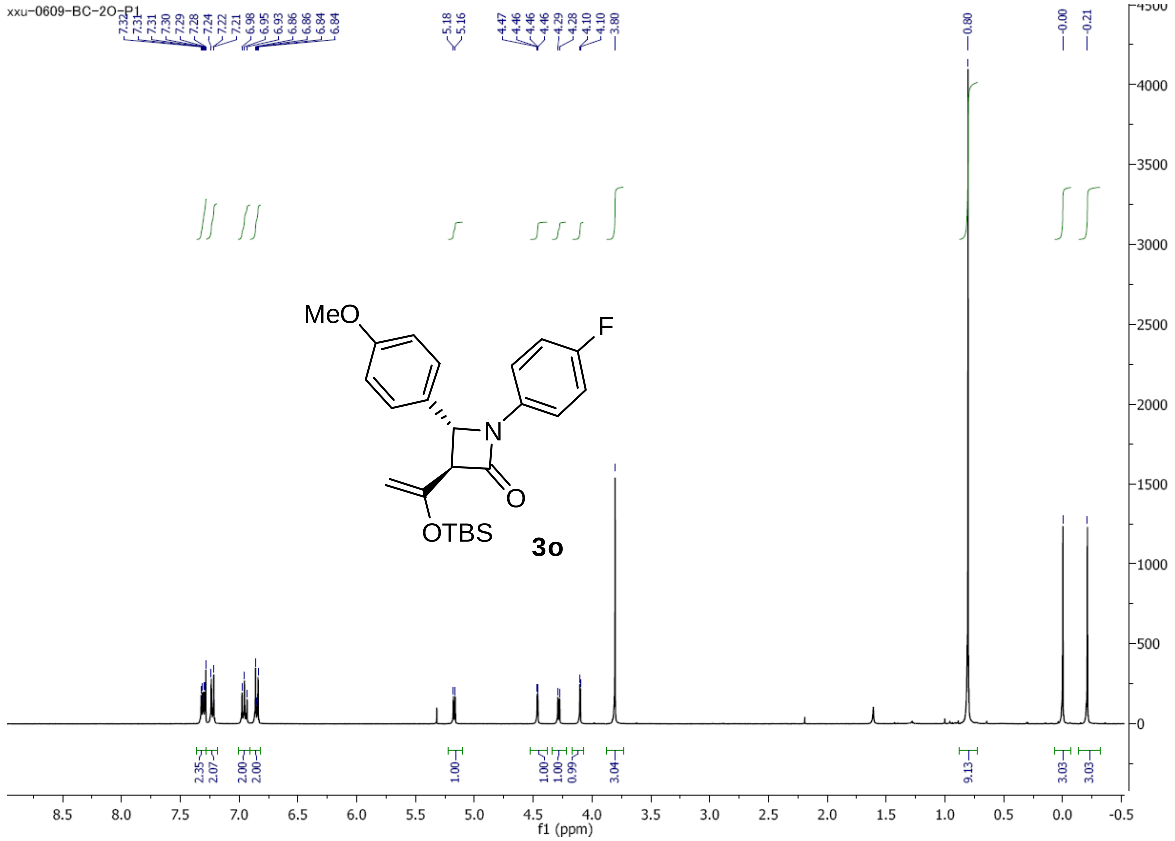

3o

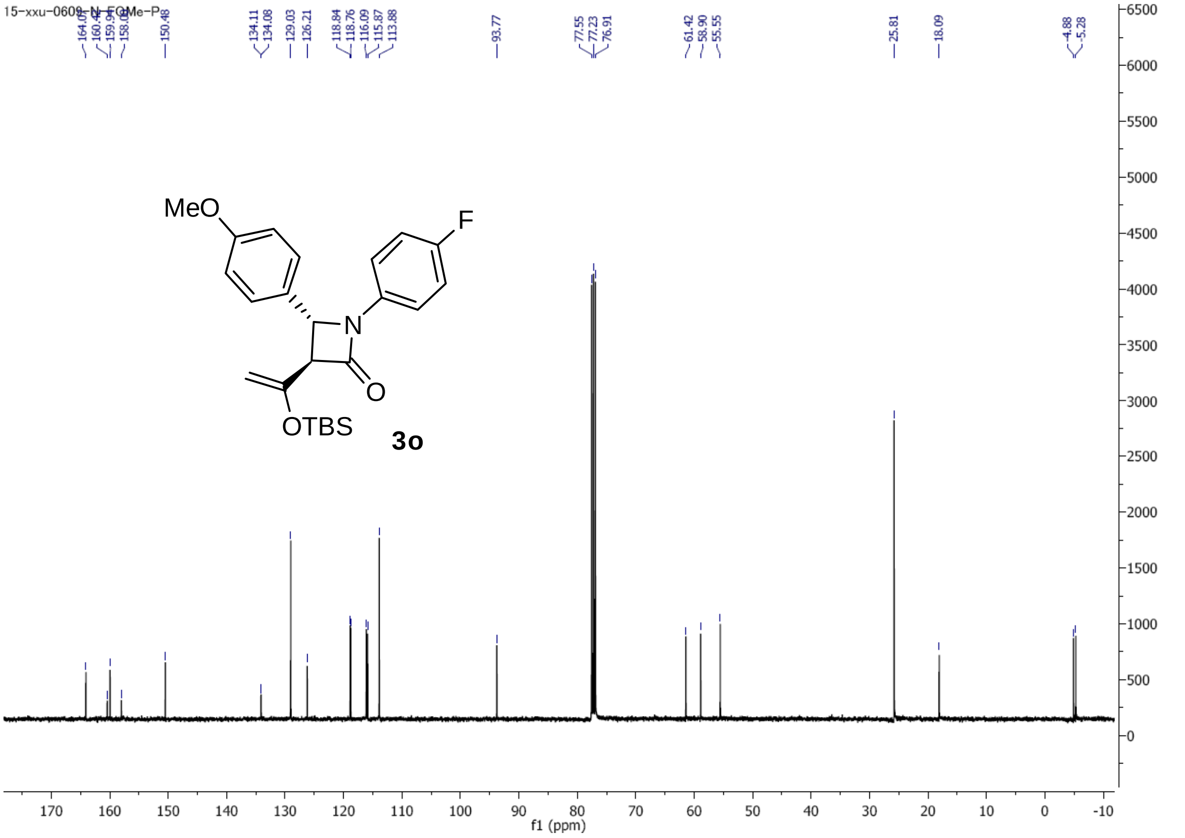

3o

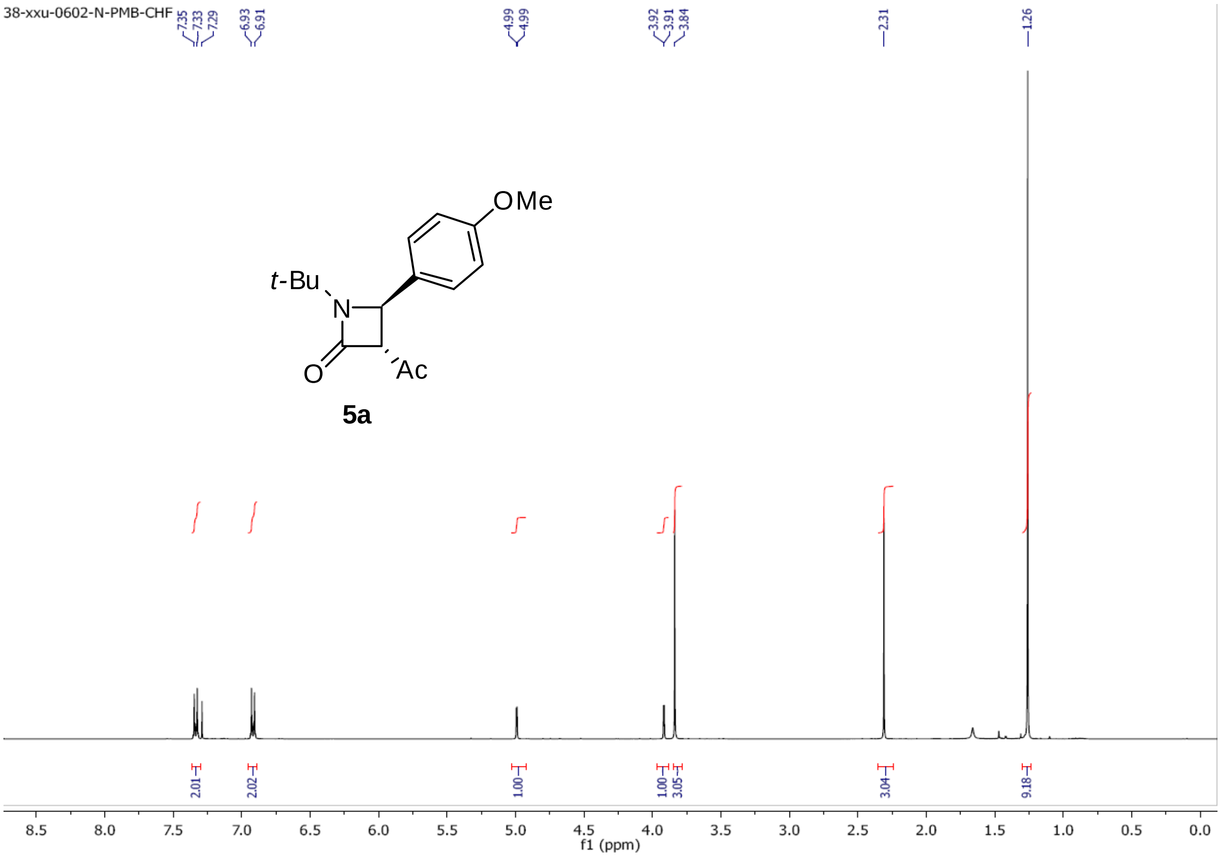
38-xxu-0602-N-PMB-CHF
7.35
7.33
7.29
6.93
6.91
4.99
4.99
3.92
3.91
3.84
2.31
1.26
OMe
t-Bu
N
O
Ac
5a
2.01
2.02
1.00
1.00
3.05
3.04
9.18
8.5 8.0 7.5 7.0 6.5 6.0 5.5 5.0 4.5 4.0 3.5 3.0 2.5 2.0 1.5 1.0 0.5 0.0
f1 (ppm)

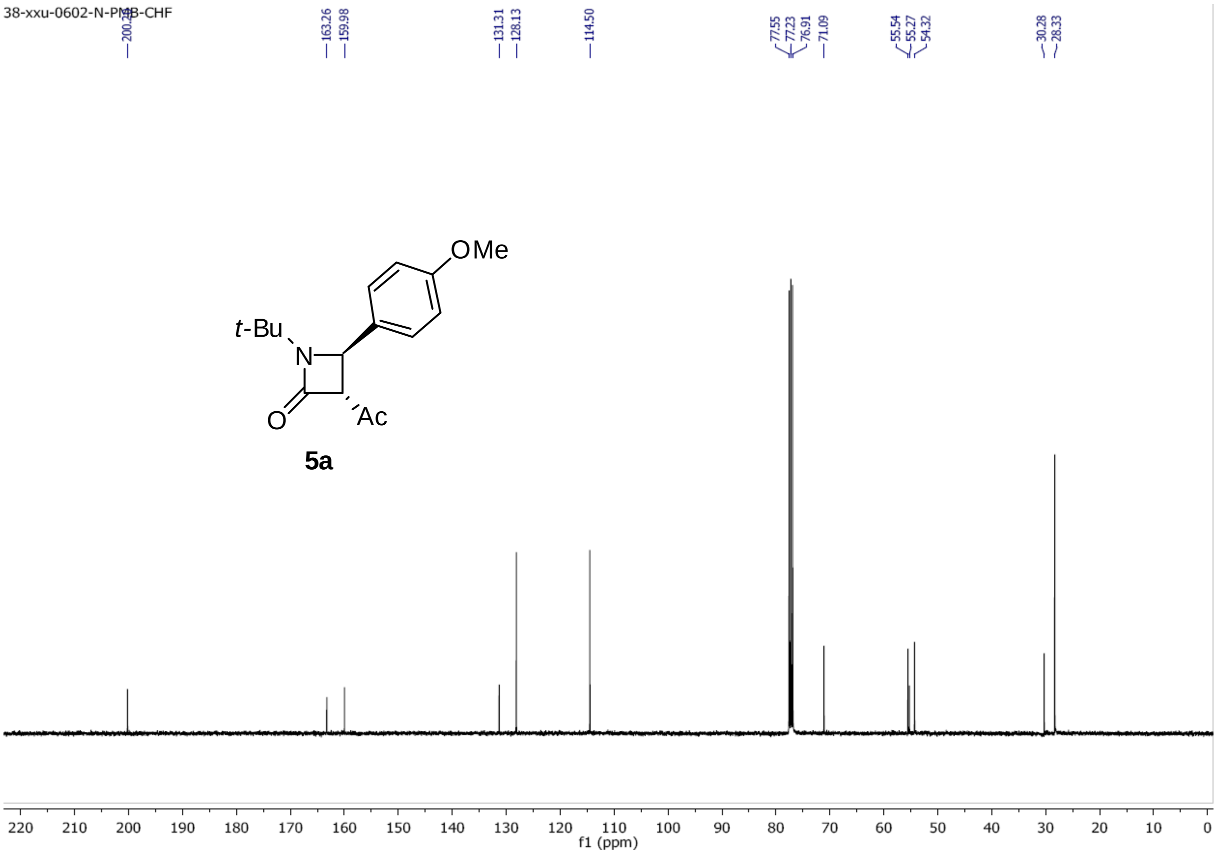
38-xxu-0602-N-PMB-CHF
163.26
159.98
131.31
128.13
114.50
77.55
77.23
76.91
71.09
55.54
55.27
54.32
30.28
28.33
OMe
t-Bu
N
O
Ac
5a
220 210 200 190 180 170 160 150 140 130 120 110 100 90 80 70 60 50 40 30 20 10 0
f1 (ppm)

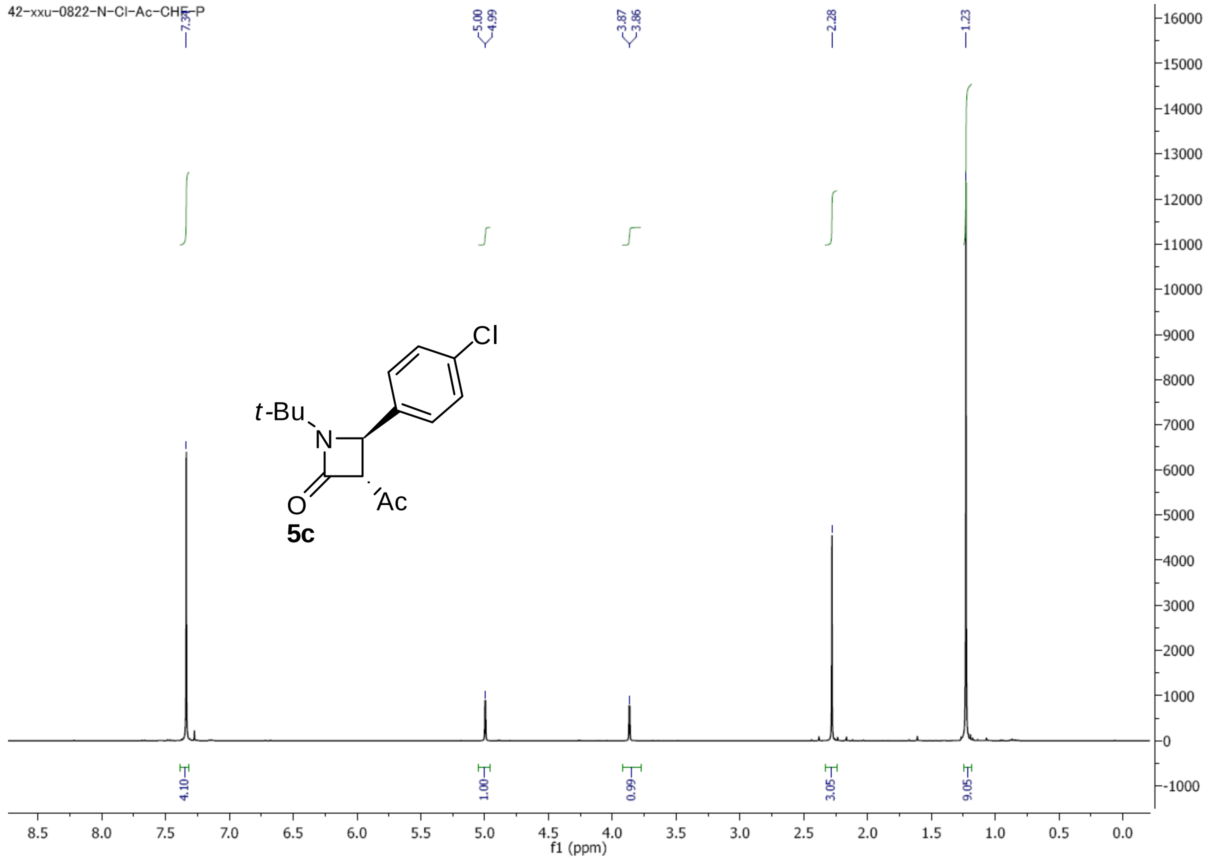
42-xxu-0822-N-Cl-Ac-CHF-P
7.34
5.00
4.99
3.87
3.86
2.28
1.23
4.10
1.00
0.99
3.05
9.05
t-Bu
N
Cl
O
Ac
5c
f1 (ppm)

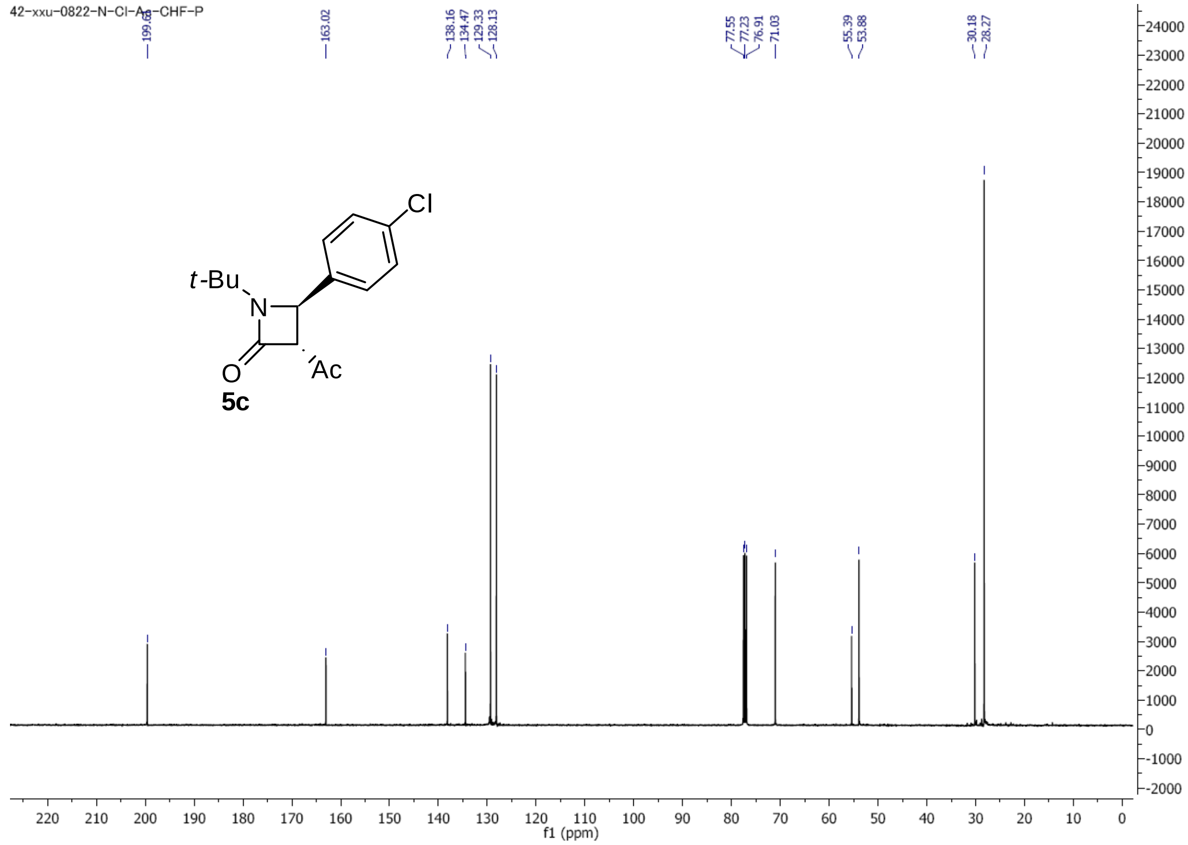
42-xxu-0822-N-Cl-Ac-CHF-P
199.63
163.02
138.16
134.47
129.33
128.13
77.55
77.23
76.91
71.03
55.39
53.88
30.18
28.27
t-Bu
N
Cl
O
Ac
5c
f1 (ppm)

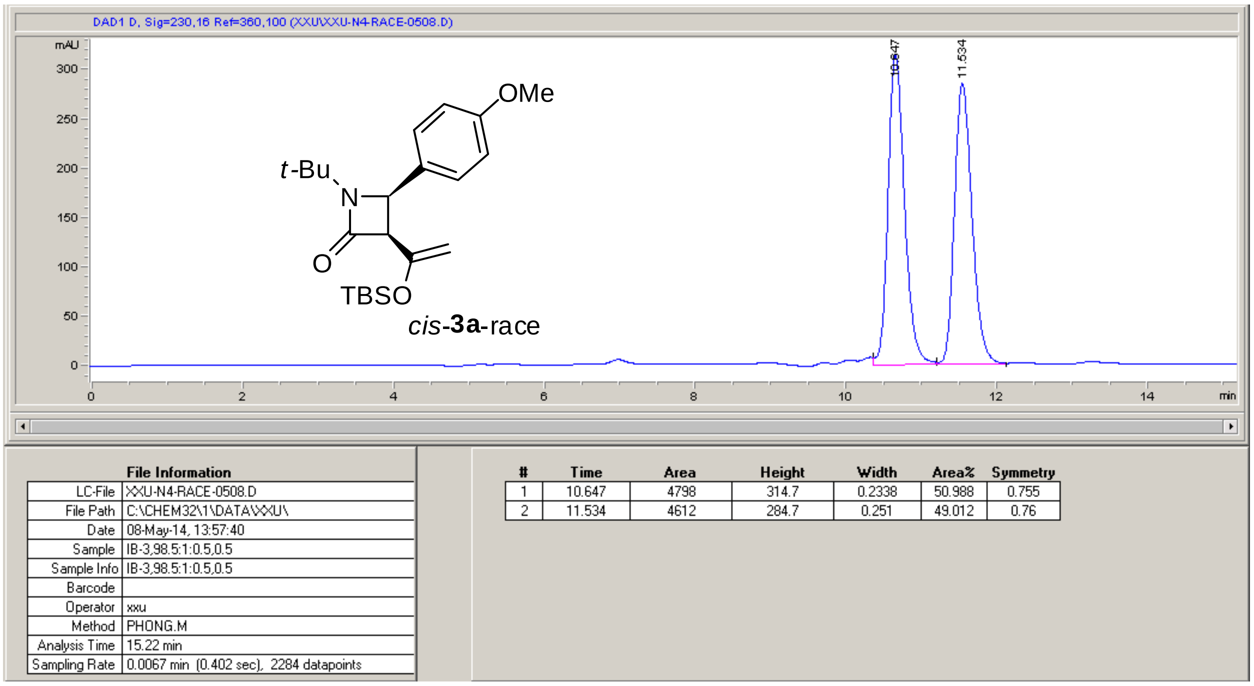

File Information	
LC-File	XXU-N4-RACE-0508.D
File Path	C:\CHEM32\1\DATA\XXU\
Date	08-May-14, 13:57:40
Sample	IB-3,98.5:1:0.5,0.5
Sample Info	IB-3,98.5:1:0.5,0.5
Barcode	
Operator	xxu
Method	PHONG.M
Analysis Time	15.22 min
Sampling Rate	0.0067 min (0.402 sec), 2284 datapoints

#	Time	Area	Height	Width	Area%	Symmetry
1	10.647	4798	314.7	0.2338	50.988	0.755
2	11.534	4612	284.7	0.251	49.012	0.76

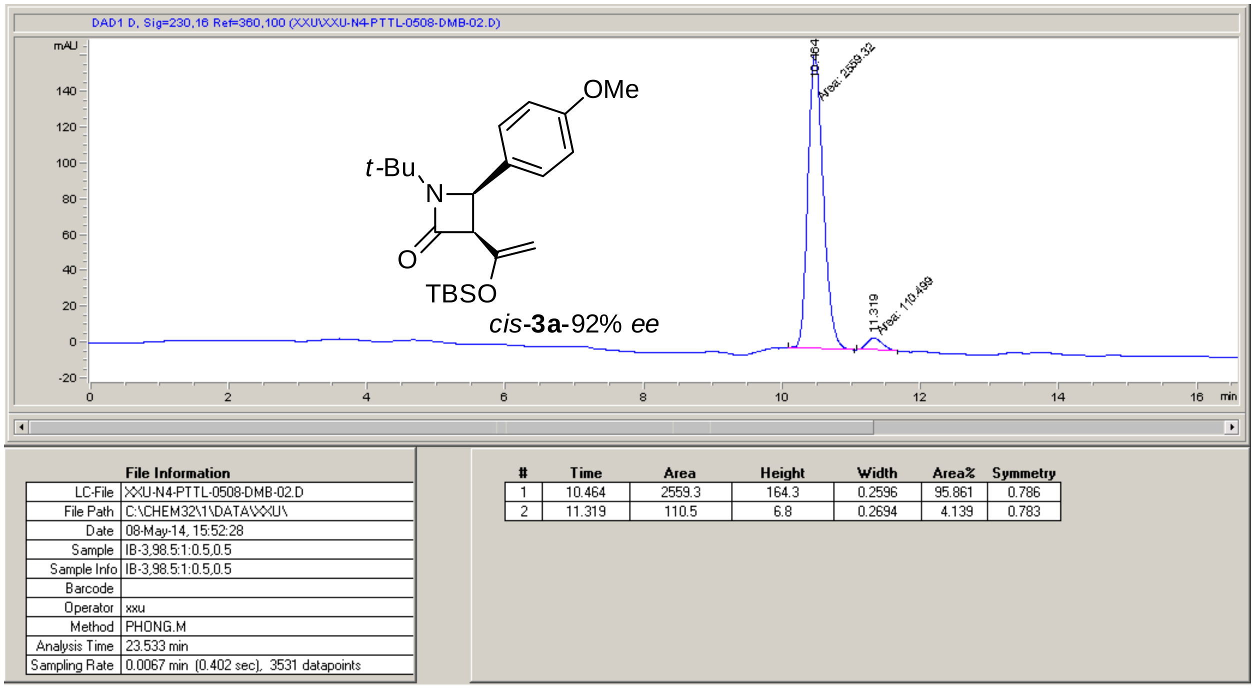

File Information	
LC-File	XXU-N4-PTTL-0508-DMB-02.D
File Path	C:\CHEM32\1\DATA\XXU\
Date	08-May-14, 15:52:28
Sample	IB-3,98.5:1:0.5,0.5
Sample Info	IB-3,98.5:1:0.5,0.5
Barcode	
Operator	xxu
Method	PHONG.M
Analysis Time	23.533 min
Sampling Rate	0.0067 min (0.402 sec), 3531 datapoints

#	Time	Area	Height	Width	Area%	Symmetry
1	10.464	2559.3	164.3	0.2596	95.861	0.786
2	11.319	110.5	6.8	0.2694	4.139	0.783

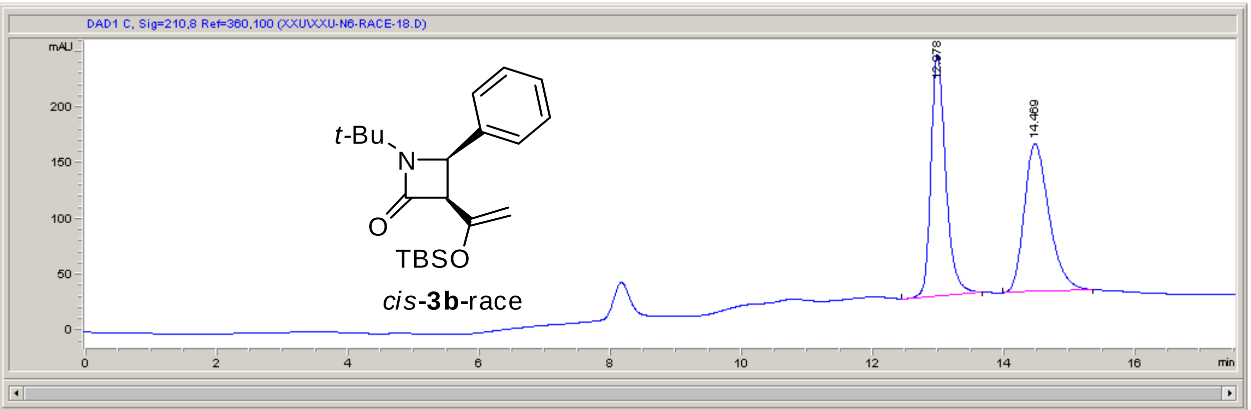

File Information	
LC-File	XXU-N6-RACE-18.D
File Path	C:\CHEM32\1\DATA\XXU\
Date	14-May-14, 17:11:38
Sample	ad-h, 99:1,0.4
Sample Info	ad-h, 99:1,0.4
Barcode	
Operator	XXU
Method	PHONG.M
Analysis Time	17.573 min
[illegible]	[illegible]

#	Time	Area	Height	Width	Area%	Symmetry
1	12.978	3431.9	217.3	0.2402	49.243	0.717
2	14.469	3537.5	133	0.4062	50.757	0.718

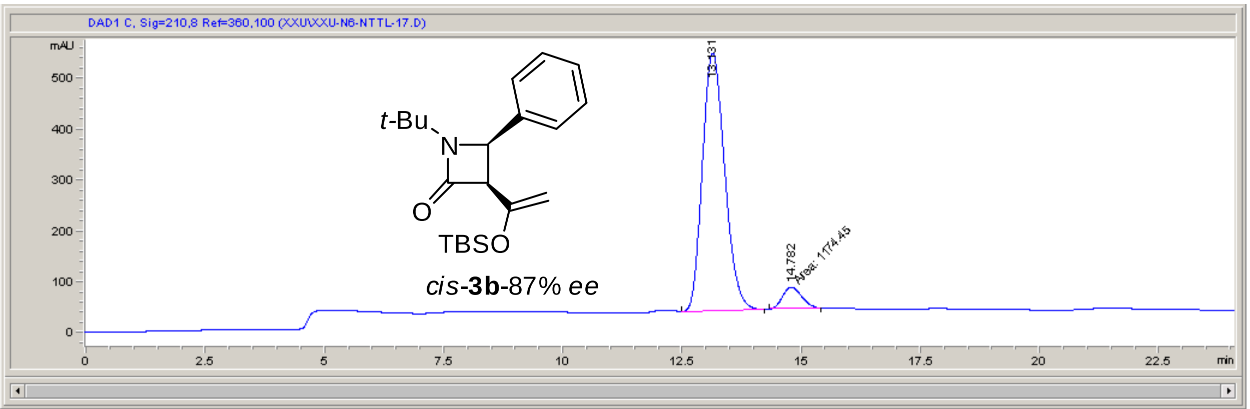

File Information	
LC-File	XXU-N6-NTTL-17.D
File Path	C:\CHEM32\1\DATA\XXU\
Date	13-May-14, 18:13:00
Sample	ad-h, 99:1,0.4
Sample Info	ad-h, 99:1,0.4
Barcode	
Operator	XXU
Method	PHONG.M
Analysis Time	24.133 min
[illegible]	[illegible]

#	Time	Area	Height	Width	Area%	Symmetry
1	13.131	16628.8	507.4	0.5103	93.403	0.8
2	14.782	1174.4	42.2	0.4641	6.597	0.81

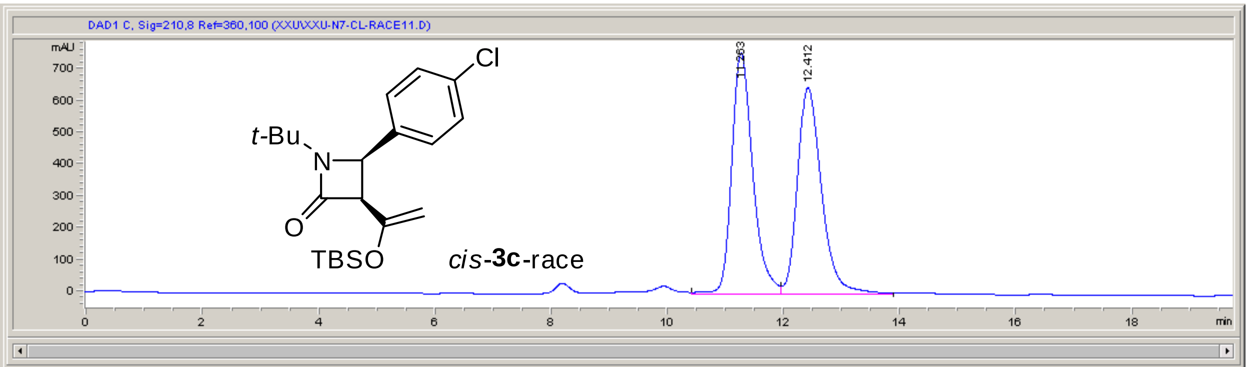

File Information	
LC-File	XXU-N7-CL-RACE11.D
File Path	C:\CHEM32\1\DATA\XXU\
Date	20-May-14, 19:27:05
Sample	aD-H, 98:2, 0.4
Sample Info	aD-H, 98:2, 0.4
Barcode	
Operator	XXU
Method	PHONG.M
Analysis Time	19.773 min
Sampling Rate	0.0067 min (0.402 sec), 2967 datapoints

#	Time	Area	Height	Width	Area%	Symmetry
1	11.263	19282.7	758.1	0.3887	50.702	0.754
2	12.412	18748.8	650.6	0.4442	49.298	0.702

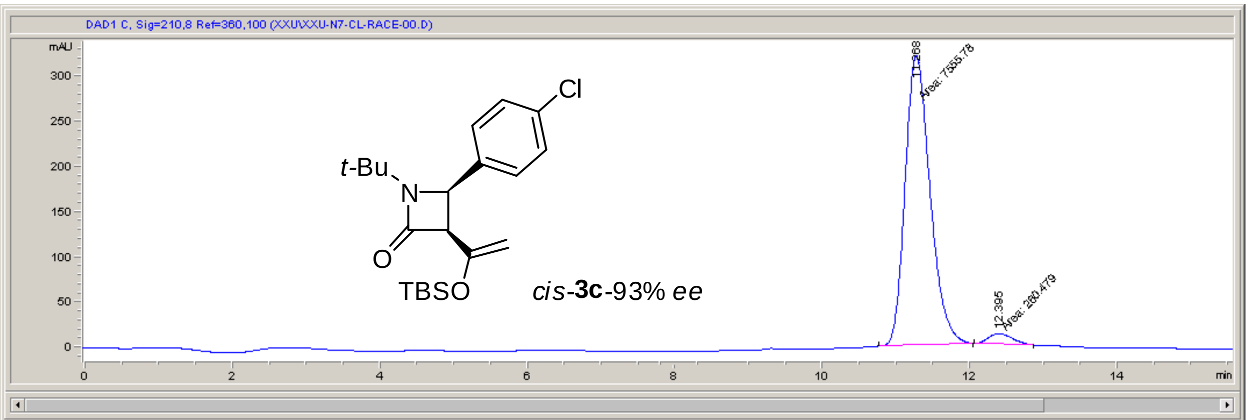

File Information	
LC-File	XXU-N7-CL-RACE-00.D
File Path	C:\CHEM32\1\DATA\XXU\
Date	20-May-14, 18:50:04
Sample	aD-H, 98:2, 0.4
Sample Info	aD-H, 98:2, 0.4
Barcode	
Operator	XXU
Method	PHONG.M
Analysis Time	18.287 min
Sampling Rate	0.0067 min (0.402 sec), 2744 datapoints

#	Time	Area	Height	Width	Area%	Symmetry
1	11.268	7555.8	320.4	0.3931	96.667	0.763
2	12.395	260.5	11.5	0.3768	3.333	0.769

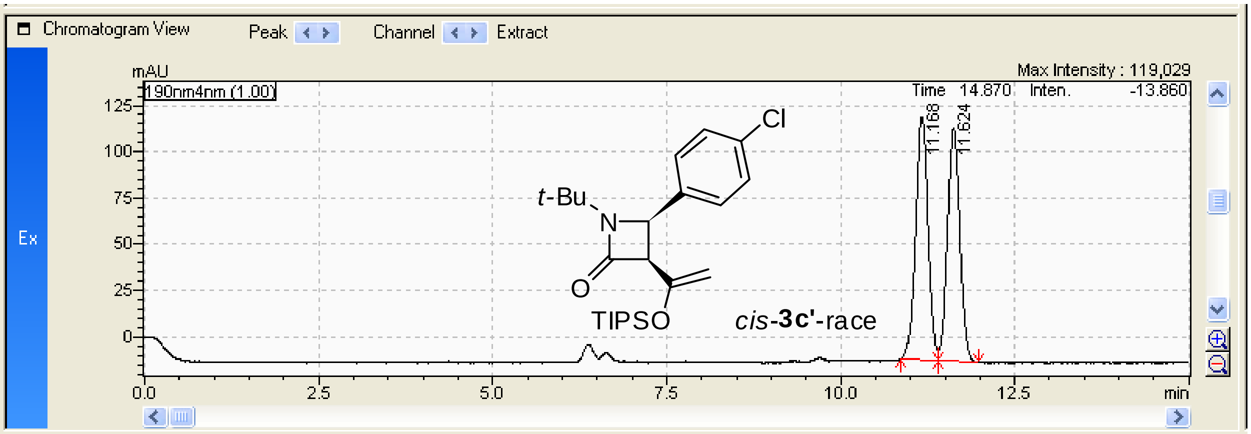

Results View - Peak Table

Peak Table | Compound | Group | Calibration Curve

Peak#	Ret. Time	Area	Area%	Conc.
1	11.168	1623042	50.998	50.998
2	11.624	1559520	49.002	49.002
Total		3182562	100.000	100.000

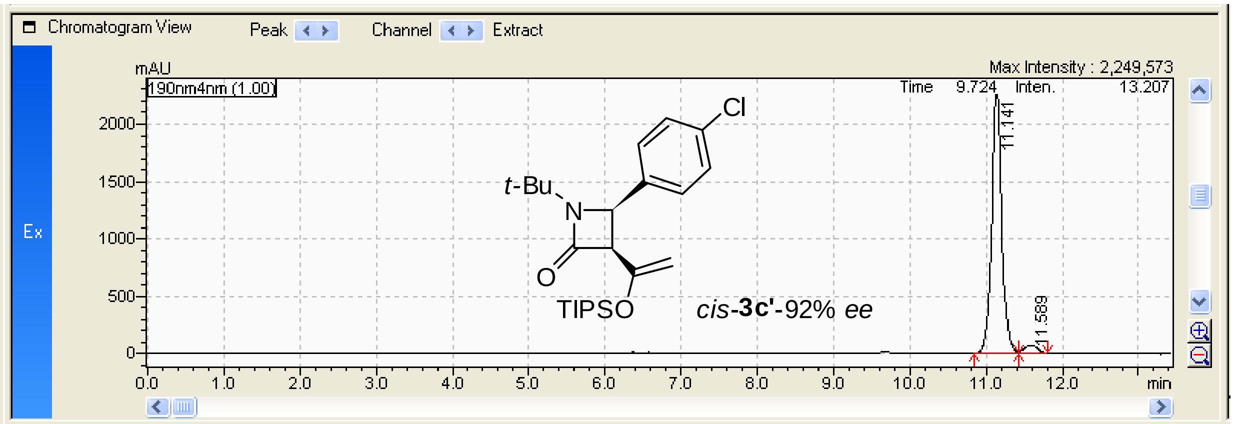

Results View - Peak Table

Peak Table | Compound | Group | Calibration Curve

Peak#	Ret. Time	Area	Area%	Conc.
1	11.141	21190672	95.917	95.917
2	11.589	902036	4.083	4.083
Total		22092708	100.000	100.000

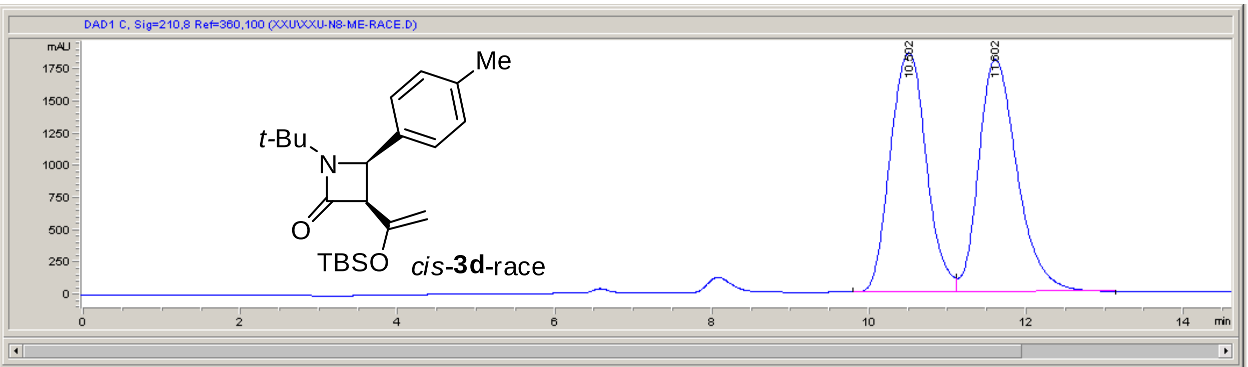

File Information	
LC-File	XXU-N8-ME-RACE.D
File Path	C:\CHEM32\1\DATA\XXU\
Date	22-May-14, 17:58:57
Sample	aD-H, 98:1;1, 0.5
Sample Info	aD-H, 98:1;1, 0.5
Barcode	
Operator	XXU
Method	PHONG.M
Analysis Time	17.52 min
Sampling Rate	0.0067 min (0.402 sec), 2629 datapoints

#	Time	Area	Height	Width	Area%	Symmetry
1	10.502	59417.1	1858.5	0.509	49.220	1.01
2	11.602	61300.8	1803	0.5267	50.780	0.713

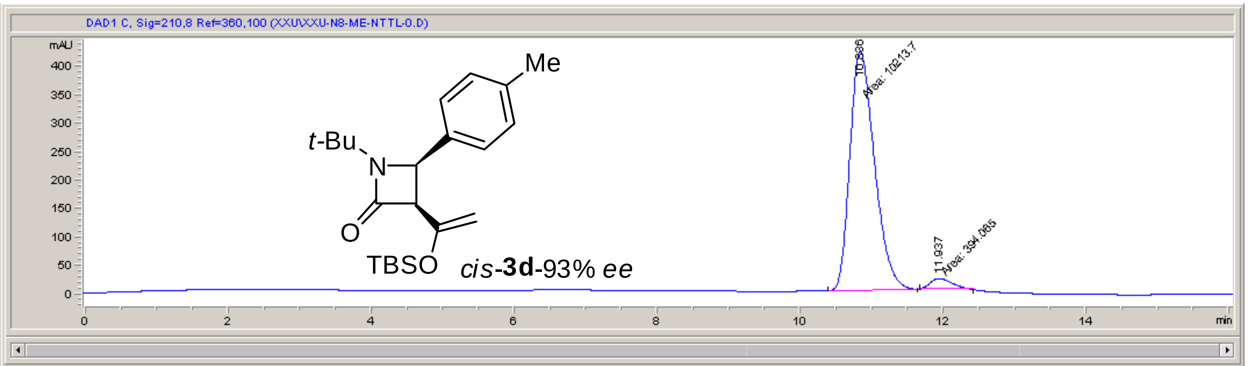

File Information	
LC-File	XXU-N8-ME-NTTL-0.D
File Path	C:\CHEM32\1\DATA\XXU\
Date	22-May-14, 19:56:45
Sample	aD-H, 99:0.5:0.5, 0.5
Sample Info	aD-H, 99:0.5:0.5, 0.5
Barcode	
Operator	XXU
Method	PHONG.M
Analysis Time	16.093 min
Sampling Rate	0.0067 min (0.402 sec), 2415 datapoints

#	Time	Area	Height	Width	Area%	Symmetry
1	10.836	10213.7	422.5	0.4029	96.285	0.705
2	11.937	394.1	18.5	0.3549	3.715	0.675

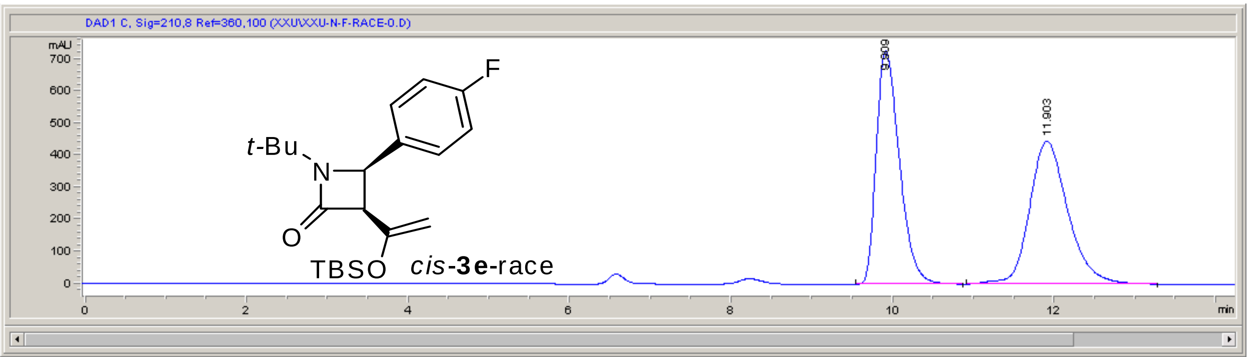

File Information

LC-File	XXU-N-F-RACE-0.D
File Path	C:\CHEM32\1\DATA\XXU\
Date	29-May-14, 17:11:09
Sample	aD-H, 99::0.5:0.5, 0.5
Sample Info	aD-H, 99::0.5:0.5, 0.5
Barcode	
Operator	XXU
Method	PHONG.M

#	Time	Area	Height	Width	Area%	Symmetry
1	9.909	14534.4	728.1	0.3109	49.596	0.649
2	11.903	14771.5	445.6	0.5047	50.404	0.784

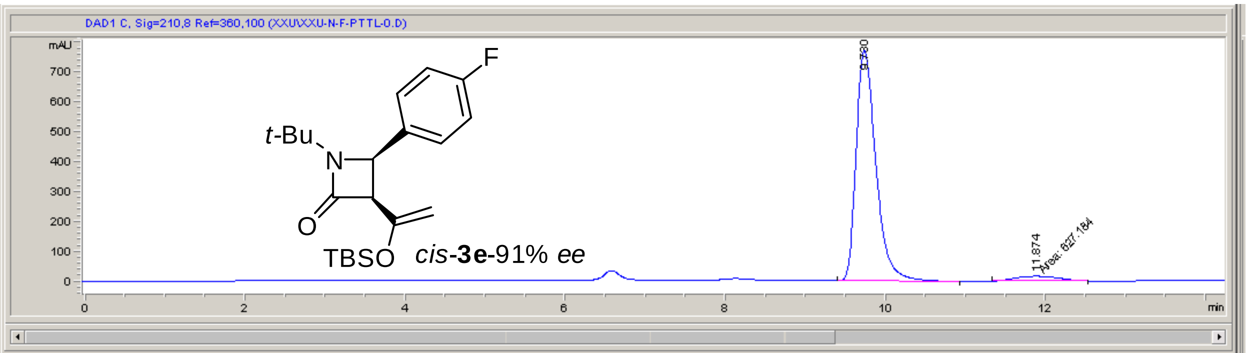

File Information

LC-File	XXU-N-F-PTTL-0.D
File Path	C:\CHEM32\1\DATA\XXU\
Date	29-May-14, 16:49:22
Sample	aD-H, 99::0.5:0.5, 0.5
Sample Info	aD-H, 99::0.5:0.5, 0.5
Barcode	
Operator	XXU
Method	PHONG.M

#	Time	Area	Height	Width	Area%	Symmetry
1	9.73	13227.2	769.4	0.2666	95.473	0.661
2	11.874	627.2	16.3	0.6414	4.527	0.921

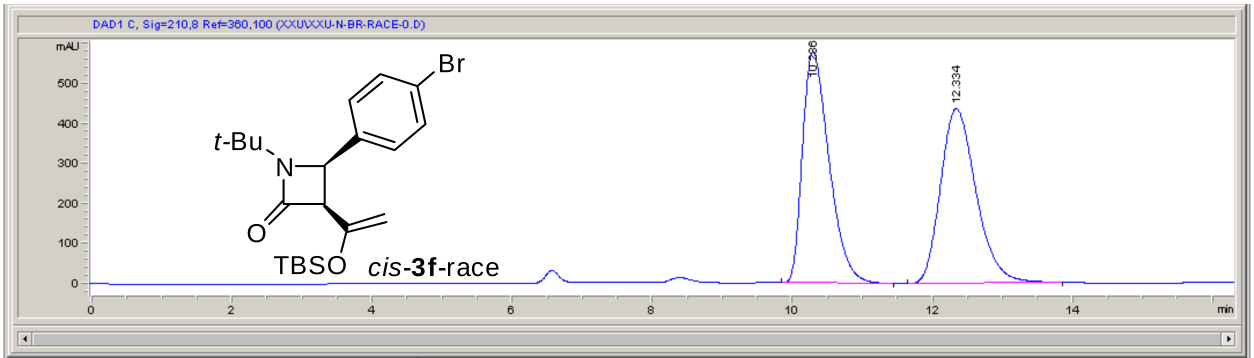

File Information	
LC-File	XXU-N-BR-RACE-0.D
File Path	C:\CHEM32\1\DATA\XXU\
Date	29-May-14, 15:39:36
Sample	aD-H, 99::0.5:0.5, 0.5
Sample Info	aD-H, 99::0.5:0.5, 0.5
Barcode	
Operator	XXU
Method	PHONG.M

#	Time	Area	Height	Width	Area%	Symmetry
1	10.286	15334.8	578.3	0.4113	49.764	0.602
2	12.334	15480	437.2	0.549	50.236	0.746

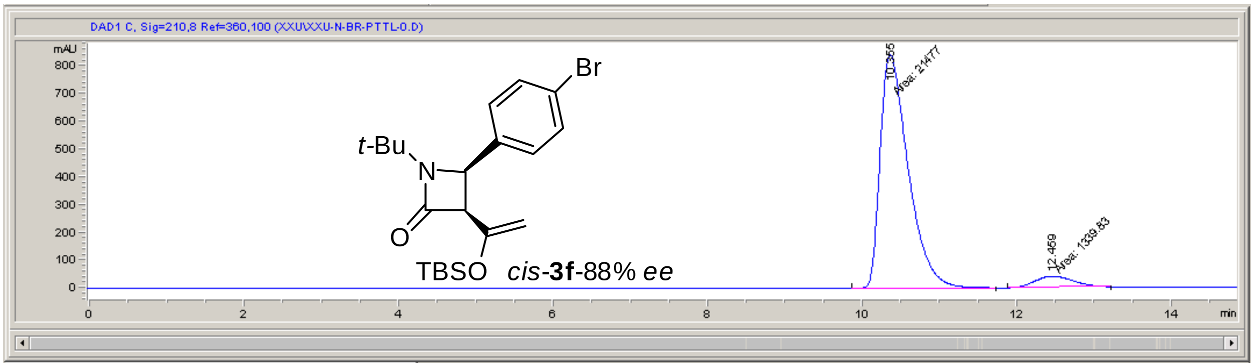

File Information	
LC-File	XXU-N-BR-PTTL-0.D
File Path	C:\CHEM32\1\DATA\XXU\
Date	29-May-14, 15:21:22
Sample	aD-H, 99::0.5:0.5, 0.5
Sample Info	aD-H, 99::0.5:0.5, 0.5
Barcode	
Operator	XXU
Method	PHONG.M

#	Time	Area	Height	Width	Area%	Symmetry
1	10.355	21477	843.9	0.4241	94.128	0.528
2	12.459	1339.8	39.5	0.5652	5.872	0.822

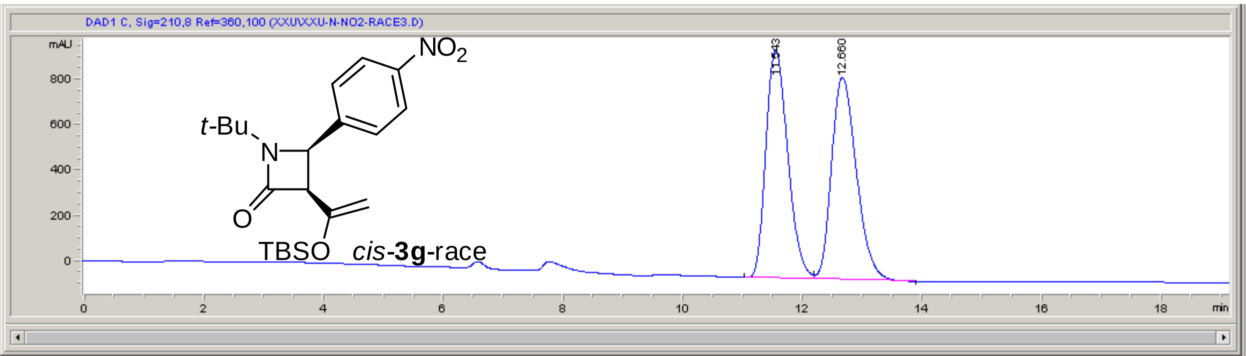

File Information	
LC-File	XXU-N-NO2-RACE3.D
File Path	C:\CHEM32\1\DATA\XXU\
Date	28-May-14, 15:40:12
Sample	aD-H, 98::0.5:1.5, 0.5
Sample Info	aD-H, 98::0.5:1.5, 0.5
Barcode	
Operator	XXU
Method	PHONG.M

#	Time	Area	Height	Width	Area%	Symmetry
1	11.543	25696.5	1007.5	0.4016	49.885	0.747
2	12.66	25814.5	893	0.4513	50.115	0.724

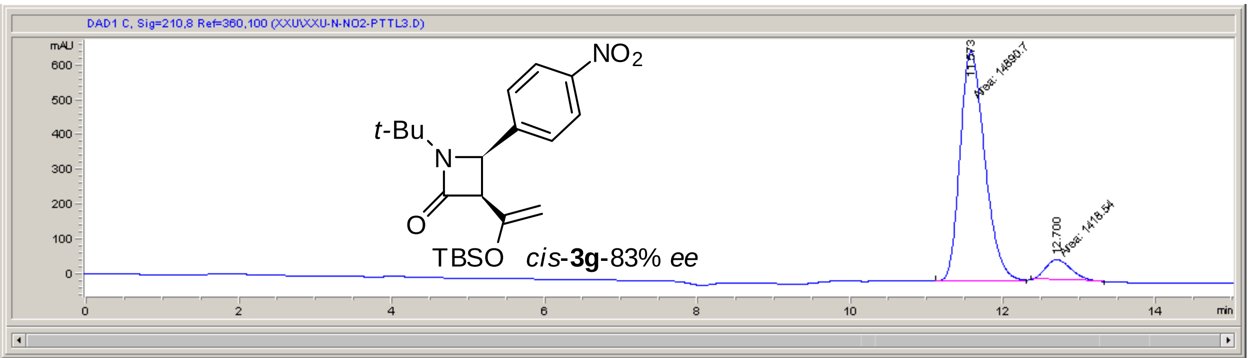

File Information	
LC-File	XXU-N-NO2-PTTL3.D
File Path	C:\CHEM32\1\DATA\XXU\
Date	28-May-14, 15:24:13
Sample	aD-H, 98::0.5:1.5, 0.5
Sample Info	aD-H, 98::0.5:1.5, 0.5
Barcode	
Operator	XXU
Method	PHONG.M

#	Time	Area	Height	Width	Area%	Symmetry
1	11.573	14890.7	661.1	0.3754	91.302	0.749
2	12.7	1418.5	59.2	0.3991	8.698	0.725

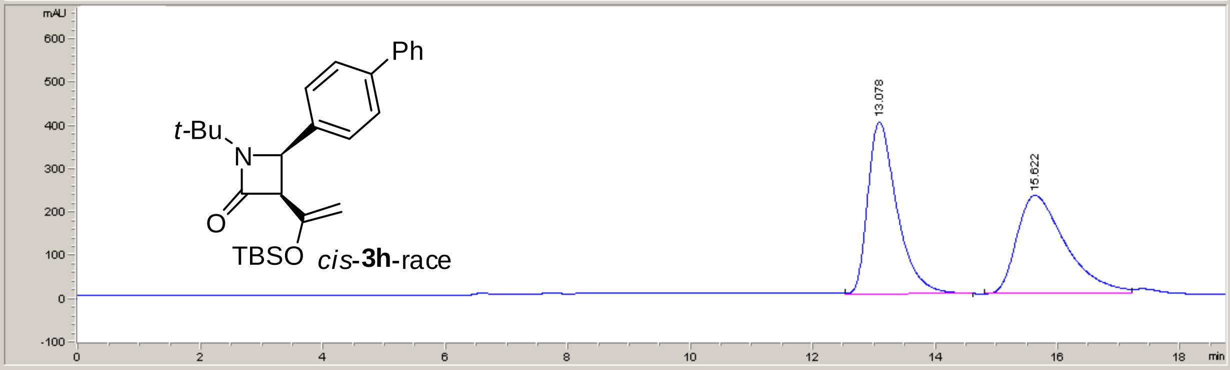

File Information	
LC-File	XXU-N-PH-RACE-32.D
File Path	C:\CHEM32\1\DATA\XXU\
Date	18-Jun-14, 13:03:36
Sample	aD-H, 98:1:1, 0.5
Sample Info	aD-H, 98:1:1, 0.5
Barcode	
Operator	XXU
Method	PHONG.M
Analysis Time	19.673 min
Sampling Rate	0.0067 min (0.402 sec), 2952 datapoints

#	Time	Area	Height	Width	Area%	Symmetry
1	13.078	13236.7	397.9	0.5021	51.033	0.605
2	15.622	12700.7	227.3	0.8539	48.967	0.541

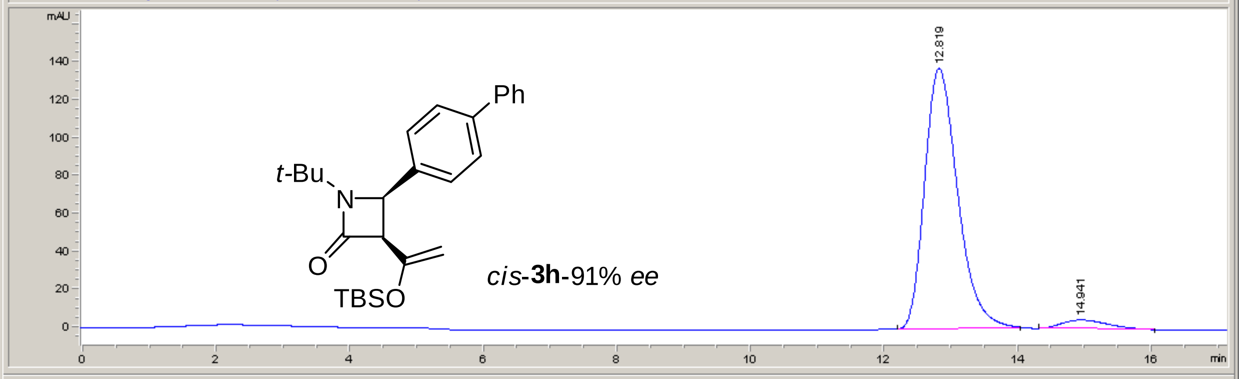

File Information	
LC-File	XXU-N-PH-PTTL-3.D
File Path	C:\CHEM32\1\DATA\XXU\
Date	18-Jun-14, 13:24:22
Sample	aD-H, 98:1:1, 0.5
Sample Info	aD-H, 98:1:1, 0.5
Barcode	
Operator	XXU
Method	PHONG.M
Analysis Time	28.587 min
Sampling Rate	0.0067 min (0.402 sec), 4289 datapoints

#	Time	Area	Height	Width	Area%	Symmetry
1	12.819	4734.2	137.4	0.532	95.394	0.73
2	14.941	228.6	4.7	0.6899	4.606	0.644

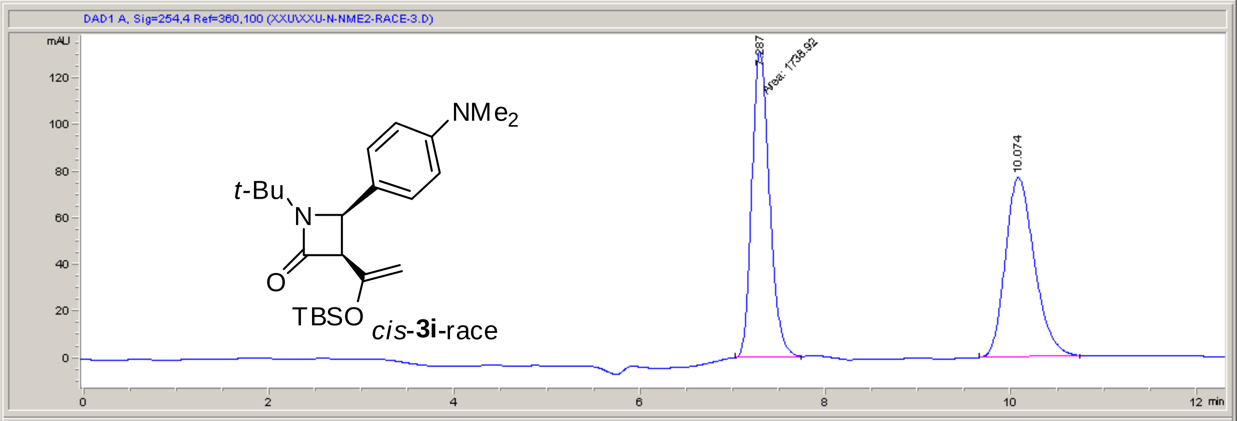

File Information	
LC-File	XXU-N-NME2-RACE-3.D
File Path	C:\CHEM32\1\DATA\XXU\
Date	20-Jun-14, 11:47:24
Sample	OD-H, 98:2, 0.7
Sample Info	OD-H, 98:2, 0.7
Barcode	
Operator	XXU
Method	PHONG.M
Analysis Time	26.927 min
Sampling Rate	0.0067 min (0.402 sec), 4040 datapoints

#	Time	Area	Height	Width	Area%	Symmetry
1	7.287	1738.9	131.6	0.2203	50.771	0.799
2	10.074	1686.1	77	0.3374	49.229	0.761

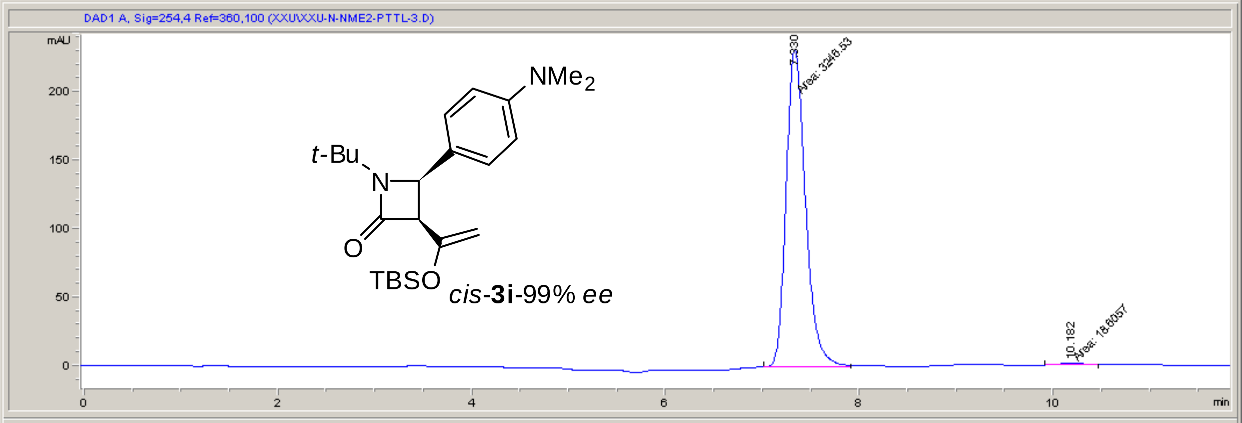

File Information	
LC-File	XXU-N-NME2-PTTL-3.D
File Path	C:\CHEM32\1\DATA\XXU\
Date	20-Jun-14, 11:32:03
Sample	OD-H, 98:2, 0.7
Sample Info	OD-H, 98:2, 0.7
Barcode	
Operator	XXU
Method	PHONG.M
Analysis Time	11.867 min
Sampling Rate	0.0067 min (0.402 sec), 1781 datapoints

#	Time	Area	Height	Width	Area%	Symmetry
1	7.33	3246.5	231.5	0.2337	99.430	0.772
2	10.182	18.6	1.1	0.2935	0.570	1.002

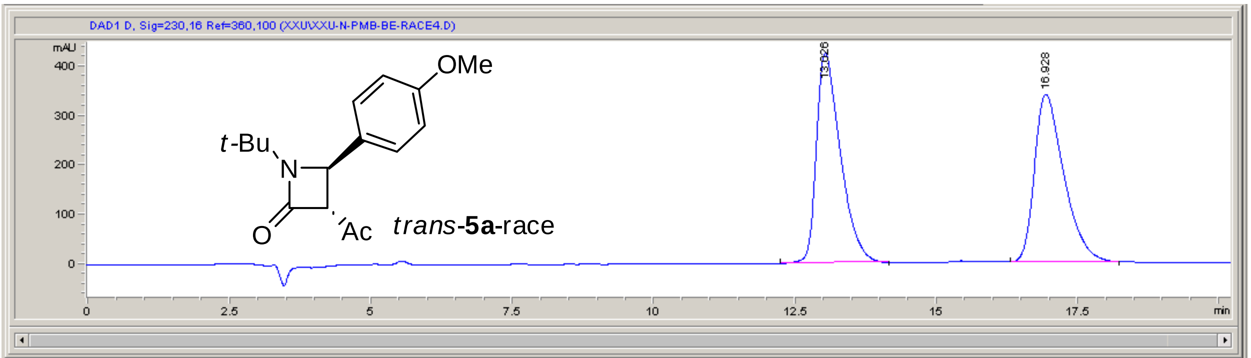

File Information

LC-File	XXU-N-PMB-BE-RACE4.D
File Path	C:\CHEM32\1\DATA\XXU\
Date	03-Jun-14, 14:28:21
Sample	aD-H, 95:5, 1.0
Sample Info	aD-H, 95:5, 1.0
Barcode	
Operator	XXU
Method	PHONG.M

#	Time	Area	Height	Width	Area%	Symmetry
1	13.026	12584.9	424.4	0.4439	50.700	0.542
2	16.928	12237.4	338	0.5484	49.300	0.584

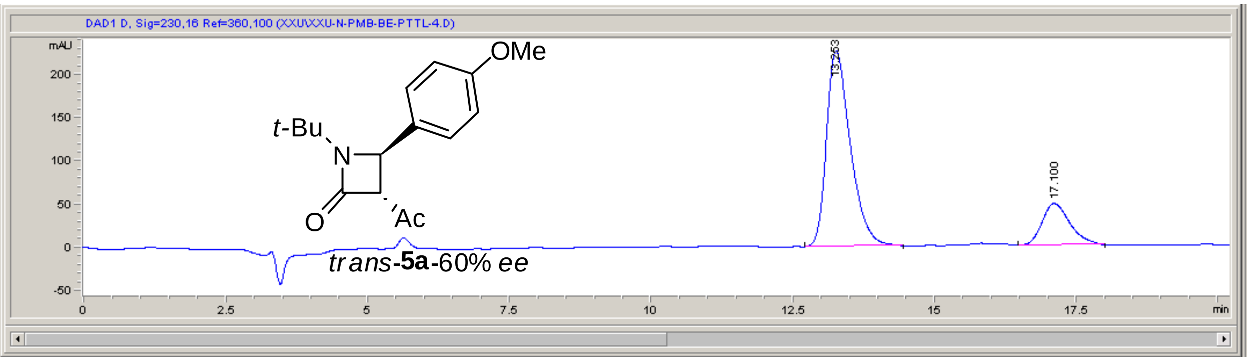

File Information

LC-File	XXU-N-PMB-BE-PTTL-4.D
File Path	C:\CHEM32\1\DATA\XXU\
Date	03-Jun-14, 14:50:10
Sample	aD-H, 95:5, 1.0
Sample Info	aD-H, 95:5, 1.0
Barcode	
Operator	XXU
Method	PHONG.M

#	Time	Area	Height	Width	Area%	Symmetry
1	13.253	6647	226	0.4451	80.124	0.623
2	17.1	1648.9	48	0.5287	19.876	0.715

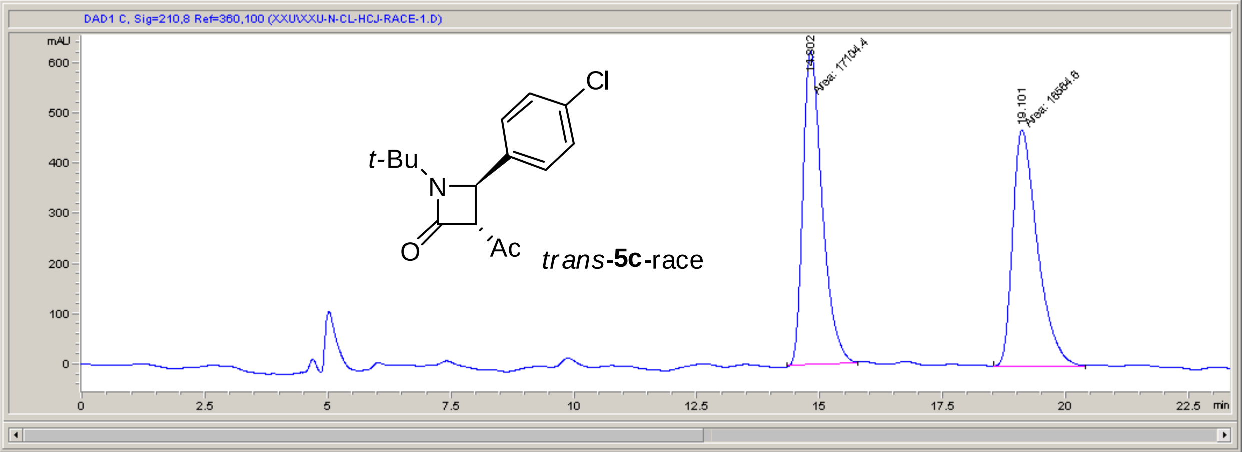

File Information

LC-File	XXU-N-CL-HCJ-RACE-1.D
File Path	C:\CHEM32\1\DATA\XXU\
Date	10-Jul-14, 17:38:47
Sample	aD-H 97:3, 0.7
Sample Info	aD-H 97:3, 0.7
Barcode	
Operator	XXU
Method	PHONG.M
Analysis Time	41.04 min
Sampling Rate	0.0067 min (0.402 sec), 6157 datapoints

#	Time	Area	Height	Width	Area%	Symmetry
1	14.802	17104.4	624.1	0.4568	50.802	0.639
2	19.101	16564.6	470.2	0.5871	49.198	0.579

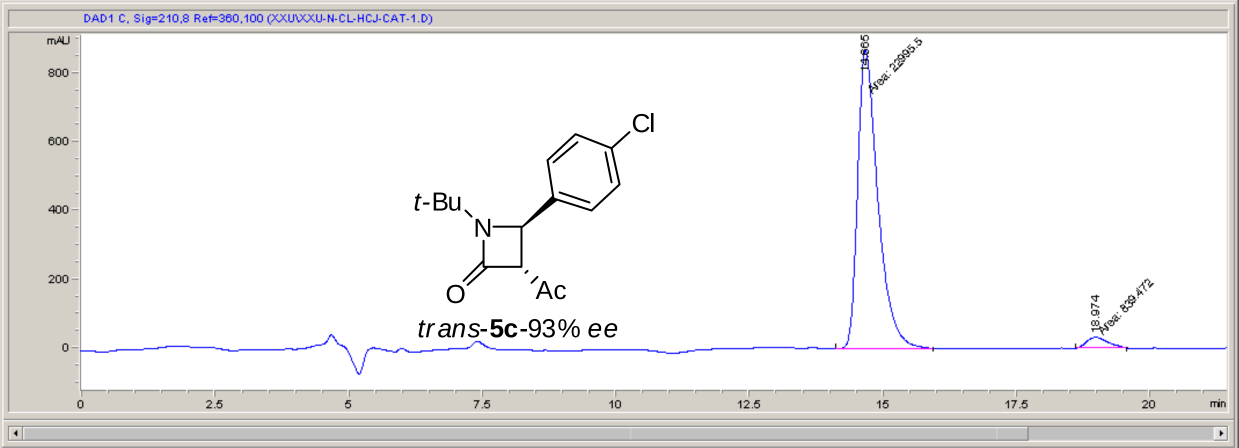

File Information

LC-File	XXU-N-CL-HCJ-CAT-1.D
File Path	C:\CHEM32\1\DATA\XXU\
Date	10-Jul-14, 17:10:23
Sample	aD-H 97:3, 0.7
Sample Info	aD-H 97:3, 0.7
Barcode	
Operator	XXU
Method	PHONG.M
Analysis Time	25.433 min
Sampling Rate	0.0067 min (0.402 sec), 3816 datapoints

#	Time	Area	Height	Width	Area%	Symmetry
1	14.665	22995.5	868.3	0.4414	96.478	0.597
2	18.974	839.5	31	0.4516	3.522	0.73

Data Collection and Structure Refinement for (3*S*,4*R*)-3i (UM-2600).

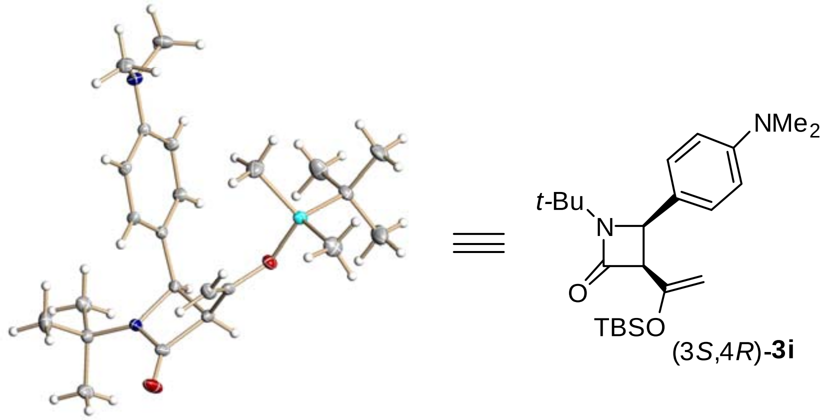

A colorless prism-like specimen of $C_{23}H_{38}N_2O_2Si$, approximate dimensions 0.41 mm × 0.49 mm × 0.52 mm, was used for the X-ray crystallographic analysis. The X-ray intensity data were measured on a Bruker APEX-II CCD system equipped with a graphite monochromator and a MoKα sealed tube (λ = 0.71073 Å). Data collection temperature was 150 K.

The total exposure time was 5.29 hours. The frames were integrated with the Bruker SAINT software package using a narrow-frame algorithm. The integration of the data using an orthorhombic unit cell yielded a total of 19657 reflections to a maximum θ angle of 30.00° (0.71 Å resolution), of which 6987 were independent (average redundancy 2.813, completeness = 99.9%, R_{int} =2.53%, R_{sig} = 3.97%) and 6686 (95.69%) were greater than $2\sigma(F^2)$. The final cell constants of a = 12.0041(6) Å, b = 12.0054(6) Å,c = 16.8057(8) Å, V = 2421.9(2) $Å^3$, are based upon the refinement of the XYZ-centroids of 9972 reflections above 20 σ(I) with4.798° < 2θ < 61.12°. Data were corrected for absorption effects using the multi-scan method (SADABS). The calculated minimum and maximum transmission coefficients (based on crystal size) are 0.9421 and 0.9540.

The structure was solved and refined using the Bruker SHELXTL Software Package, using the space group P 21 21 21, with Z = 4 for the formula unit, $C_{23}H_{38}N_2O_2Si$. The final anisotropic full-matrix least-squares refinement on F^2 with 370 variables converged at R_1 = 3.07%, for the observed data and wR_2 = 6.41% for all data. The goodness-of-fit was 1.000. The largest peak in the final difference electron density synthesis was 0.287 $e^-/Å^3$ and the largest hole was -0.153 $e^-/Å^3$ with an RMS deviation of 0.033 $e^-/Å^3$. On the basis of the final model, the calculated density was 1.104 g/cm^3 and F(000), 880 e^-.
